# Analysis of eIF4E-family members in fungi contributes to their classification in eukaryotes

**DOI:** 10.1016/j.jbc.2024.108129

**Published:** 2024-12-21

**Authors:** Greco Hernández, Daniela Ross-Kaschitza, Gabriel Moreno-Hagelsieb, Alejandra García, Dora Emma Vélez, Blanca Licia Torres

**Affiliations:** 1 mRNA and Cancer Laboratory, Unit of Biomedical Research on Cancer, National Institute of Cancer (INCan), Mexico City, Mexico; 2 Escuela de Medicina y Ciencias de la Salud, Tecnológico de Monterrey, Mexico City, Mexico; 3 Institut für Biochemie und Molekulare Medizin (IBMM), University of Bern, Bern, Switzerland; 4 Department of Biology, Wilfrid Laurier University, Waterloo, Ontario, Canada

**Keywords:** eIF4E, fungi, gene expression, mRNA, RNA metabolism, translation initiation

## Abstract

The kingdom of fungi contains highly diverse species. However, fundamental processes sustaining life such as RNA metabolism are much less comparatively studied in Fungi than in other kingdoms. A key factor in the regulation of mRNA expression is the cap-binding protein eIF4E, which plays roles in mRNA nuclear export, storage, and translation. The advent of massive genomics has unveiled a constellation of eIF4E-family members across eukaryotes. However, how this protein diverged into fungal species remains largely unexplored. Here, we studied the genome of 538 species from six evolutionarily distant *phyla* and retrieved 1462 eIF4E cognates. The analyzed species contained 1 to 7 paralogs. We sorted all cognates in six phylogenetically coherent clades, that we termed Class I to VII (mammalian Class III was absent in Fungi). Proteins from Classes IV to VII did not match the current eIF4Es classification that is based on variations in the residues equivalent to W43 and W56 of the human protein. eIF4Es from other eukaryotes do not fit into this classification either. Thus, we have updated the eIF4E categorization based on cladistics and the presence of cap-binding amino acids to better fit eIF4E’s diversity across eukaryotes. Finally, we predicted the structure of the global protein and the cap-binding pocket and experimentally tested the ability to rescue the lack of endogenous eIF4E in *Saccharomyces cerevisiae* of representative members of each of the six classes of fungal eIF4E.

The eukaryotic-specific cap-binding protein translation initiation factor 4E (eIF4E) is a key player in RNA metabolism. By recognizing the cap structure of mRNAs, eIF4E is involved in translation and mRNA storage in cytoplasmic foci (1, 2). Besides the cap-binding complex (CBP)20/CBP80, human eIF4E in complex either with Importin 8 (Imp8) or human homeodomain protein 9 (HOXA9) also catalyzes mRNA nuclear export (3). eIF4E is defined by a unique α/β fold of three alpha-helices and six beta-sheets (4). It consists of a core of 160 to 170 amino acids carrying eight conserved tryptophan residues, *i.e.*, W43, W46, W56, W73, W102, W113, W130, and W166 (human protein numbering). Structural studies showed that eIF4E is a cupped-hand-shaped protein whose pocket interacts with the cap structure between W56 and W102 *via* sandwich-like cation-π stacking interactions and W166 recognizes the cap methyl group. Additional salt bridge interactions of the positively charged R112, R157, and K162 with the ribose and triphosphate moieties, and between E103 and the guanine ring further stabilize the association with the cap (5, 6, 7).

The advent of intensive genomics has revealed a constellation of eIF4E family members across eukaryotes (8). Among them, mammalian eIF4E-1, terrestrial plants eIF4E (p26), and *Saccharomyces cerevisiae* eIF4E are the prototypical proteins. By analyzing 220 eIF4E homologs from 118 species, primarily Metazoan, and plants, a classification of the eIF4E-family members was established 20 years ago (9) according to variations in the residues equivalent to W43 and W56 and sorted out the proteins into three classes. Class I cognates contain both W residues; Class II members, termed eIF4E homologous protein (4EHP), possess Y, F, or L at the first position and Y or F at the second position; and Class III proteins possess W at the first position and C or Y at the second position. Interestingly, functional diversification has been described for several eIF4E cognates (1).

Despite its paramount importance in the biosphere, food industry, agriculture, and medicine, the kingdom of Fungi remains largely unexplored in this regard. Indeed, fungal eIF4E has been only studied in the yeasts *S. cerevisiae* (6, 10, 11, 12, 13) and *Schizossacharomyces pombe* (14, 15), of the *phylum Ascomycota*. *In silico* studies have also identified Class I *eIF4E* orthologs in 36 unicellular species of this *phylum* (9, 16, 17). Here, we have analyzed the divergence of eIF4E across Fungi. We identified six separate clades of proteins belonging to six *phyla* and expanded the eIF4Es classification based on cladistics and the type of amino acids key for cap binding that better accounts for the complex diversity of eIF4Es across eukaryotes. Finally, the fungal orthologs showed different functional features.

## Results

**Figure 1:**
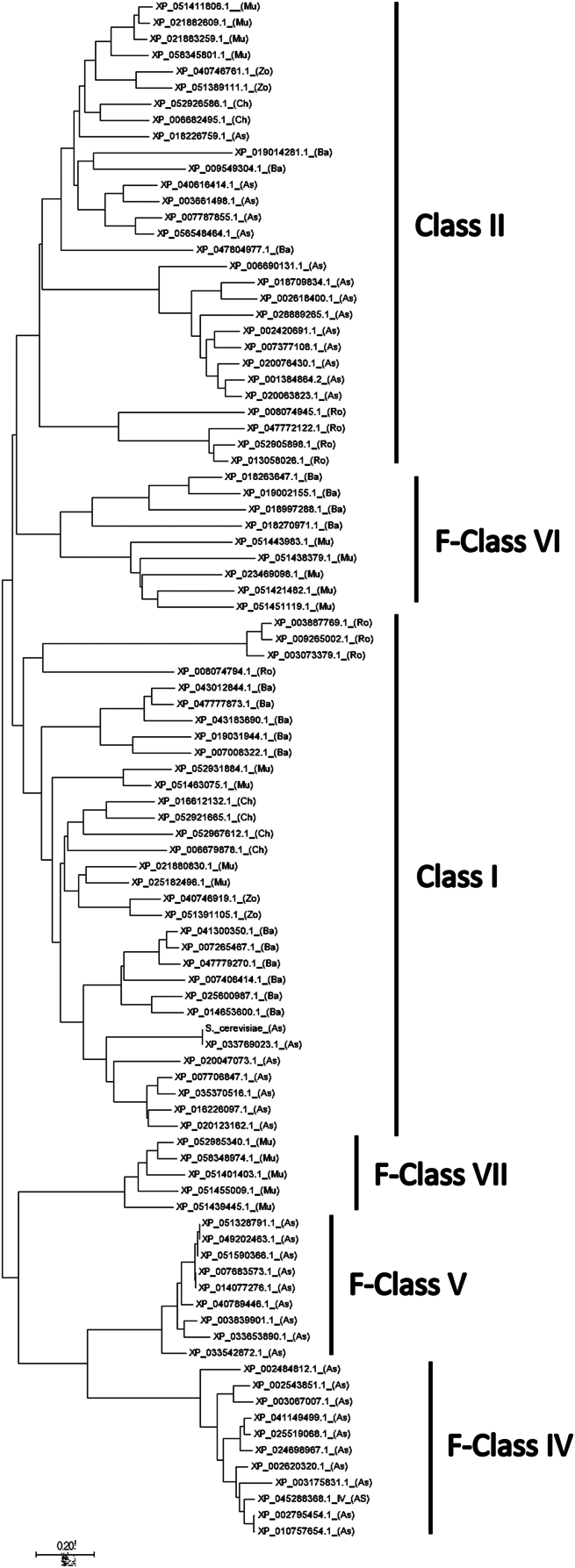
**Cladogram showing the phylogenetic relationship of fungal eIF4Es.** Full-length sequences of selected eIF4E-family members from six *phyla* were analyzed by the Neighbor-Joining method. The proteins are distributed in six clades of eIF4E. The Fungi-specific classes are indicated with a F prefix. *Phylum* name is indicated in parenthesis: (As)*, Ascomycota*; (Ro), *Rozellomycota*; (Ba), Basidiomycota; (Ch), *Chytridiomycota*; (Zo), Zoopagomycota; (Mu), Mucoromycota.

We identified 1462 eIF4E cognates belonging to 538 fungal species in the genomes available at RefSeq (18). We retrieved *eIF4E* genes from the *phyla Ascomycota*, *Basidiomycota*, *Chytridiomycota*, *Rozellomycota*, *Mucoromycota,* and *Zoopagomycota* (19) (Table S1). The statistics of the acquired cognates are presented in Table 1. A cladistic analysis sorted all fungal eIF4Es into six clades (Fig. 1). We found that the fungal species contained between 1 and 7 paralogs. Cladograms of the individual *phyla* are shown in Figures S1–S6.

**Table 1:** *eIF4E* cognate genes identified in six fungal *phyla*

*Phylum*	Species analyzed	*eIF4E* paralogs per species	Class
*Ascomycota*	421	1–4	Class I
			Class II
			F-Class IV
			F-Class V
*Basidiomycota*	82	1–5	Class I
			F-Subclass IA
			Class II
			F-Class VI
*Chytridiomycota*	5	2	Class I
			Class II
*Rozellomycota*	14	2–3	Class I
			F-Subclass IB
			Class II
*Mucoromycota*	14	1–7	Class I
			Class II
			F-Class VI
			F-Class VII
*Zoopagomycota*	2	2	Class I
			Class II
Total	538	1458	6

Alignment of the cognates from the six fungal *phyla* showed a group of eIF4E orthologs that possessed conserved residues that interact with the mRNA cap of the human protein. These orthologs shared high similarity to the core of prototype eIF4E (Class I) from other kingdoms (Fig. S7). All fungal *phyla* also contained one or more paralogs matching Class II proteins from Metazoan and terrestrial plants (20) (Fig. S8). However, no fungal orthologs fit into Class III. Other fungal eIF4E cognates, however, showed either conservative or non-conservative variations in different amino acids key for cap binding. Thus, many of these proteins did not fit into the current classification and conformed to novel fungal-specific groups of eIF4E that we termed F-Classes and F-Subclasses (F stands for fungal). For further comparisons, we used eIF4E from the two fungal species so far characterized, namely *S. cerevisiae* (12, 21) and *Schizosaccaromyces pombe* (14, 15) (Class I proteins).

**Figure 2:**
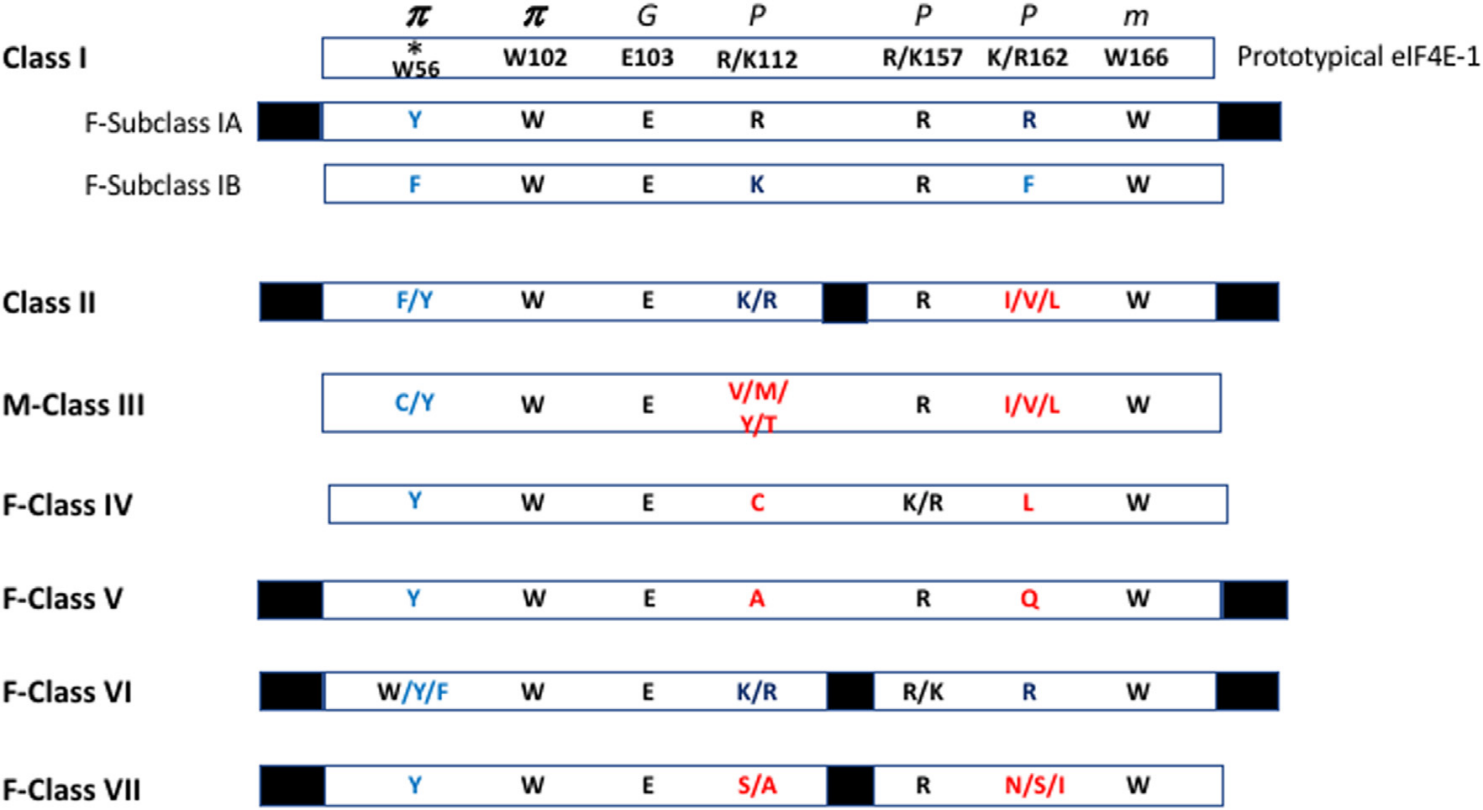
**Scheme representing the core of prototypical eIF4E and the classes of fungal eIF4Es**. Class I eIF4E from metazoan, terrestrial plants and yeast is represented. Mammalian Class III and fungal-specific classes of eIF4E are indicated with the prefix M- and F-, respectively. Numbering is from human protein (43). Amino acid residues directly contacting the mRNA cap structure are indicated: π, residues binding the guanine by π – π interactions; *G*, residue recognizing the guanine ring; *P*, positively charged residues interacting with the phosphate groups; *m*, W recognizing the ^7^methyl group (5, 6, 7, 13). Conservative substitutions with respect to the prototypical proteins are in blue. Non-conservative changes are in red. An asterisk indicates W56 used to classify the eIF4E-family members into three classes (9). Stretches of amino acid insertions and extensions are depicted as black boxes (not to scale).

Figures S9–S12 show alignments of *Ascomycota* proteins. These species were endowed with 1 to 4 eIF4E paralogs belonging to Class I and or Class II. Some eIF4E cognates showed changes in the positions corresponding to W56 (human protein numbering) ➜ Y, R/K112 ➜ C or A, and K/R162 ➜ L or Q conforming novel clades of cognates that we termed F-Classes IV and V, respectively. Some proteins contained amino acid stretches inserted between structural regions with respect to the prototypical eIF4Es (Fig. 2). The *phylum Basidiomycota* contained species endowed with 1 to 5 eIF4E cognates belonging to four clades of proteins, namely Class I (Fig. S13), Class II (Fig. S14), and the novel F-Class VI (Fig. S15). Some Class I paralogs had a W56 ➜ Y substitution, 14 to 29 amino acids stretch insertion, and large amino acid extensions ranging from 126 to 307 and 202 to 437 amino acids in both the amino and carboxy terminus, respectively, conforming a subclade we termed F-Subclass IA. Class VI proteins contained residues W/Y/F at position W56, insertions of 18 to 25 amino acids, and extensions ranging from 100 to 900 amino acids at the amino terminus and 160 to 850 amino acids at the carboxy terminus compared to human eIF4E-1.

The *phylum Chytridiomycota* (Fig. S16) possessed only Class I and Class II eIF4Es. The *phylum Rozellomycota* (Fig. S17) is endowed with Class I and Class II eIF4E orthologs. Some proteins contained 50 and 58 amino acids extensions at the amino and carboxy terminus, and some others have insertions of 22 amino acids stretches. Moreover, some Class I paralogs had W56 ➜ F and K/R162 ➜ F substitutions, conforming a subclade we termed F-Subclass IB. The *phylum Mucoromycota* contained species endowed with 1 to 7 eIF4Es, the largest number of paralogs among fungi. In addition to Class I (Fig. S18) and Class II (Fig. S19) eIF4Es, *Mucoromycota* contained eIF4Es belonging to the fungal F-Class VI and F-Class-VII (Fig. S20). Class VII contained the change W56 ➜ Y, the non-conservative substitutions at R/K112 ➜ S/A and K/R162 ➜ N/S/I, an amino acid stretch insertion and a large amino acids extension at the amino terminus (Fig. 2). From the *phylum Zoopagomycota* we retrieved only two Class I paralog and two Class II paralogs (Fig. S7 and S8, respectively).

Sequence comparisons of eIF4E from all fungal classes (Fig. 2) showed that, among the amino acids interacting with the cap structure of mRNA, those equivalent to human W102, E103, R/K157, and W166 positions were conserved across fungi. In contrast, W56 showed conservative substitutions toward F or Y, and R/K112 and K/R162 showed a high variability toward non-conservative amino acids. No fungal orthologs of the Metazoan Class III were found.

### Partial conservation of phosphorylatable residues across fungal eIF4E paralogs

eIF4E phosphorylation has been reported in different species. In *S. cerevisiae*, eIF4E residues S2 and S15 phosphorylate during log- and stationary phases of growth and also under heat shock (22). Studer *et al.* (23) further identified S28 as a phosphorylation site conserved across several species of fungi. Indeed, S28 phosphorylation and the phosphomimetic S28 E change were demonstrated to increase eIF4G affinity (23). Phosphorylated S209 of human/mouse eIF4E (24, 25) and its equivalent S251 in *Drosophila* eIF4E-1 (26) are key for eIF4E function and cell growth. We analyzed the conservation of *S. cerevisiae* S2, S15, and S28 and of the mammalian S209 residues across the fungal eIF4E paralogs (Table S2). S209 of mammalian eIF4E is the most conserved among the identified phosphorylatable residues, being highly conserved in Class I and Class II proteins from all the six fungal *phyla* analyzed, as well as in F-Class V. In contrast, it is absent in eIF4Es from F-Class IV, F-Class VI, and F-Class VII. F-Class VI and F-Class VII proteins lack all known phosphorylatable residues. Moreover, *Rozellomycota* Class I and Class II paralogs lack residues S15 and S28, and *Zoopagomycota* Class II proteins lack residues S2, S15, and S28.

### Three-dimensional analysis shows diverse structures among fungal eIF4Es

**Figure 3:**
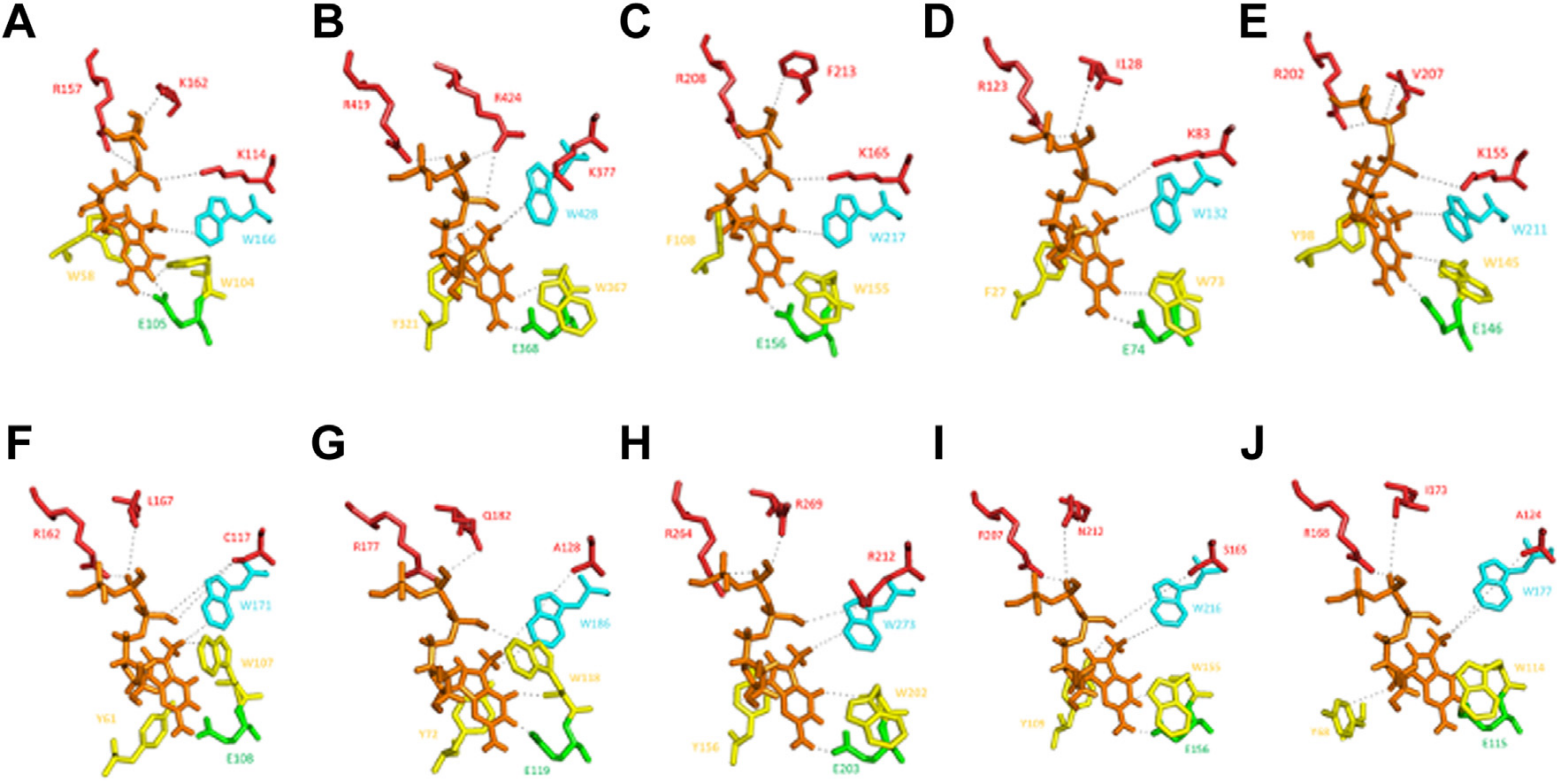
**Three-dimensional prediction of eIF4E amino acids involved in cap recognition.** eIF4E structures were predicted with Alphafold2. Only close-ups of the cap-binding pocket are shown. The cap is shown in orange and all amino acids involved in cap-interaction are highlighted in color-code. *Yellow*, pi-pi interaction; *Green*, guanine binding; *Red*, phosphate binding; *Blue*, ^7^methyl binding. *A*, *S. cerevisiae*, Class I (PDB structure 6FC1). *B*, *Cryptococcus gattii*, F-Subclass IA. *C*, *Mitosporidium daphnia*, F-Sublcass IB. D, *Nematocida major*, Class II. *E*, *Punctularia strigosozonata*, Class II. *F*, *Uncinocarpus reesii*, F-Class IV. *G*, *Ascochyta rabiei*, F-Class V. *H*, *Malassezia globosa*, F-Class VI. *I*, *Gilbertella persicaria*, F-Class VII. *J*, *Gamisella multidivaricata*, F-Class VII.

To gain insight into eIF4Es function, we performed Alphafold three-dimensional structure predictions of representative proteins of each fungal class, based on the known tertiary structure of the *S. cerevisiae* eIF4E in complex with the m^7^GTP (11) (Fig. 3). Close-up modeling showed that the cap-binding pocket is conserved among proteins, although it showed some variations in the spatial arrangement of the amino acids involved in cap-binding. Predicted global three-dimensional structures (Figs. S21–S25) showed that there is a core with the cupped-hand shape highly conserved. However, different protein extensions provoke significant structural variability. Further, the predicted distances between the cap-interacting amino acids and the cap were determined (Table S3).

### eIF4E from fungal classes differentially cross-complement *S. cerevisiae* CDC33

**Figure 4:**
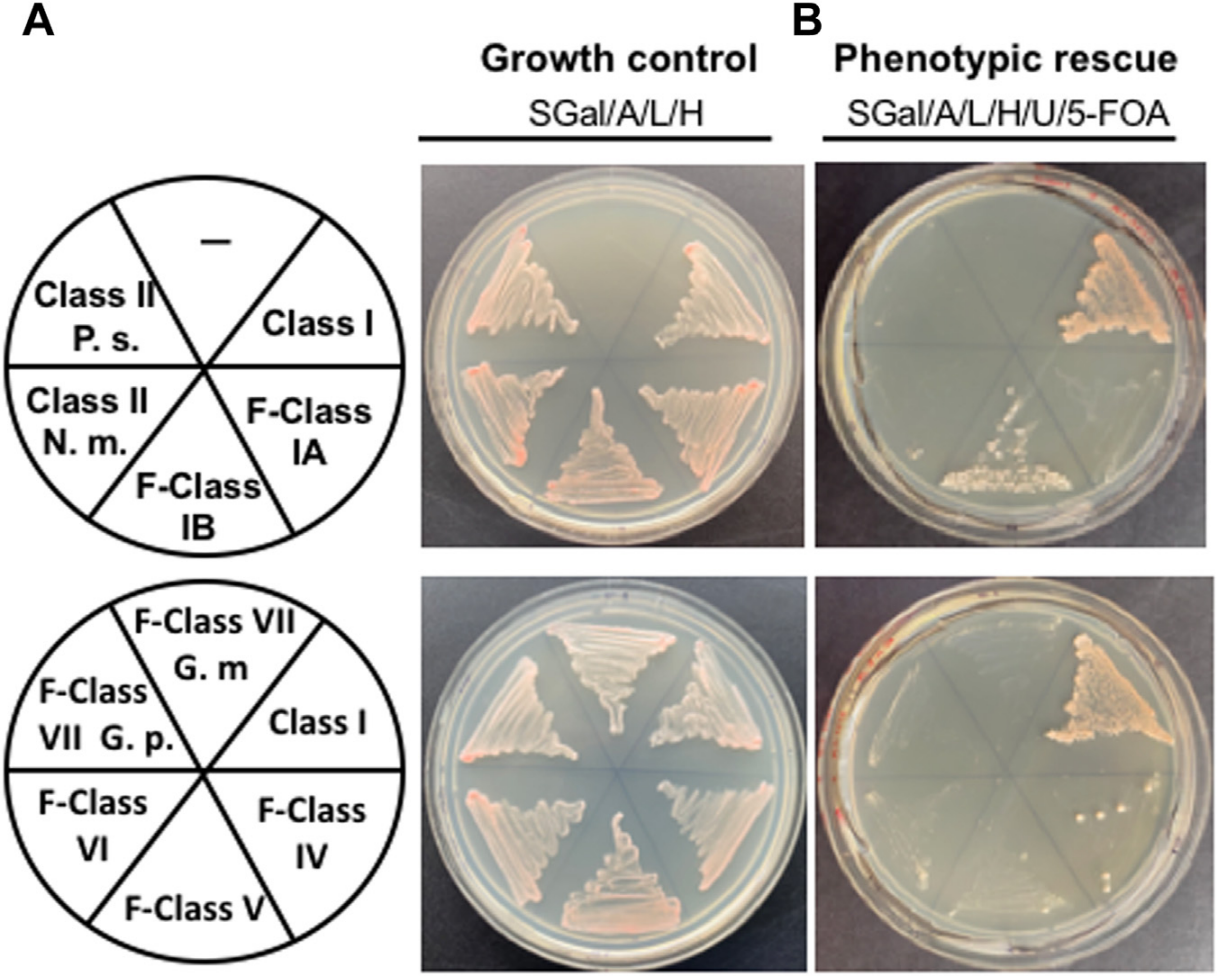
**Fungal classes of eIF4E differentially rescue the lack of *S. cerevisiae* endogenous eIF4E.** Phenotypic rescue was performed using eIF4E-HA from the Class I, *S. cerevisiae* (positive control); F-Subclass IA, *Cryptococcus gattii*; F-Subclass IB, *Mitosporidium daphnia*; Class II, *Nematocida major* (*N. m.*); Class II, *Punctularia strigosozonata* (*P. s.*); F-Class IV, *Uncinocarpus reesii*; F-Class V, *Ascochyta rabiei*; F-Class VI, *Malassezia globosa*; F-Class VII, *Gilbertella persicaria (G. p.*); and F-Class VII, *Gamisella multidivaricata (G. m.*). *A,* Growth control. *B,* Phenotypic rescue. *S. cerevisiae* eIF4E (positive control) and F-Class IB eIF4E showed a robust growth; F-Class IV eIF4E showed less but significant growth. *S*, yeast nitrogen base; —, no plasmid; *A*, adenine; *L*, leucine; *H*, histidine; *U*, uracil; *5-FOA*; 5-fuoroorotic acid.

Next, we performed phenotypic rescue assays to analyze whether representative eIF4E orthologs from all fungal classes (those analyzed in Fig. 3) can rescue the lethality due to the lack of *S. cerevisiae* eIF4E (Class I). To do so, we performed plasmid shuffling experiments using a *CDC33*-knockout strain (27) to replace *S. cerevisiae* endogenous eIF4E by fungal orthologs as the sole source of eIF4E. As shown in Fig. 4, eIF4E belonging to the F-Class IB rescued the lack of endogenous eIF4E with the highest efficiency, and F-Class IV with lower but significant efficiency too. Proteins of classes F-IA, II, F-V, F-VI, and F-VII did not rescue. A similar expression of the tested proteins was not determined.

## Discussion

### The eIF4E diversity does not fit into the current classification

We analyzed the eIF4E divergence across six *phyla* of Fungi, five of them in which eIF4E divergence has not been previously compared. Overall, we identified proteins matching Class I and Class II eIF4Es, and cognates that did not fit with the current classification corresponding to four novel clades that we termed F-Class IV to F-Class VII. Proteins from other eukaryotes, such as protists, do not match the current eIF4E classification either. Therefore, eIF4Es from Trypanosomids are classified into three groups (28, 29), namely Group 1 (eIF4E-1 and -2; Fig. S26), Group 2 (eIF4E-3 and eIF4E-4; Fig. S27) and Group 3 (eIF4E-5 and eIF4E-6; Fig. S28). eIF4Es from other protist groups, including dinoflagellates, cryptomonads, apicomplexan, ciliates, and heterokonts, were also classified into the alternative clades eIF4E-1, eIF4E-2, and eIF4E-3 divided into nine sub-clades (30, 31, 32, 33) (Fig. S29).

The impossibility of sorting out many eIF4Es from different taxa within the current classification reflects the high diversity of this protein across eukaryotes and makes evident the need for an updated categorization of this family of proteins. This prompted us to upgrade the current classification, so that it fits the eIF4E diversity, and not the other way around.

### New classification of eIF4Es

To simplify the assortment of eIF4E homologs, we propose to categorize them based both in phylogenetically coherent groups and the nature of the amino acids key for cap-binding as determined for prototypical eIF4Es, namely W56, W102, E103, R/K112, R/K157, K/R162, and W166 (human protein numbering) (5, 6, 7, 13). We did not use the conservation of W43 as a consideration in our classification, in contrast to the previous classification strategy (9). Thus, human eIF4E-1 remains the prototypical Class I protein, with orthologs in Metazoan, land plants, Fungi, and protists, and should be the reference for further nomenclature. Class II, present in the same kingdoms, was also defined by Joshi *et al.* (2005) as proteins containing the conservative change W56 ➜ Y/F. Here we add to this definition the presence of the non-conservative substitutions R/K162 ➜ I/V/L (Fig. 2 and Fig. S8).

We defined a new class of eIF4Es when non-conservative changes in the amino acids key for cap-binding appeared in a sequence. Additional classes, grouping proteins specific to each lineage were identified with a capital letter according to that group. Thus, Metazoan-specific Class III (9) here was renamed M-Class III. Fungal-specific classes were termed F-Class IV to F-Class VII, as well as F-Subclasses. Trypanosomids (28, 29) Group 1 eIF4Es conserve all residues key for cap-recognition and share a similar size to prototypical eIF4Es. Thus, they were included in Class I proteins. No proteins of Class II have been reported in Trypanosomids (20). Group 2 and Group 3 eIF4Es show non-conservative changes in residues involved in cap recognition and contain insertions of amino acid stretches, as well as amino- and or carboxy-terminal extensions. We renamed these proteins T-Class III and T-Class IV eIF4Es, respectively. Other protists such as Dinoflagellates (30, 31, 32, 33) also possess prototypical Class I eIF4E proteins. Additionally, protists with two flagella of unequal length (heterokonts) contain a Class II-like protein that shows the conservative changes W56 ➜ Y and R/K112 ➜ H. A third class contains the change W56 ➜ Y, the non-conservative substitutions R/K112 ➜ C and K/R162 ➜ V/A, and two amino acid stretch insertions. Fig. S29 shows a summary of Trypanosomids and Dinoflagellates eIF4E under the new classification. It is worth mentioning that E103 and W166 are the unique non-variable residues in all eukaryotes studied so far studied.

### Functional diversification of fungal eIF4Es

We showed that Class I, F-Class IB, and F-Class IV eIF4Es (in this order of efficiency) rescued the lethality of the *CDC33* gene deletion in *S. cerevisiae*. These results agree with the fact that F-Class protein IB is the most similar to *S. cerevisiae* eIF4E (a Class I protein). Whereas F-Class IB proteins possess the conservative changes W56 to F (human protein nomenclature), F-Class IV peptides possess the substitutions W56 to Y, R/K112 to C, and KR162 to L. Interestingly, both F-Class IB and F-Class IV peptides are the only eIF4E families that do not contain amino acid extensions, which makes them the most similar in both size and global three-dimensional shape to the *S. cerevisiae* protein among all fungal paralogs.

The structural variability among proteins observed in the cap-binding pocket and the global tertiary structure, suggests different biochemical capabilities during mRNA recruitment. This, together with the uneven ability to cross-complement *S. cerevisiae* eIF4E by the fungal counterparts, shows a functional diversification, as it has been also shown for plant and animal species possessing several eIF4E cognates (1, 28, 34, 35, 36). Moreover, the high conservation of mammalian S209 among metazoans and fungi supports the notion that its phosphorylation also may be key in fungi.

## Experimental procedures

### Sequence obtaining, alignment, and cladistic analysis

To obtain the sequences of eIF4E homologous proteins, we downloaded the 538 fungal genomes available from NCBI’s RefSeq (18) by the end of September 2023. Using the diamond alignment tool (37), we compared the sequence of the *S. cerevisiae* eIF4E protein (NP_014502.1) against all the proteins annotated in all RefSeq’s fungal genomes and obtained a total of 1401 homologs. To complement this protein set, we queried all the fungal proteins against the PFAM database (38) using mmseqs2 (39). The PFAM entry, PF01652, matched all 1401 proteins found using diamond, plus an additional 61 proteins for a total of 1462.

Sequences were aligned using the Clustal Omega program (https://www.ebi.ac.uk/Tools/msa/clustalo/) and optimized by eye. The following conservative amino acids were considered: G and A; S and T; K, R and H; E, D, Q and N; and L, I, M, V, C, Y, F, and W. Cladistic analyses were conducted with the MEGA7 program (40) using the Neighbor-Joining method (41). Cladograms were drawn to scale, with branch lengths in the same units as those of the evolutionary distances used to infer the phylogenetic tree. The evolutionary distances were computed using the Poisson correction method and are in the units of the number of amino acid substitutions per site.

### Three-dimensional structure prediction

All structures were predicted by Alphafold2 (42) and were aligned with Worldwide Protein Data Bank (PDB) structure 6FC1 (11) which depicts *S. cerevisiae* eIF4E in complex with the m^7^GTP cap. Images were visualized using PyMOL software (Schrödinger, L., & DeLano, W. (2020). *PyMOL*. Retrieved from http://www.pymol.org/pymol).

### Plasmids

eIF4E cDNAs were obtained from GeneScript. eIF4E sequences from different fungal species (Table S4) were recoded toward the *S. cerevisiae* preferential use of codons, and cloned into the multicopy plasmid pESC-TRP containing an in-frame hemagglutinin-tag (HA-tag) to obtain pESC-TRP-eIF4E-HA constructs that express eIF4E-HA cDNAs under the regulable Gal10 promoter.

### Functional complementation of eIF4E in *S. cerevisiae*

Phenotypic rescue assays were performed according to (27), consisting of the replacement of the *S. cerevisiae* essential gene *CDC33* (encoding eIF4E) by the *eIF4E* cDNA from other fungal species. We used the haploid strain RH2585 *ΔCDC33::KanX*, <pVTU-eIF4E>, a generous gift of Michael Altmann, Universität Bern, Switzerland (27). The strain RH2585 was transformed with the pESC-TRP-eIF4E-HA constructs and independent colonies were transferred onto S, agar, adenine, leucine, histidine, and galactose (to switch on the Gal10 promoter; SGal/A/L/H) plates. To test for phenotypic rescue, colonies were further streaked on the same medium supplemented with uracil and fluoroorotic acid (5-FOA; SGal/A/L/H/U/5-FOA) to trigger the removal of plasmid < pVT-URA3-eIF4E> and grown for 6 days.

## Data availability

All data are included in this publication.

## Supporting information

This article contains supporting information (5, 6, 7, 9, 11, 12, 13, 14, 15, 17, 18, 21, 22, 23, 24, 25, 26, 28, 29, 30, 32, 37, 38, 39, 40, 41, 42, 43, 44, 45, 46, 47, 48, 49, 50, 51, 52).

## Conflict of interest

The authors declare that they have no conflicts of interest with the contents of this article.
