# Supplementary material for: Analysis of eIF4E-family members in fungi contributes to their classification in eukaryotes
Source: J Biol Chem. 2024 Dec 21;301(2):108129. doi: 10.1016/j.jbc.2024.108129 (PMC11791286; doi:10.1016/j.jbc.2024.108129)
Supplement: Supplementary Figures Tables [file mmc2.docx]

**Supporting information for:**

**Analysis of eIF4E-family members in Fungi contributes to their classification in eukaryotes**

Greco Hernández^1, 2^*, Daniela Ross-Kaschitza^3^, Gabriel Moreno-Hagelsieb^4^, Alejandra García^1^,Dora Emma Vélez^1^, Blanca Licia Torres^1^

^1^ mRNA and Cancer Laboratory, Unit of Biomedical Research on Cancer, National Institute of Cancer (INCan), Mexico City 14080, Mexico.

^2^ Escuela de Medicina y Ciencias de la Salud, Tecnológico de Monterrey. Mexico City 14380. Mexico.

^3^ Institut für Biochemie und Molekulare Medizin (IBMM), University of Bern, 3012 Bern, Switzerland.

^4^ Department of Biology, Wilfrid Laurier University. 75 University Ave. W. Waterloo, ON N2L 3C5, Canada.

* To whom correspondence should be addressed: Greco Hernández.

Email: [ghernandezr@incan.edu.mx](mailto:ghernandezr@incan.edu.mx); greco.hernandez@tec.mx.

Phone: +52/55/36935200 ext. 246.

**This file includes:**

Supplementary text.

Supplementary Figures S1 to S29.

Supplementary Tables S2 to S4.

Supplementary References.

**Experimental procedures**

*Sequence obtaining, alignment, and cladistic analysis*

To obtain the sequences of eIF4E homolog proteins, we downloaded the 538 fungal genomes available from NCBI’s RefSeq (1) by the end of September 2023. Using the diamond alignment tool (2), we compared the sequence of the *Saccharomyces cerevisiae* eIF4E protein (NP_014502.1) against all the proteins annotated in all RefSeq’s fungal genomes and obtained a total of 1401 homologs. To complement this protein set, we queried all the fungal proteins against the PFAM database (3) using mmseqs2 (4). The PFAM entry, PF01652, matched all 1401 proteins found using diamond, plus an additional 61 proteins for a total of 1462.

Sequences were aligned using the Clustal Omega program (<https://www.ebi.ac.uk/Tools/msa/clustalo/>) and optimized by eye. The following conservative amino acids were considered: G and A; S and T; K, R and H; E, D, Q and N; and L, I, M, V, C, Y, F, and W. For demonstrated phosphorylation of S residues, the phospho-mimicking substitutions E and D were considered conservative changes. Cladistic analyses were conducted with the MEGA7 program (5) using the Neighbor-Joining method (6). Cladograms were drawn to scale, with branch lengths in the same units as those of the evolutionary distances used to infer the phylogenetic tree. The evolutionary distances were computed using the Poisson correction method and are in the units of the number of amino acid substitutions per site.

*Three-dimensional structure prediction*

All structures were predicted by Alphafold2 (7) and were aligned with Worldwide Protein Data Bank (PDB) *S. cerevisiae* eIF4E structure 6FC1 (8) in complex with the m^7^GTP cap. Images were visualized using PyMOL software (Schrödinger, L., & DeLano, W. (2020). *PyMOL*. Retrieved from <http://www.pymol.org/pymol>).


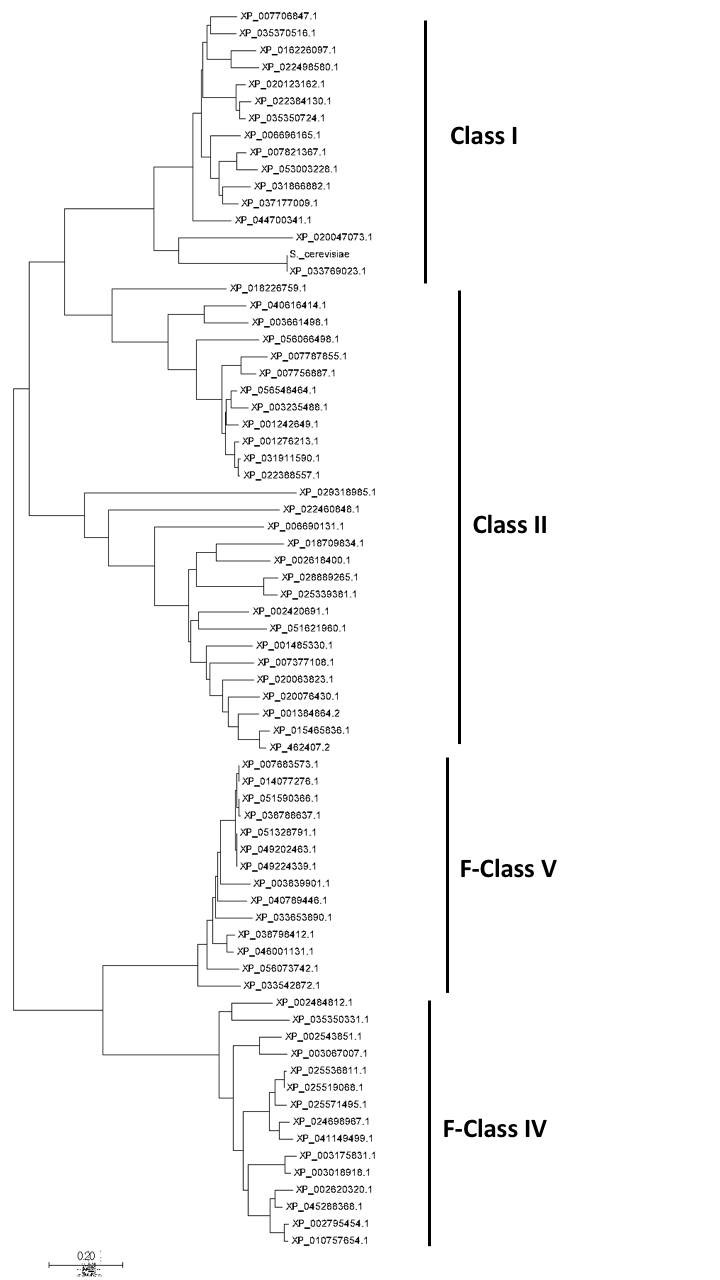


**Supplementary Fig. S1. Cladogram showing the relationships of *Ascomycota* eIF4E orthologs.** A neighbor-joining phylogeny of selected sequences of full-length proteins is presented. The Fungi-specific classes are indicated by a F- prefix.

**Supplementary Fig. S2. Cladogram showing the relationships of *Basidiomycota* eIF4E orthologs.** A neighbor-joining phylogeny of selected sequences of full-length proteins is presented. The Fungi-specific classes and subclasses are indicated by a F- prefix.

**
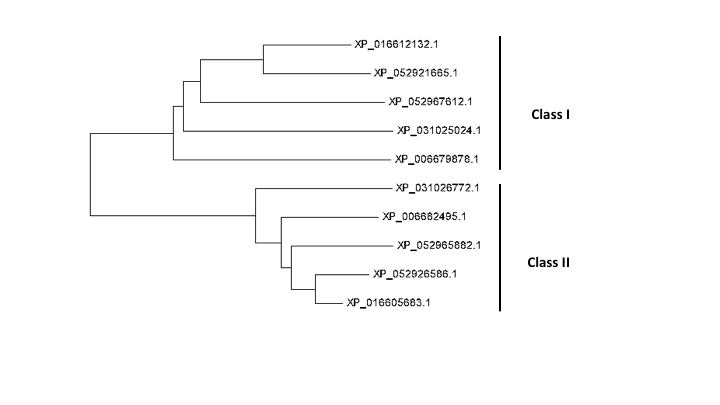
**

**Supplementary Fig. S3. Cladogram showing the relationships of *Chytridiomycota* eIF4E orthologs.** A neighbor-joining phylogeny of selected sequences of full-length proteins is presented.

**
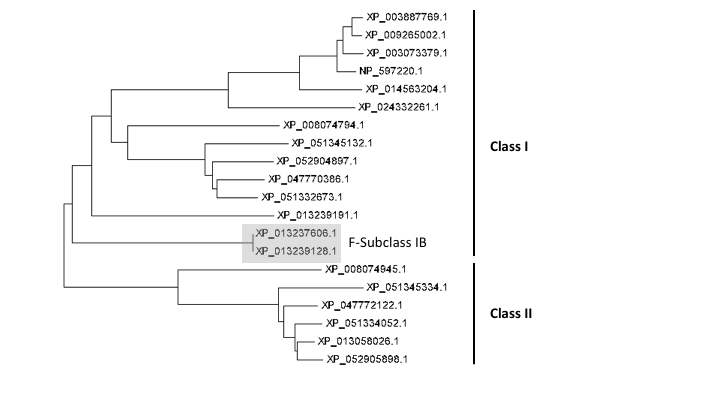
**

**Supplementary Fig. S4. Cladogram showing the relationships of *Rozellomycota* eIF4E orthologs.** A neighbor-joining phylogeny of selected sequences of full-length proteins is presented. The Fungi-specific subclass IB is shaded in gray.


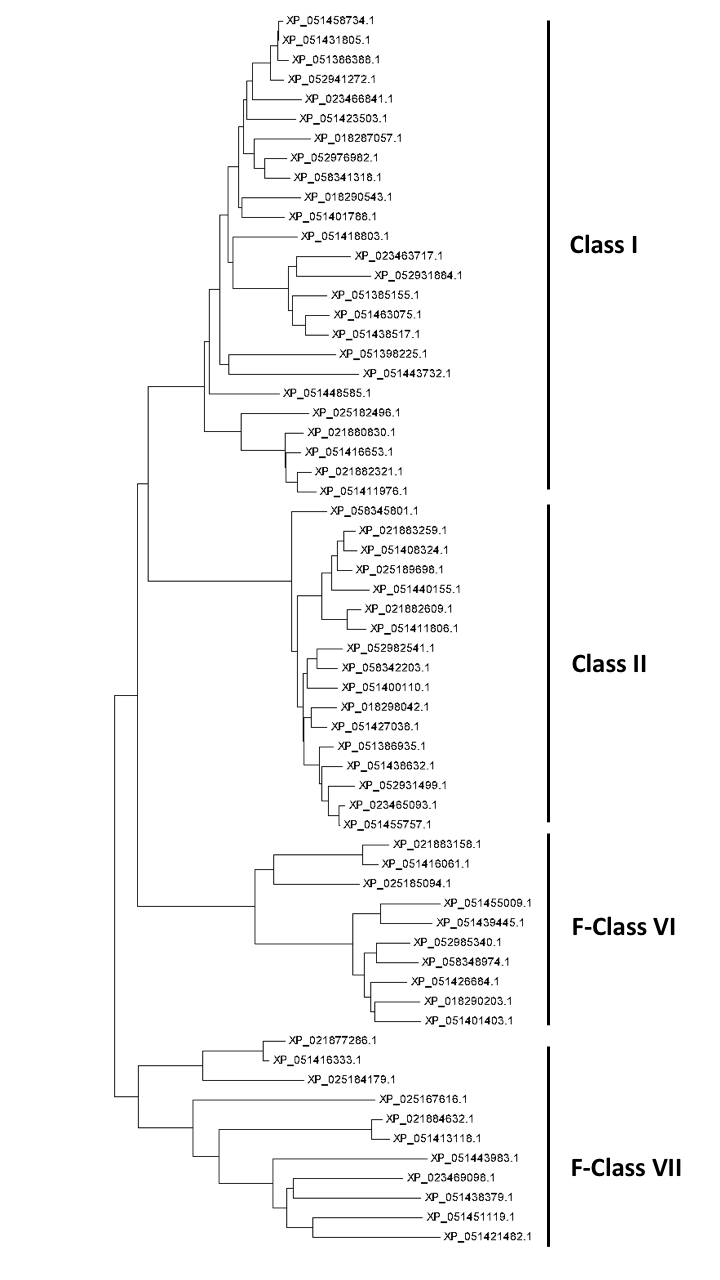


**Supplementary Fig. S5. Cladogram showing the relationships of *Mucoromycota* eIF4E orthologs.** A neighbor-joining phylogeny of selected sequences of full-length proteins is presented. The Fungi-specific classes are indicated with a F- prefix.

**
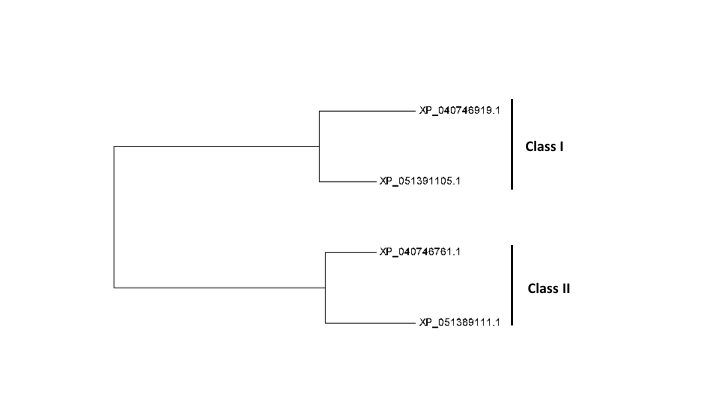
**

**Supplementary Fig. S6. Cladogram showing the relationships of *Zoopagomycota* eIF4E orthologs.** A neighbor-joining phylogeny of selected sequences of full-length eIF4Es is presented

Human eIF4E -----------------------------------------MAT-VEPETTPTPN--PP- 15

Rabbit eIF4E -----------------------------------------MAT-VEPETTPTPN--PP- 15

Drosophila eIF4E-1 MQSDFHRMKNFANPKSMFKTSAPSTEQGRPEPPTSAAAPAEAKD-VKPKEDPQETGEPAG 59

Wheat p26 -----------------------------------------MAEDTETRPA--SAGAE-E 16

Yeast eIF4E (As) -----------------MSV---------------------------------EEVSK-K 9

NP_594228.1 Spo(As) MQ----------------------TEQ----PPKESQ----------------------- 11

NP_595451.1 Spo(As) MADAEDSRHSKNEGFPNTSLITEKLDLLDLFGSPKVKTEREGRPARLLEGLSAVNAETA- 59

XP_003887769.1 Eh (Ro) ------------------------------------------------------------ 0

XP_024332261.1 Vc (Ro) ------------------------------------------------------------ 0

XP_041300350.1 Sd (Ba) -----------------MAALPASFQHANKAAI-------AAAL-AENSESIGADVEP-G 34

XP_037222000.1 Mi (Ba) -----------------MSVLSG------PAPS-------GAALSDPPKDATIDDSEP-G 29

XP_016612132.1 Sp (Ch) ----------MATTAASLSATDDSFQNGAPHPS-------NHNP-IAESDT--PRTSS-P 39

XP_031025024.1 Sm (Ch) -----------------------------------------MAP-AE----------E-K 7

XP_051458734.1 Mm (Mu) -----------------MT------------------------E-QETRPI--DVAEQ-E 15

XP_051386388.1 Cr (Mu) -----------------MT------------------------E-E-TRPT---VEEK-E 13

XP_040746919.1 Lp (Zo) -----------------MT------------------------E---------------V 4

XP_051391105.1 Ka (Zo) -----------------MT------------------------D---------------V 4

*

Human eIF4E TTEEEKTESNQE------VANPEHYIKHPLQNRWALWFFKNDK----------------- 52

Rabbit eIF4E PAEEEKTESNQE------VANPEHYIKHPLQNRWALWFFKNDK----------------- 52

Drosophila eIF4E-1 NTATTTAPAGDD------AVRTEHLYKHPLMNVWTLWYLENDR----------------- 96

Wheat p26 REEGEIADDGD----GSSAAAAGRITAHPLENAWTFWFDNPQGKSR-------------- 58

Yeast eIF4E (As) FEENVSVDDTTATPKTVLSDSAHFDVKHPLNTKWTLWYTKPAVD-K-------------- 54

NP_594228.1 Spo(As) TENTVSEP-QEKALRTVFDDKINFNLKHPLARPWTLWFLMPPTP---------------- 54

NP_595451.1 Spo(As) -----------------------FVKTHPLQHEWTLWFLKPPTQ---------------- 80

XP_003887769.1 Eh (Ro) ---------------------------MNLASQWVLWQNNNDE-SN-------------- 18

XP_024332261.1 Vc (Ro) ---------------------------MKLQSKWVIWQNTSED-SD-------------- 18

XP_041300350.1 Sd (Ba) EIQEVDMQAQADDIRTVFSHPTSFNVKHPLYSPWTLWFDSPATKGRNLPQTPISSFPQTP 94

XP_037222000.1 Mi (Ba) EVQEVDMQTQAETIRTVFSDPTNFNVKHPLYSPWTLWFDSPQTKGRNMPQTPISAFPQTP 89

XP_016612132.1 Sp (Ch) EPDQVS-DDESSSFKTIFDDPKNFNAKHPLQNRWTMWFDNPGKRTN-------------- 84

XP_031025024.1 Sm (Ch) TAET-TTESPATDIITVFDDPINFTHKHPLQHKWCVWFDSAQGKQN-------------- 52

XP_051458734.1 Mm (Mu) PIVE-QGNNDDNSVRTVFHDAKNYNVKHPLQNTWTLWFDNPGKKAN-------------- 60

XP_051386388.1 Cr (Mu) IESP-SEQQNDESLKTVFHDPKNYNVKHPLQNTWTLWFDNPGKKAN-------------- 58

XP_040746919.1 Lp (Zo) Q-N--T-----AAAKTVLTDAENFDTVHPLNSEWTLWFDNPSRRTN-------------- 42

XP_051391105.1 Ka (Zo) QAAGSSDNI-TQEVKTVLNDAKDFNALHGLNSEWTLWFDNPSKRTS-------------- 49

*

** G**

Human eIF4E ---------SKTWQANLRLISKFDTVEDFWALYNHIQLSSNLMPGCDYSLFKDGIEPMWE 103

Rabbit eIF4E ---------SKTWQANLRLISKFDTVEDFWALYNHIQLSSNLMPGCDYSLFKDGIEPMWE 103

Drosophila eIF4E-1 ---------SKSWEDMQNEITSFDTVEDFWSLYNHIKPPSEIKLGSDYSLFKKNIRPMWE 147

Wheat p26 ---------QVAWGSTIHPIHTFSTVEDFWGLYNNIHNPSKLNVGADFHCFKNKIEPKWE 109

Yeast eIF4E (As) ---------SESWSDLLRPVTSFQTVEEFWAIIQNIPEPHELPLKSDYHVFRNDVRPEWE 105

NP_594228.1 Spo(As) ---------GLEWNELQKNIITFNSVEEFWGIHNNINPASSLPIKSDYSFFREGVRPEWE 105

NP_595451.1 Spo(As) ---------GLEWSDLLKEIISFKTVEEFWGIFKTISKASMLPAKSDYSYFLKGIRPEWE 131

XP_003887769.1 Eh (Ro) ---------AKSWGDDLVAVGEVSTVPEFLYLCDEISN-VGIGKLCTMNLFRKGVKPMWE 68

XP_024332261.1 Vc (Ro) ---------VKSWADDLKNVGEFTQIEEFKFFADELKE-KKLDKLLSLKIFKSGIKPMWE 68

XP_041300350.1 Sd (Ba) VPQTPSAAAAQGWMEDIKRVVSFDSVEEFWGLYNHIVPPSQLPQKANYYLFKEGIIPAWE 154

XP_037222000.1 Mi (Ba) VAQTPGVAAAQGWMEDIKRVISFDSVEEFWGLYNNIVPPSQLPQKANYYLFKEGIIPAWE 149

XP_016612132.1 Sp (Ch) ---------QHNWSNNLKNLITVDTVEDFWGVYNNVVKASQLTHGSNYHIFKEGVQPMWE 135

XP_031025024.1 Sm (Ch) ---------QKNWMDNLKNLVTFDSVEDFWGIMNHIMKASALPAGSNYHVFKEGIQPMWE 103

XP_051458734.1 Mm (Mu) ---------AASWSQNLKEIVNVDTVEDFWGVHNNIVKVNHLEISSNYHVFKKGIRPEWE 111

XP_051386388.1 Cr (Mu) ---------AASWSQNLKEIVDVNTVEDFWGVHNNIVKVNHLEISSNYHVFKKGIRPEWE 109

XP_040746919.1 Lp (Zo) ---------TSSWTQNLKEIVTLGTVEDFWGVHSNVTKAVDLPNGSNYHLFRKGIKPMWE 93

XP_051391105.1 Ka (Zo) ---------TSNWTANLKEIVTLKAVEDFWGVYNNVAIASDLPNGSNYHLFRKGVRPMWE 100

**P**

Human eIF4E DEKNKRGGRWLITLNKQQRRSDLDRFWLETLLCLIGESFDDY-----------SDDVCGA 152

Rabbit eIF4E DEKNKRGGRWLITLNKQQRRSDLDRFWLETLLCLIGESFDDY-----------SDDVCGA 152

Drosophila eIF4E-1 DAANKQGGRWVITLNKSS-KTDLDNLWLDVLLCLIGEAFDH------------SDQICGA 194

Wheat p26 DPICANGGKWTISCGRG----KSDTFWLHTLLAMIGEQFDFG------------DEICGA 153

Yeast eIF4E (As) DEANAKGGKWSFQLRGK--GADIDELWLRTLLAVIGETIDED-----------DSQINGV 152

NP_594228.1 Spo(As) DVHNKTGGKWAFQNKGRGGNA-LDEMWLTTVLAAIGETLDP--T---------GQEVMGV 153

NP_595451.1 Spo(As) DPQNMNGGKWAYQSKHKGS--NLDELWLYMVLAAIGETLDP--T---------GKEVTGV 178

XP_003887769.1 Eh (Ro) DEANIDGGRIIMDVPVVG-RDNVGDLWKKTMAFCVSNSV---------------DNVCGC 112

XP_024332261.1 Vc (Ro) DPRNMNGGRLVIDIPTAS-GYNPEEVFLLTVAFCISNTA---------------AGICGC 112

XP_041300350.1 Sd (Ba) DEANKNGGKWSIQLPKEKNRSQVDKMWLYTMLAAIGETFDPHLTSGEATEPNTQSLITGV 214

XP_037222000.1 Mi (Ba) DEANKNGGKWSIQLPKDKNRGNVDKMWLYTMLAAIGETFDPSLTSADPAGSPPSSLITGV 209

XP_016612132.1 Sp (Ch) DPHNANGGKWVVQLPKSKR-SELDQMWLFSVLAAIGETFPDS------------DEICGI 182

XP_031025024.1 Sm (Ch) DPQNKKGGKWVVSITKKHR-GQMDTWWLNTLMGMIGEAFEDG------------AEIMGA 150

XP_051458734.1 Mm (Mu) DAANANGGKFSIQFPRNRTGESINDYWLNLILAMLGEQFQYE------------DEICGA 159

XP_051386388.1 Cr (Mu) DPANANGGKFSIQFPRNRTGEAINDYWLNLILTMLGEQFKYE------------DEICGA 157

XP_040746919.1 Lp (Zo) DAANANGGRWGIQLQRS-VGGKANDLWLNTLLACIGETFDAS------------TDVCGA 140

XP_051391105.1 Ka (Zo) DAANARGGKWGYQFQRS-IGEKVNEHWLHTLLACIGESFDSS------------PDVCGA 147

**P P m**

Human eIF4E VVNVRAKGDKIAIWTTECE-------NREAVTHIGRVYKE-RLGLPP--------KIVIG 196

Rabbit eIF4E VVNVRAKGDKIAIWTTECE-------NRDAVTHIGRVYKE-RLGLPP--------KIVIG 196

Drosophila eIF4E-1 VINIRGKSNKISIWTADGN-------NEEAALEIGHKLRD-ALRLGR--------NNSLQ 238

Wheat p26 VVSVRQKQERVAIWTKNAA-------NEAAQISIGKQWKE-FLDYKD----------SIG 195

Yeast eIF4E VLSIRKGGNKFALWTKSED--------KEPLLRIGGKFKQ-VLKLTD--------DGHLE 195

NP_594228.1 Spo(As) VINMRKGFYRLAVWTKSCN-------NREVLMEIGTRFKQ-VLNLPR--------SETIE 197

NP_595451.1 Spo(As) VCNMRKGFYRIAVWTRNCN-------DKDVLEKIGLRFKE-VLGISD--------KETIE 222

XP_003887769.1 Eh (Ro) VLSEKQSFYKLAIWFGKDY----------NQDTIKEMWQE-ALGGGN---------LSVY 152

XP_024332261.1 Vc (Ro) VVMSKHEFIKISLWIENEQ----------YHDDIMAKWKD-VLVRYD---------LNIY 152

XP_041300350.1 Sd (Ba) IVSTRPQFYRLSIWTRLAPSGDE-DKLRERIETVGRHFKINVLGYPETQKLAGPLATEVE 273

XP_037222000.1 Mi (Ba) IVSTRPQFYRLSIWTRLAPSGSEDDKLRERIEGVGKHFKTSVLGYADHAKLAGPLSTEVE 269

XP_016612132.1 Sp (Ch) VVSIRKQQDRLSLWTKSAL-------DEDKCRASGQHWKG-VLGLGD--------GEKIG 226

XP_031025024.1 Sm (Ch) VVSARRSYDRISLWTKTGA-------IQDVQERIGKQFKQ-IMGVDS--------DITLG 194

XP_051458734.1 Mm (Mu) VVSVRKVFYRVALWIKSSE-------KNEKIETIGRQLKE-FLNLNN--------TLVVE 203

XP_051386388.1 Cr (Mu) VVSVRRVFYRVALWIKSSE-------KNETTETIGRQLKE-FLNLNS--------SLVVE 201

XP_040746919.1 Lp (Zo) VFSNRKQCFRIAIWTRNAA-------DKEACEAIGRHLKT-VLNGEH----------QLE 182

XP_051391105.1 Ka (Zo) VFSNRKSCFRIAIWTRNAD-------DQEACENIGRHFKS-VLGVSH----------PLE 189

Human eIF4E YQSHADTATKS-G--ST-TKNRFVV 217

Rabbit eIF4E YQSHADTATKS-G--ST-TKNRFVV 217

Drosophila eIF4E-1 YQLHKDTMVKQ-G--SN-VKSIYTL 259

Wheat p26 FIVHEDAKRSDKG-----PKNRYTV 215

Yeast eIF4E (As) FFPHSSANGRHPQ-----P-SI-TL 213

NP_594228.1 Spo(As) FSAHEDSSKS--G--STRAKTRMSV 218

NP_595451.1 Spo(As) YSAHEDSSKA--G--SMRAKTRMSL 243

XP_003887769.1 Eh (Ro) SFLHKKSLDSNKGKKKWGGRR---- 173

XP_024332261.1 Vc (Ro) FLLHKKGIDGNKGRKTW-NKKKAYF 176

XP_041300350.1 Sd (Ba) FLSHKDSEKK--GK----AAKKLVI 292

XP_037222000.1 Mi (Ba) FLSHKDSEKKG-GK-S----RKIVV 288

XP_016612132.1 Sp (Ch) YQAHSDALRKNS---SFSNQDMYTV 248

XP_031025024.1 Sm (Ch) YQMHDTALKTNS---SYGNDDIYQV 216

XP_051458734.1 Mm (Mu) FTPHGDSAAKSS-------ENRFTI 221

XP_051386388.1 Cr (Mu) FTPHGDSAAK-----S--GENKFTI 219

XP_040746919.1 Lp (Zo) YMPHTDNAK------S-GPKVLYSV 200

XP_051391105.1 Ka (Zo) YFAHSDSSK------T-GNSVAYTV 207

**Supplementary Fig. S7.** **Sequence comparison of prototypical Class I eIF4Es with orthologs belonging to six *phyla* of fungi.** Full-length proteins were compared.Residues involved in the cap recognition (9-12) are indicated as follows: , W binding the guanine by  –  interactions; *G*, residue recognizing the guanine ring; *P*, residues interacting with the phosphate groups; *m*, W recognizing the cap methyl group. *Asterisks* indicate W43 and W56 of the human protein used to classify the eIF4E-family members into three classes (13). Residues identical to human eIF4E are shaded in black boxes. Conservative changes are in grey boxes. Purple boxes highlight conservation of the phosphorylated residues S2, S15, and S28 of *S. cerevisiae* eIF4E (14), and S209 or S251 of human/mouse eIF4E (15,16) and *Drosophila* eIF4E-1 (17), respectively. Phosphorylation of *S. cerevisiae* S28 was demonstrated to increase eIF4G affinity (18). Phospho-mimicking E or D residues in the phosphorylatable positions (18,19) are also highlighted in gray boxes. Gaps are represented by dashes. Human eIF4E (acc. numb.M15353) (20); Rabitt (*Oryctolagus cuniculus*) eIF4E-1 (acc. numb.X61939) (21); *Drosophila melanogaster* eIF4E-1 (acc. numb.CG4035) (22,23); Wheat (*Triticum aestivum*) eIF4E p26 (acc. numb.Z12616) (24); Fungal species: yeast *Saccharomyces cerevisiae* (acc. numb. NP_014502.1) (25,26); Spo, *Schizosaccharomyces pombe* (27,28); Eh, *Encephalitozoon hellem*; Vc, *Varimorpha ceranae*; Sd, *Suillus discolor*; Mi, *Mycena indigotica*; Sp, *Spizellomyces punctatus*; Sm, *Synchytrium microbalum*; Mm, *Mucor mucedo*; Cr, *Cokeromyces recurvatus*; Lp, *Linderina pennispora*; Ka, Kickxella alabastrina. *Phylum* name is indicated in parenthesis: (As)*, Ascomycota*; (Ro), *Rozellomycota*; (Ba), Basidiomycota; (Ch), *Chytridiomycota*; (Zo), Zoopagomycota; (Mu), Mucoromycota.

*

* ****

Human 4EHP EHPLQYNYTFWYS-RRTPGRPTSSQSYEQNIKQIGTFASVEQFWRFYSHMVRPGDLTGHSDFH 114

Drosophila 4EHP ENRLQHTYCLWFSRKE-TQRAA--ADYSKSLHMVGRCASVQQWWSLYSHLIRPTALKPYRELL 104

C.elegans IFE-4 DHQLQYSYTFSYF-MRPTG-KFDPEDYASYVQPVGIMKSVEQFWSIMVHFKRPTEMCDKADIH 90

Arabidopsis nCBP LHPLRYKFSIWYT-RRTPGVRNQ--SYEDNIKKMVEFSTVEGFWACYCHLARSSLLPSPTDLH 101

XP_008074945.1 Vcf (Ro) THPLFVPFTVKTI-T-RNTTKKEPFDFCNSLKKLCTLKSVENLLYFLNH-VNFDHIEGITDIS 61

XP_047772122.1 Nm (Ro) PSTLSSALVLSSV-FRSVNIKGDSEDFKSKIKQEAKITTPEEFLYVIRRLLKLQDIKTITDLS 63

XP_052905898.1 Na (Ro) SQKLASTLILSSV-FRCMTVKGDTEDFKSKIKQEAIIETKEEFLYLIRRMQKLQEIKPITDLS 64

XP_013058026.1 Np (Ro) NQKLASTLILSSV-FRCLTVKGDTEDFKSKIKQEATVETQEEFLYLIRRLQKLQDIKPITDLS 64

XP_019014281.1 Kp (Ba) RHPLRQDWSISYV-HRPPGAKV---EYEKEIRKVATFGSIESFLHLYSHITPPNELPPVTDIL 96

XP_047804977.1 Pst(Ba) QHKLRSKWVFWLL-HRPPSKKISEEEYGKAMRRLGSCDTVEQFFSLYLHIKRPSQHLPISDLH 236

XP_009549304.1 Hi (Ba) VHPLRNTWVFWFRQQRAPGNKIT--NYEEGIKKISAFHSVESFWSLWTHLHPPSALLPTTDYL 133

XP_007382918.1 Ps (Ba) VHPLKNTWVFWFRQQRAPGNKTL--NYEEGIKKVAAFSSVESFWSLWTHVNPPSALQPTTDYL 134

XP_031026772.1 Sm (Ch) SSQRHIIVYTRFM-HRPPGQKIISDDYLSGVKEVGTFATIEEFWGLYSRMRRPNELPNISDIH 117

XP_052926586.1 Fj (Ch) THPLRYPWVFWFM-HREAGAKIE--NYNNSIKKISTFSTVEEFWGVYNRLTRPMELNNVCDFH 79

XP_006682495.1 Bd (Ch) FHALHYSWVFWFM-HRSPGAKIQ--DYTNEIKHVCTFSTVEEFWGAFSHMKRPGELSNISDYH 91

XP_040746761.1 Lp (Zo) ------------M-HRAPGEKIT--DYESAMIRLASFGTVEGFWGVYSHLLRPNQVPTITDYH 48

XP_051389111.1 Ka (Zo) EHPLEFAWTFWFM-HRPPGQKID--DYEAAMIRIATFASVESFWAVYSHIKRPDKVPTITDYH 64

XP_025189698.1 Ri (Mu) IHPLHFTWVFWFM-HRNPGSKIL--NYESSMKKIAAFSSIEDFWAVYSHLRRPHELPNISDYH 98

XP_051408324.1 Gm (Mu) RHPLHFNWVFWFM-HRAPGSKIL--NYEGAMKKIATFGSAEDFWAVYSHLKRPHELPTVSDYH 199

XP_021883259.1 Lt (Mu) LHPLHFNWVFWFM-HRAPGSKIV--NYESSMKKIATFGSVEDFWAVYSHLKRPHELPTVSDYH 218

XP_051411806.1 Gm (Mu) IHPLQFNWVFWFM-HRAPGSKIL--NYESSMKRIAGFGSVEAFWGIYSHLRRPHELPHVSDYH 80

XP_021882609.1 Lt (Mu) THPLHYNWVFWFM-HRAPGSKIL--NYESSMKKITTFGSVEAFWAVYSHLRRPNELPHVSDYH 245

**G**  **P**

Human 4EHP LFKEGI-KP-MWEDDANKNGGKWIIRLRKG----LASRCWENLILAMLGEQFMV------ 162

Drosophila 4EHP LFKQGI-IP-MWEDPANSKGGQWLIRLRKN----KVDRAWENVCMAMLGEQFLV------ 152

C.elegans IFE-4 FFKTGV-KP-VWEDPANCKGGKWIIRLKKG----LSTRIWENLLMAIIGEQFLV------ 138

Arabidopsis nCBP FFKDGI-RP-LWEDGANCNGGKWIIRFSKV----VSARFWEDLLLALVGDQLDD------ 149

XP_008074945.1 Vcf (Ro) IFKDGI-EP-LWEDKSNIKGGKWIIKLRRE---V-STRLFQKLLIRMVRQPFDK------ 109

XP_047772122.1 Nm (Ro) LFKEGI-EP-MWEDPSNLKGGKWIVKIKRN---TAEQRLFESVFLWMALVPFKT------ 112

XP_052905898.1 Na (Ro) LFKSGI-EP-MWEDPCNINGGKWIIKIKKN---TAEQRLFESLFVWMALVPFAS------ 113

XP_013058026.1 Np (Ro) LFKKGI-EP-MWEDPSNLNGGKWIIKIKKN---TAEQRLFESLFIWMALVPFST------ 113

XP_019014281.1 Kp (Ba) VFVSRIGRPGVWEE--MRDGGKFTIRLVHP----ITPLLFENLLLALIGDQFDE------ 144

XP_047804977.1 Pst(Ba) MFVDPI-KP-AWEDPENVGGGKWTIRLKKG----LANRLWETLILSLVGGGLEKLIRNNN 290

XP_009549304.1 Hi (Ba) LFHSGVRRP-VWEDPLNLPGGKWILRLRKG----VADRVWEDLVLAIVGDQFADCAAPEE 188

XP_007382918.1 Ps (Ba) LFHSGVRRP-VWEDPVNISGGKWILRLKKG----VADRIWEDLVLAIIGDQFADADS--- 186

XP_031026772.1 Sm (Ch) LFRKGT-RP-VWED--NPKGGKWIVRLKKG----LSSRYWENLVIAVIGDNFGD------ 163

XP_052926586.1 Fj (Ch) LFKQGI-RP-IWED--NLNGGKWIVRLKKG----LASRYWESLVMAMIGDQFDV------ 125

XP_006682495.1 Bd (Ch) FFKKGI-RP-IWED--NLTGGKWIIRLKKG----IASRYWEDLLLAIVGDQFDV------ 137

XP_040746761.1 Lp (Zo) LFRDSV-RP-VWEDPANMNGGKWMIRLRKG----LATRLWERLAMAVVGDVFDV------ 96

XP_051389111.1 Ka (Zo) MFRSGV-RP-VWEDSTNMHGGKWMIRLKKG----LSPRLWEKLAMAVVGDVFGV------ 112

XP_025189698.1 Ri (Mu) LFKQGV-RP-VWEDDTNINGGKWIVRLKKG----LASRYWESLVMAVIGDQFDV------ 146

XP_051408324.1 Gm (Mu) LFKQGV-RP-VWEDETNIHGGKWIVRLKKG----LASRYWEDLVIAVIGDQFEV------ 247

XP_021883259.1 Lt (Mu) LFKQGV-RP-VWEDATNINGGKWIVRLKKG----LASRYWENLVMAVIGDQFDV------ 266

XP_051411806.1 Gm (Mu) LFKKGV-RP-VWEDPVNINGGKWIVRLKKG----LASRYWENLVMAVIGDQFDV------ 128

XP_021882609.1 Lt (Mu) LFKQGV-RP-VWEDPANISGGKWIVRLKKG----LASRYWENLAMAVIGDQFDV------ 293

**P P m**

Human 4EHP ---------------------------------------GEEICGAVVSVRFQEDIISIW 183

Drosophila 4EHP ---------------------------------------GDEICGVVLQTKYPEDSLSVW 173

C.elegans IFE-4 ---------------------------------------GDELCGAVCSIRNQEDIISLW 159

Arabidopsis nCBP ---------------------------------------ADNICGAVLSVRFNEDIISVW 170

XP_008074945.1 Vcf (Ro) ----------------------------------------IDVNGIVISFRMKNVILAVW 129

XP_047772122.1 Nm (Ro) ----------------------------------------MDVNGAVISVRGHHTILSLW 132

XP_052905898.1 Na (Ro) ----------------------------------------MEVNGIVVSVRGHHTILSLW 133

XP_013058026.1 Np (Ro) ----------------------------------------MDVNGIVVSVRGHHTILSLW 133

XP_019014281.1 Kp (Ba) ---------------------------------------SDNVVGCVLSVRQTEDILSVW 165

XP_047804977.1 Pst(Ba) DSSDS----------------SDDDQDDQDEGE-EGWEKRREICGAVLSIRRDEDILAVW 333

XP_009549304.1 Hi (Ba) AAGGGGGGGAAGSSKADEAESWRSGPKDAKEGKDVKDEEWPDICGCTISVRQSEDIVSVW 248

XP_007382918.1 Ps (Ba) -----------------------------------LDKDGPEICGCTISVRQSEDVVQLW 211

XP_031026772.1 Sm (Ch) ---------------------------------------SSEITGAVISIRNNEDILSLW 184

XP_052926586.1 Fj (Ch) ---------------------------------------GNEICGAVISIRHSEDILSLW 146

XP_006682495.1 Bd (Ch) ---------------------------------------GDEICGAVVSIRHSEDIVSLW 158

XP_040746761.1 Lp (Zo) ---------------------------------------GDEVCGIVLSIRNSEDILSLW 117

XP_051389111.1 Ka (Zo) ---------------------------------------GGEICGIVLSIRNSEDIISLW 133

XP_025189698.1 Ri (Mu) ---------------------------------------GTEICGAVLSIRSSEDILSLW 167

XP_051408324.1 Gm (Mu) ---------------------------------------GTEICGAVLSIRGSEDILSLW 268

XP_021883259.1 Lt (Mu) ---------------------------------------GSEICGAVLSIRGGEDILSLW 287

XP_051411806.1 Gm (Mu) ---------------------------------------GEEICGIVLSIRGAEDILSIW 149

XP_021882609.1 Lt (Mu) ---------------------------------------GSEICGIVLSIRGAEDILSIW 314

Human 4EHP NKTA----SDQATTARIRDTLRRVLNLPPNTIMEYKTHTDSIKMPGRLGPQRLLFQNLWK 239

Drosophila 4EHP HRTA----TDMTSTTRIRDTLRRILNIPLTTALEYKIHCDSLKYV-SINKG--------- 219

C.elegans IFE-4 NRNA----DDTPVTNRIRETLRSVLQLPQNTVLEYKRHDDCLRDQSSYR-------HTTK 208

Arabidopsis nCBP NRNA----SDHQAVMGLRDSIKRHLKLPHAYVMEYKPHDASLRDNSSYR-------NTWL 219

XP_008074945.1 Vcf (Ro) TKDSTGKDS----FKDVLMEIKKVLDVKFFLSVEYKDNDESLKDNSSFRNTKNLYV---- 181

XP_047772122.1 Nm (Ro) TKTCPSDGE----MLEQEAEIREKLEIKPVIPVAFKGNDESLKDKSSFRHIVKEKK---- 184

XP_052905898.1 Na (Ro) TKTCGTEEE----MAAQEKEIRDKLELKHAIPVTFKGNDESLKDKSSFRYIVKDKV---- 185

XP_013058026.1 Np (Ro) TKSCPSEEE----RHIQEKEIRNTLELKQIIPVSFKGNDESLKDKSSFRHTVKDKI---- 185

XP_019014281.1 Kp (Ba) VEEE----SDSVRSGALKEKILTLLSLPSTTSCEYRANRIFLEA-TSKPA--FN------///309

XP_047804977.1 Pst(Ba) HKTGTPESGDGKMAKQVKLSLQTVLQLPLNCHLVYKLNADCLSTNVDLTTIVNNI-----///473

XP_009549304.1 Hi (Ba) TRDS-----DVKVRERTREMIRRVLSLPLATVMEYKTNNDSMQDKSSFR-------NSAV 296

XP_007382918.1 Ps (Ba) NRQE-----KPEVKEKIRETMRRVLNLPASTIIEYKSNNG-------------------- 246

XP_031026772.1 Sm (Ch) NTNA----SEGRIGLRIRDTMKKLLDLPGNCTMEYKAHTAALTDGSSFK-------NTET 233

XP_052926586.1 Fj (Ch) NLSA----DEGRVNLRIRDTLKRVLNLPPNCIMEYKAHKSSVADNSSFR-------NTDL 195

XP_006682495.1 Bd (Ch) NKSA----DEGRINLRIRDTLKRVLSLPANCVMEYKAHKAAVADNSSFR-------NTET 207

XP_040746761.1 Lp (Zo) NRTA----VDAKTNVHIRDIIKQVLEVPAETIMEYKAHNDSLKDNSSFR-------NTDV 166

XP_051389111.1 Ka (Zo) NKTA----FDSRTNLHIRDTMKMTMGLPVECIMEYKAHNDSLKDNSSFR-------NTDI 182

XP_025189698.1 Ri (Mu) NQSA----HEGRINLKIRDTMKRVLNLPSETIMEYKTHNDALKDNSSFR-------NTDV 216

XP_051408324.1 Gm (Mu) NQSA----HEGRINLKIRDTMKRVLNLPADTIMEYKTHNDALKDNSSFR-------NTDV 317

XP_021883259.1 Lt (Mu) NQSA----HEGRINLKIRDTMKRVLNLPADTIMEYKTHNDALKDNSSFR-------NTDV 336

XP_051411806.1 Gm (Mu) NQSA----DEGRINLKIRDTMKRVLNLPADTIMEYKSHNDALKDNTSFR-------NTDV 198

XP_021882609.1 Lt (Mu) NKSA----DEGRINLKIRDTMKRVLDLPIDTVMEYKSHNDALKDNTSFR-------NTDI 363

**Supplementary Fig. S8.** **Sequence comparison of human 4EHP and selected Class II orthologs from different fungal *phyla*.** The core and part of carboxi-terminal of the proteins were compared. Residues involved in cap recognition (9-12) are indicated as follows: , residues binding the guanine by  –  interactions; *G*, residue recognizing the guanine ring; *P*, residues interacting with the phosphate groups; *m*, W recognizing the cap methyl group. *Asterisks* indicate W43 and W56 of the human protein used to classify the eIF4E-family members into three classes (13). Residues identical to human 4EHP are shaded in black boxes. Conservative changes are in grey boxes. Gaps are represented by dashes. /// indicates that some residues are not shown. 4EHP-specific residues are highlighted in red. Phosphorylated S209 or S251 of human/mouse eIF4E (15,16) and *Drosophila* eIF4E-1 (17), respectively, and conserved in the fungal 4EHP orthologs is highlighted in purple boxes. Human 4EHP (acc. numb. AF047695) (29); *Drosophila* *melanogaster* 4EHP/eIF4E-8 (acc. numb. CG33100) (22,23); *Caenorhabditis elegans* IFE-4 (acc. numb. AAF62414.1) (30); *Arabidopsis thaliana* nCBP (acc. numb. AF028809) (31). Fungal species: Vcf, *Vavraia culicis floridensis*; Nm, *Nematocida major*; Na, *Nematocida ausbeli*; Np, *Nematocida parisil*; Kp, *Kwoniella pini*; Pst, *Puccinia striiformis tritici*; Hi, *Heterobasidion irregulare*; Ps, *Punctularia strigosozonata*; Sm, *Synchytrium microbalum*; Fj, *Fimicolochytrium jonesii*; Bd, *Batrachochytrium dendrobatidis*; Lp, *Linderina pennispora*; Ka, *Kickxella alabastrina*; Ri, *Rhizophagus irregularis*; Gm, *Gamsiella multidivaricata*; Lt, *Lobosporangium transversale*; *Phylum* name is indicated in parenthesis: (Ro), *Rozellomycota*; (Ba), *Basidiomycota*; (Ch), *Chytridiomycota*; (Zo), *Zoopagomycota*; (Mu), *Mucoromycota*.

Human eIF4E MAT------VE-----------PETTPT---PNPPT--------------TEEEKTESNQ 26

Yeast eIF4E MSVEEVSKKFE-----------------------------------ENVSVDDTTATPKT 25

NP_594228.1 Spo MQTEQPPKESQT---------------------------------ENTVS-EPQEKALRT 26

NP_595451.1 Spo MADAEDSRHSKN-------EGFPNTSLITEKLDLLDLFGSPKVKTEREGR-PARLLEGLS 52

XP_044700341.1 Ms MTTASS-DLPT------MVEVLDEQRPLNEIPVSPPNGASDDDADAAGASDANSKEQLT- 52

XP_022384130.1 Ab MAA-----VAE-----------NGPVPVNDENTAPG----VAAPEQKEET-EKTNGDNIT 39

XP_035350724.1 Tr MAT-----ATE-----------NGAAPA----------------IDEKATAPTGENEIVT 28

XP_020123162.1 Ta MAT-----ATE-----------NGAAPA---------------PAISEKT--EQNGDVVT 27

XP_006696165.1 Tt ---------------------MSEQVDLSTIPVSPDGENKNDTN----GSQTEEVPKPIT 35

XP_031866882.1 Ve MATATAPAPAS------GDANPSSPVDLSTIPISPDGGNTVDGKK---PSGESAADEQIT 51

XP_037177009.1 Ca -----MATSVQ------TPTNPDQQVDLSTIPISPEGAEKSTET------KENDTDKDIT 43

XP_007821367.1 Mr ------MAATS------ETPNMDQQVDLSTIPISPNGKSESTEA------K--DGDKPVT 40

XP_053003228.1 Ff -----MAAAAT------ETPKMDEQVDLTTIPVDPNGKESASDI---------KDDKPVT 40

XP_007706847.1 Bz ------MAEVA------ASANSNQQVPLDTIPISPTDNSGEGEQSS---SNAANEGEIRT 45

XP_035370516.1 Lt ---------MA------ATANPQEPVPLDTIPISPEGGNMNGTKEGEEGKVPYNDSDPVT 45

XP_016226097.1 Em ------MAEVE----------LPQPVP--------------G-PVSNENAAPASSDEIKT 29

XP_033769023.1 Sp MSVEEVSKKFE-----------------------------------ENVSVDDTTATPKT 25

XP_020047073.1 Ar MT-EEVSQKLNDLTITDDKKIIDDKTTTTTLTKSTSNTNS----ATDSTTNNNAQSKEPV 55

*

* ****

Human eIF4E EVANP-EHYIKHPLQNRWALWFFKNDKS--K-TWQANLRLISKFDTVEDFWALYNHIQLS 82

Yeast eIF4E VLSDSAHFDVKHPLNTKWTLWYTKPAVDKSE-SWSDLLRPVTSFQTVEEFWAIIQNIPEP 84

NP_594228.1 Spo VFDDKINFNLKHPLARPWTLWFLMPPTP-GLE-WNELQKNIITFNSVEEFWGIHNNINPA 84

NP_595451.1 Spo AVNAETAFVKTHPLQHEWTLWFLKPPTQ-GLE-WSDLLKEIISFKTVEEFWGIFKTISKA 110

XP_044700341.1 Ms VFHSPENFNVKHPLMNKWTLWFTKPPSPKGENNWNDLLKEVVTFDSVEEFWGIYNNIAKT 112

XP_022384130.1 Ab VFHDPENFNVKHPLMHEWTLWFTKPPSGKGD-NWNDLLKEVVTFNSVEEFWGIYNNITPT 98

XP_035350724.1 Tr VFHDPENFNVKHPLMNEWTLWFTKPPSGKGD-NWNDLLKEVVTFSSVEEFWGIYNNITPT 87

XP_020123162.1 Ta VFHDPENFNVKHPLMNEWTLWFTKPPSGKGD-NWNDLLKEVVTFGSVEEFWGIYNNITPT 86

XP_006696165.1 Tt VFHDKENFNVKHPLSCRWTLWFTKPASGKGD-NWNDLLKKVITFESVEEFWGIYNNIAPV 94

XP_031866882.1 Ve VFHDKDNFNVKHPLMHKWTLWFTKPPSGKGD-NWNELLKEVITFNSVEEFWGVYNNIAPA 110

XP_037177009.1 Ca VFHDKDNFNVKHPLQNKWTLWFTKPPSGKGD-NWNDLLKEVITFNSVEEFWGVYNNIAPV 102

XP_007821367.1 Mr VFHDKDNFNVKHPLQNKWTLWFTKPPSGKGD-NWNDLLKEVITFDSVEEFWGVYNNVAPV 99

XP_053003228.1 Ff VFHDKDNFNVKHPLQNKWTLWFTKPPSGKGD-NWNDLLKEVITFDSVEEFWGVYNNVAAV 99

XP_007706847.1 Bz VFHDPENFNVKHPLMNTWTLWFTKPPSGKGD-NWAELLKEVISFDSVEEFWGIYNNITPT 104

XP_035370516.1 Lt VFHDPNNFNVKHPLMNAWTLWFTKPPSGKGD-NWNELLKEVISFDSVEEFWGIYNNITPT 104

XP_016226097.1 Em VFHDVNNFNVKHPLLNTWSLWFTKPPSSKGD-NWNDLLKEVISFDSVEEFWGVYNNITAC 88

XP_033769023.1 Sp VLSDSAHFDVKHPLNTKWTLWYTKPAVDKSE-SWSDLLRPVTSFQTVEEFWAIIQNIPEP 84

XP_020047073.1 Ar SIFSSKHFTAYHPLNSKWTLWYTKPPTNRNE-SWNDLLKPVITFSTVEEFWGIYNAIPSA 114

**G P**

Human eIF4E SNLMPGCDYSLFKDGIEPMWEDEKNKRGGRWLITLNKQQRRSDLDRFWLETLLCLIGESF 142

Yeast eIF4E HELPLKSDYHVFRNDVRPEWEDEANAKGGKWSFQLR-GK-GADIDELWLRTLLAVIGETI 142

NP_594228.1 Spo SSLPIKSDYSFFREGVRPEWEDVHNKTGGKWAFQNK-GRGGNALDEMWLTTVLAAIGETL 143

NP_595451.1 Spo SMLPAKSDYSYFLKGIRPEWEDPQNMNGGKWAYQSK-HKG-SNLDELWLYMVLAAIGETL 168

XP_044700341.1 Ms SELALKSDYHLFKAGVRPEWEDPQNKNGGKWSYQFK-EKRAVPIDELWLHVMLAAIGETL 171

XP_022384130.1 Ab SELGLKADYHLFKKGIRPEWEDPQNKHGGKWSYSFK-DKRSVPIDDLWLHAQLAAIGETL 157

XP_035350724.1 Tr SELGLKADYHLFKKGIRPEWEDQQNKHGGKWSYSFK-DKRAVPIDELWLHAQLAAIGETL 146

XP_020123162.1 Ta SELGLKADYHLFKKGIRPEWEDQQNKHGGKWSYSFK-DKRAVPIDELWLHAQLAAIGETL 145

XP_006696165.1 Tt SELAVKSDYHLFKEGVRPEWEDPQNKHGGKWAYQFK-DKRSVNIDELWLHTMLAAIGETL 153

XP_031866882.1 Ve SELALKSDYHLFKEGVRPEWEDVQNKHGGKWAYQFK-EKRSVPIDELWLHVMLAAIGETL 169

XP_037177009.1 Ca SELALKSDYHLFKAGVRPEWEDAQNKHGGKWSYQFK-EKRAVPIDDLWLHVMLAAIGETL 161

XP_007821367.1 Mr SDLALKSDYHLFKAGVRPEWEDPQNKHGGKWSFQYK-EKRNVDIDRLWLQVMMGAIGETL 158

XP_053003228.1 Ff SELSLKSDYHLFKAGVRPEWEDPQNKHGGKWSYQYK-DKRNIDVDRLWLQVMMAAIGETL 158

XP_007706847.1 Bz SDLALKSDYHLFKKGVRPEWEDSQNKHGGKWAFQFK-DKKAINIDALWLHVMLAAIGENL 163

XP_035370516.1 Lt SQLALKSDYHLFKKGVRPEWEDPQNKHGGKWSFSFR-DKKLIDIDSLWLHVMLAAIGETL 163

XP_016226097.1 Em SDLGLKSDYHLFKKGVRPEWEDPQNKHGGKWSYQYK-DKKLINIDELWLHTQLAAIGETL 147

XP_033769023.1 Sp HELPLKSDYHVFRNDVRPEWEDEANAKGGKWSFQLR-GK-GADIDELWLRTLLAVIGETI 142

XP_020047073.1 Ar NELPIKSDYHLFKEGIRPEWEDVVNSKGGKWAYQFK-EKSKVDINDLWMRSVLALIGETI 173

**P P m**

Human eIF4E DDY-SDDVCGAVVNVRAKGDKIAIWTTEC---------------------ENREAVTHIG 180

Yeast eIF4E DED-DSQINGVVLSIRKGGNKFALWTKSE----------------------DKEPLLRIG 179

NP_594228.1 Spo DPT-GQEVMGVVINMRKGFYRLAVWTKSC---------------------NNREVLMEIG 181

NP_595451.1 Spo DPT-GKEVTGVVCNMRKGFYRIAVWTRNC---------------------NDKDVLEKIG 206

XP_044700341.1 Ms EAEDDKEIMGVVVNVRKAFFRIGVWTKSSGG---------------GKTAPSRESLMEIG 216

XP_022384130.1 Ab ENDGDSEVMGVVVNVRKGFYRVGLWTRTVGKSIPGDKQ-------ARTPAQGKDVLESIG 210

XP_035350724.1 Tr ENDDDKEVMGVVVNVRKGFYRIGLWTRTVGKSLPNDKS-----TEGRSVAKGKEVLESIG 201

XP_020123162.1 Ta ENDEDNEVMGVVVNVRKGFYRIGLWTRTVGKSLPGDKS-----TGARSAAKGKEVLENIG 200

XP_006696165.1 Tt EDEEDGEVMGVVVNVRKGFYRIGVWTRTTGRHIASRGDGDVAGGKGRSLEKSKEILMNIG 213

XP_031866882.1 Ve EEEGDGEVMGVVVNVRKGFYRVSVWTRTIGKSIPNGGDGDVAGGKGRSVEKGKEILMNIG 229

XP_037177009.1 Ca EGEDDGEVMGVVVNVRKAFFRIGVWTRTIGKSIPGRGDGDVAGGKGRSQDKGREILLNIG 221

XP_007821367.1 Mr EEEDDGEVMGVVVNVRKAFYRIGVWTRTIGKSIPGRGDGDVAGGKGRSGEKSKDILMSIG 218

XP_053003228.1 Ff EDEDDGEVMGVVVNVRKAFFRIGVWTRTIGKSIPGRGDGDVAGGKGRSNEKGKEILMAIG 218

XP_007706847.1 Bz EDEDDNEVMGVVVNVRRGFYRIGLWTRSVGRAIPGD------GGKGRTQEQGKEVLLKIG 217

XP_035370516.1 Lt EDEGDGEVMGVVVNVRKGFYRIGLWTRTVGKPFPGGGDGNNAGGKGRSFEQGKDVLMKIG 223

XP_016226097.1 Em EDEGDNEIMGVVVNVRKGFYRIGLWTRTTGKA------------------SGRDTLLKIG 189

XP_033769023.1 Sp DED-DSQINGVVLSIRKGGNKFALWTKSE----------------------DKEPLLRIG 179

XP_020047073.1 Ar EENEENEVNGVVLNVRKVFYRIGLWTKSC----------------------DKKSLEPIG 211

Human eIF4E RVYKERLGLPPKIVIGYQSHADTATKSGSTT-KNRFVV---------------- 217

Yeast eIF4E GKFKQVLKLTDDGHLEFFPHSSANGRHPQ---PSIT-L---------------- 213

NP_594228.1 Spo TRFKQVLNLP-RSETIEFSAHEDSSKSGSTRAKTRMSV---------------- 218

NP_595451.1 Spo LRFKEVLGISD-KETIEYSAHEDSSKAGSMRAKTRMSL---------------- 243

XP_044700341.1 Ms KKFKEVLRLN-TNEHCEFSGHTESAHSGSTRAKAKFVV---------------- 253

XP_022384130.1 Ab RRFKEVLRLN-EADVVEFSGHTDSAHSGSTRRRPSTLFEAFTTSGASHFTICPR 263

XP_035350724.1 Tr RRFKEVLRLK-EADIVEFSGHTDSAHSGSTRAKAKYTV---------------- 238

XP_020123162.1 Ta RRFKEVLRLKD-ADVVEFSGHTDSANAGSTRAKAKYTV---------------- 237

XP_006696165.1 Tt RRLKEVLKLPPN-EMVEFSGHTEAAQAGSTRAKARMVV---------------- 250

XP_031866882.1 Ve RRFKEVLKLP-SSEAVDFSGHTDSAHSGSTRAKAKYTV---------------- 266

XP_037177009.1 Ca RRFKEILNLPP-GESVEFSGHTDSAHSGSSRAKAKHTV---------------- 258

XP_007821367.1 Mr RRFKEVLELP-NNEQVEFSGHSDSAHAGSTRAKAKYTV---------------- 255

XP_053003228.1 Ff RRFKEVLELP-ATEQVEFSGHTDSAHSGSTRAKAKHVV---------------- 255

XP_007706847.1 Bz RRFKQALQLKD-NDVVEFSGHTDAAHAGSTRAKAKFSV---------------- 254

XP_035370516.1 Lt ARFKEALTLK-ENDQVEFSGHTDSAHSGSTRAKAKFVV---------------- 260

XP_016226097.1 Em QRFKEILQLP-ASEQLEFSGHTDAAHAGSTRAKAKYTV---------------- 226

XP_033769023.1 Sp GKFKQVLKLTDD-GHLEFFPHSSANGRHPQ---PSITL---------------- 213

XP_020047073.1 Ar KKFKTILKIRDD-DQVEFTSHDDGDTTASK---KSFHV---------------- 245

**Supplementary Fig. S9.** **Sequence comparison of human eIF4E and selected Class I orthologs from the *phylum Ascomycota****.* Full-length proteins were compared.Residues involved in the cap recognition (9-12) are indicated as follows: , W binding the guanine by  –  interactions; *G*, residue recognizing the guanine ring; *P*, residues interacting with the phosphate groups; *m*, W recognizing the cap methyl group. *Asterisks* indicate W43 and W56 of the human protein used to classify the eIF4E-family members into three classes (13). Residues identical to human eIF4E are shaded in black boxes. Conservative changes are in grey boxes. Purple boxes highlight conservation of phosphorylated amino acids S2, S15, and S28 of *S. cerevisiae* eIF4E (14), and S209 of human/mouse eIF4E (15,16). Phosphorylation of *S. cerevisiae* S28 was demonstrated to increase eIF4G affinity (18). Phospho-mimicking E or D residues in the phosphorylatable positions (18,19) are also highlighted in gray boxes. Gaps are represented by dashes. Human eIF4E (acc. numb.M15353) (20); *Ascomycota* species: *S. cerevisiae* eIF4E(acc. numb. NP_014502.1) (25,26); Spo, *Schizosaccharomyces pombe* eIF4E (27,28); Ms, *Morchella sextelata*; Ab, *Aspergillus bombycis*; Tr, *Talaromyces rugulosus*;Ta, *Talaromyces atroroseus*; Tt, *Thermochaetoides thermophila*; Ve, *Venustampulla echinocandica*; Ca, *Colletotrichum aenigma*; Mr, *Metarhizium robertsii*; Ff, *Fusarium falciforme*; Bz, *Bipolaris zeicola*; Lt, *Lasiodiplodia theobromae*; Em, *Exophiala mesophila*; Sp, *Saccharomyces paradoxus*; Ar, *Ascoidea rubescens*.

Human 4EHP ------------------------------------------------------------ 0

Human eIF4E ------------------------------------------------------------ 0

Yeast eIF4E ------------------------------------------------------------ 0

XP_002620320.1 Bg ------------------------------------------------------------ 0

XP_045288368.1 Hc ------------------------------------------------------------ 0

XP_018226759.1 Pc ------------------------------------------------------------ 0

XP_056066498.1 Dv MDSRDNLWTRRSNTSKLSLSMSSGDGKSD---PP------QRTFSATKRFGDT-SSHG-G 49

XP_007787855.1 Ep -MDNANLWTRRSNTGKLSLSMTG-DNKDGSKIDS------PRNSGSNRRLGDS-SSHG-K 50

XP_007756887.1 Cy -MENANLWTRRSNTGKLSLSMRDQDGKDGGKIES------PRSY-SSRRFGDT-SSHG-K 50

XP_003235488.1 Tr -MENANLWTRRSNSAKLSLSTSSSDSKDGAGKLD------SSRG--RSAGRFD-GSHP-R 49

XP_001242649.1 Ci -MENANLWTRRSNSSRLSLSVSDNRDSHGRSDSP------RSSS--K-RFG---DGHG-R 46

XP_056548464.1 Pe -MDNANLWTRRANSSKLSLSMSGTDGKDGARVEL------PRSS--K-RFAPD-SSHG-R 48

XP_031911590.1 Ap -MDNANLWTRRSNSSKLSLSMTGTDGKDGARVEI------PRT---K-RFGPD-SSHG-R 47

XP_040616414.1 Sb ---MDSLYGRRTNSNKLSLSTNTSPSIGSFGDAPAASPSTTRNTTLPRRFGGDSSSHGGK 57

XP_003661498.1 Tt ---MDNLWSRRANSSKLSLSTGSGQD------SP------SGRNSSFKRFGGDSSSLGK- 44

Human 4EHP -----------------------------------------------MNNKFDALKD-DD 12

Human eIF4E -----------------------------------MA----TVEPE-------------- 7

Yeast eIF4E ------------------------------------------------------------ 0

XP_002620320.1 Bg -----------------------------------------------MDNPKIV------ 7

XP_045288368.1 Hc -----------------------------------------------MDNPNVF------ 7

XP_018226759.1 Pc -------------------------MSQESDYFFSLTSS--NITP---SNE--------- 21

XP_056066498.1 Dv RNPFNALSPITAGGLASPTTSGQTAFGLGSGAFASFGSA--NKTPKTPGTAFDFKTATMS 107

XP_007787855.1 Ep SNPFNAISPLAT---NSPSAGASSAFGLGSGAFASFGTV--K-TPKTPGAAGVFDLNGRD 104

XP_007756887.1 Cy TNPFNALSPAAT---KSPSAGASSAFGLGSGAFASFGSA--K-TPKTPGTGPAFDFSLRE 104

XP_003235488.1 Tr SNPFNAISPLSS-TVSSPSVAASSAFGLGSGAFASFGST--AKSSKTANSPDAI--AGKQ 104

XP_001242649.1 Ci SNPFNAISPLST-GVSSPSTNASSAFGLGSGAFASFGSA--AKTPKTPGAF-DF--N--S 98

XP_056548464.1 Pe SNPFNAISPLSGGGVSSPSTNASSAFGLGSGAFASFGAP--K----TPAGSSEV--K--T 98

XP_031911590.1 Ap SNPFNALSPLSG-GVSSPSTNASSAFGLGSGAFASFGAP--K----TPGGS-DL--K--T 95

XP_040616414.1 Sb ANPFNTATTPGGSRIMSPGGPASSAFGLGSGAFSSFGSS--AKTPKSPGNPFDVGFKSAT 115

XP_003661498.1 Tt -HTFGSVTTPGGS-LASPIGGASSAFGLGSGAFASFGSAKTPKTPKSPGNPFDAALGAAA 102

*

Human 4EHP SGDHDQNEENSTQKDGEKE-----------KTERDKNQSSSKRKA-VVPGPAEHPLQYNY 60

Human eIF4E --------------------TTPTPNPPTTEEEKTESNQEVANPEHY----IKHPLQNRW 43

Yeast eIF4E -----MSVEEVSK------------KFEENVSVDDTTATPKTVLSDSAHFDVKHPLNTKW 43

XP_002620320.1 Bg -------------------TPDLEPAAENR-S-----SSRKTLHQKILGKLRPLPLQYHW 42

XP_045288368.1 Hc -------------------NTDPEPAAENG-SGV-VPASRRTLHQNILGKLRPLPLQYHW 46

XP_018226759.1 Pc -----------------------SPILKTSQFKKSDACQNRNIQSSIYTQSPVHMLKYSF 58

XP_056066498.1 Dv GGAPTTPGEKKEKPAGKIVNSARKE------SLSITASEEAPVSSAPLDFSVPWPLKYTW 161

XP_007787855.1 Ep ------RGGKDTNVEAVDS----------DRSKS---KTPNTTSATSNSNNSDHPLRSTW 145

XP_007756887.1 Cy ------KIEKKEKDSDIPA----------ETS----EAEPSQDSSSSSKTSSEHPLRSTW 144

XP_003235488.1 Tr ------QGDKRDLQ-ADQEGGAWKPVKSKA-SSSSLDKSASSANKDN--GVRELPLKSTW 154

XP_001242649.1 Ci ------SKSRTEKQDGEHSGTGSKSVK----SKG----SSSSLNSTNTSGPKEHPLKSTW 144

XP_056548464.1 Pe ------PGEKRDL-SVEHE----------ELSRS-------KALANAPAGPGEHALKSIW 134

XP_031911590.1 Ap ------PLEKRDN-LAEHD------------SAEGA------KMKAASSTIKEHPLKSTW 130

XP_040616414.1 Sb SGTSA-PAAAGEK--SSA--ATPRPAAVGGAAAPGAAGTAAADAAPPADKNQRYLLRNEW 170

XP_003661498.1 Tt KTPTA-EKSAKDGASGGK--SISKPAFAT--SLKEAG---TKTATPSAGPS-THQLRNCW 152

*

****

Human 4EHP TFWYSRRTPGRPTSSQSYEQNIKQIGTFASVEQFWRFYSHMVRPGDLTGHSDFHLFKEGI 120

Human eIF4E ALWFFKNDK-----SKTWQANLRLISKFDTVEDFWALYNHIQLSSNLMPGCDYSLFKDGI 98

Yeast eIF4E TLWYTKPAVDK---SESWSDLLRPVTSFQTVEEFWAIIQNIPEPHELPLKSDYHVFRNDV 100

XP_002620320.1 Bg TVWYEKHSESSN-----YVERLYVLHEDVADIGTFYRVYNNYPWHKVKLRDTVHIFRKGT 97

XP_045288368.1 Hc TVWYEKHSESAN-----YDERLYILHEDVADIGTFYRVYNNYPWHKVKLRDTVHIFRKGT 101

XP_018226759.1 Pc TFWYMNRAFG-S-RIKDYESSLKKLITFSSVEEFWSIYVYLKRPDTLPVISDLHLFKEGI 116

XP_056066498.1 Dv VIWYRPPTPK----NSDYEKSTKALCRMSTVQEFWKVFIHLKRPSTLPTVSDYHFFKEGI 217

XP_007787855.1 Ep NLFYRPPTSK----YSDYEKSTIKLASISTVETFWTIYSHLKRPSQLPSVSDYHIFKEGI 201

XP_007756887.1 Cy NLFYRPPANK----FSDYEKSTQRLASISSVESFWTIYSHLKRPSLLPTVSDYHIFKDGI 200

XP_003235488.1 Tr IVWYRPPTPK----YSDYEKSTIPLASISSVESFWTVYSHLKRPSLLPTVSDYHIFKKNI 210

XP_001242649.1 Ci VVWYRPPTPK----YSDYEKSTIALASISSVESFWAVYSHLKRPSLLPTVSDYHIFKKGI 200

XP_056548464.1 Pe IVWYRPPTPK----YSDYEKSTVPLASISSVESFWAVYSHLKRPSLLPTVSDYHIFKKGI 190

XP_031911590.1 Ap VIWYRPPTPK----YSDYEKSTVPLASIFSVESFWSIYSHLKRPSLLPTVSDYHIFKKGI 186

XP_040616414.1 Sb VFWFRPPISKAN-GYIEYEKTLHPVATVSSVEDFFAVYTHLKRPSTLPLVADYHFFRNGI 229

XP_003661498.1 Tt VFWFRPPISKAN-GFIEYEKTLHPIAAVDCVEHFFKIYQHLKRPSTLPLVSDYHLFKKGI 211

**G**  **P**

Human 4EHP KPMWEDDANKNGGKWIIRLRKG----LASRCWENLILAMLGEQFM-----VGEEICGAVV 171

Human eIF4E EPMWEDEKNKRGGRWLITLNKQQRRSDLDRFWLETLLCLIGESFD----DYSDDVCGAVV 154

Yeast eIF4E RPEWEDEANAKGGKWSFQLRGKG--ADIDELWLRTLLAVIGETID----EDDSQINGVVL 154

XP_002620320.1 Bg KPVWEDPENQKGGRWTFRVPKA----KSQAFFHEVVILCMANEFQAALEAEHDHVLGIST 153

XP_045288368.1 Hc KPVWEDPENQKGGRWTFRVPKA----KSQAFFHEVAILCMANEFQAALEAEHDHVLGVST 157

XP_018226759.1 Pc RPIFEDPANQDGGKWTLRLRKG----IVVRYWEQLVLAIVGDQFW----NVGDELCGIVL 168

XP_056066498.1 Dv RPVWEDDENKRGGKWIMRLKKG----VADRYWEELLMAVTGGEFM----EATEEVCGFVL 269

XP_007787855.1 Ep RPVWEDEANKRGGKWIVRLKKG----VADRYWEDLLFAIVGDQFM----EAGEEVCGAVL 253

XP_007756887.1 Cy RPVWEDEANKKGGKWIVRLKKG----VADRYWEDLLLAIIGDQFM----EAGEEVCGAVL 252

XP_003235488.1 Tr RPVWEDEANKRGGKWIIRLKKG----VADRYWEDLLLAMIGDQFA----EASDEVCGAVL 262

XP_001242649.1 Ci RPVWEDQANKKGGKWIVRLKKG----VADRYWEDLLLAMIGDQFA----EASDEVCGAVL 252

XP_056548464.1 Pe RPVWEDEANKRGGKWIVRLKKG----VADRYWEDLLLAMVGDQFA----EAGDEVCGAVL 242

XP_031911590.1 Ap RPVWEDDANKKGGKWVVRLKKG----VADRYWEDLLLAMVGDQFA----EAGDEVCGAVL 238

XP_040616414.1 Sb RPIWEDAENRKGGKWIVRLKKG----VADRYWEDLLFAIIGDQFG----DASDEVCGAVL 281

XP_003661498.1 Tt CPIWEDEENKNGGKWVVRLRKG----VADRYWEDLLLAVIGDQFG----EASEEVCGVVL 263

**P P m**

Human 4EHP SVRFQEDIISIWNKTASDQATTARIRDTLRRVLNLPP----NTIMEYKTHTDSIKMPGRL 227

Human eIF4E NVRAKGDKIAIWTTECENREAVTHIGRVYKERLGLPP----KIVIGYQSHADTATKSGST 210

Yeast eIF4E SIRKGGNKFALWTKSE-DKEPLLRIGGKFKQVLKLTD----DGHLEFFPHSSANGRHPQP 209

XP_002620320.1 Bg SVRFNSHLISIWNKSGSNLKAIKAIEDTVIQRLSPDLRPLSAQSYFYKRHDEHEGFEAAM 213

XP_045288368.1 Hc SVRFNSHLISVWNKLGSNPKAIKALEDTVLERLSPDLRPSSTQSYFYKRHDEHEGFKAAV 217

XP_018226759.1 Pc SIRNSEDLISVWNKSSDNGRVSLKIRDLIKQILNLPL----DTVLEYKGHKDSFVDIYNH 224

XP_056066498.1 Dv SVRSGEDVFSIWTKN--DGGRNIKIRETVKRVLNLPE----GTIITWRSHDESIAQRTAI 323

XP_007787855.1 Ep SVRSGEDVLNVWTKN--DGGRNVKIRETIKRLLAFPA----DTNIVWKSHDDSIAQR-SA 307

XP_007756887.1 Cy SVRSGEDVLSVWTKN--DGGRNIKIRETIKRVLAFPP----DTNIVWKSHDDSIAQR-SA 306

XP_003235488.1 Tr SVRSGEDVLSIWTKI--DGGRNIKIRETIKRLLAFPP----DTNIIWKSHDDSIAQR-SA 316

XP_001242649.1 Ci SVRSGEDVLSVWTRI--DGGRNIKIRETMKRLLNFPP----DTNIVWKSHDDSIAQR-TA 306

XP_056548464.1 Pe SVRGGEDVLSVWTKI--DGGRNIKIRETIKRLLAFPA----DTNIVWKSHDDSIAQR-SA 296

XP_031911590.1 Ap SVRSGEDVLSVWTRI--DGGRNIKIRETIKRLLSFPI----DTNIVWKSHDDSIAQR-SA 292

XP_040616414.1 Sb SVRNGEDILSVWTRT--DGSRVLKIRETMRRALGFPL----ETKMDWKSHDSSIQQR-SA 335

XP_003661498.1 Tt SVRNGEDILSIWARA--NGQRVLKIRETMRRILSFPP----DTKLEWKSHDSSIQQR-TA 317

Human 4EHP -GPQRLLFQNLWKP--RLNVP---------------------------------------- 245

Human eIF4E -TKNRFVV----------------------------------------------------- 217

Yeast eIF4E -SITL-------------------------------------------------------- 213

XP_002620320.1 Bg -EEHKAAVEEHE---------------------------------------APTSSGERRN 234

XP_045288368.1 Hc -EENAGATS----------------------------------------------SGKSGK 231

XP_018226759.1 Pc -YSSD--KNSRLYE--KTNTTKHKVIDN--------------------------------- 247

XP_056066498.1 Dv -DQARQDKGHQEKRRPNTSNTEE----SSK------------------------------- 348

XP_007787855.1 Ep LDEARQQKGGSTQV--------------------------QQGHDKRRQTLQEDPEKGKGR 341

XP_007756887.1 Cy LDEARQQKVSGTPG--------------------------HPGV-QRRSTLRDEPERSKEK 339

XP_003235488.1 Tr IDQARLDKSSGNSGHH------------------HHHHHHNQGADRRRITNQEDSTTERGK 358

XP_001242649.1 Ci IDQARQDKAANSGH--------------------------HQGSDRRRGANPDDSTGEKGK 340

XP_056548464.1 Pe IDQARHEKAHTGGSNPTA----------------------LQGSERRRTG-HDE--SDKGK 331

XP_031911590.1 Ap IDQARQEKAAGNNSHHHHHHHHH----NNN--NNNNNQHHNLGAERRRVTANDDSTGDKGK 346

XP_040616414.1 Sb IEESRRDKSNQHHNSGGTNNQHHNNHHNNQHHNNHHNHNHNNYNQHQHQHQNHQSDRRSGK 395

XP_003661498.1 Tt IEESRREKANQHHHGDKRSSNKQ---HQQQHS-----QQQQQQQQ-QQQQQQQQADDQ--K 366

Human 4EHP --------------------------- 245

Human eIF4E --------------------------- 217

Yeast eIF4E --------------------------- 213

XP_002620320.1 Bg GP------------------------- 236

XP_045288368.1 Hc GS------------------------- 233

XP_018226759.1 Pc --------------------------- 247

XP_056066498.1 Dv --------------------------- 348

XP_007787855.1 Ep GPL------------------------ 344

XP_007756887.1 Cy GTQ------------------------ 342

XP_003235488.1 Tr NTTNS---------------------- 363

XP_001242649.1 Ci GSAS----------------------- 344

XP_056548464.1 Pe GSTAL---------------------- 336

XP_031911590.1 Ap GAAS----------------------- 350

XP_040616414.1 Sb LPYSARASATTSGGGGDDQQLHAHGTP 422

XP_003661498.1 Tt NPTNSS--------------------- 372

**Supplementary Fig. S10.** **Sequence comparison of human 4EHP and selected Class II orthologs from the *phylum Ascomycota****.* Human and yeast *S. cerevisiae* eIF4Es were also included to identify the reported phosphorylated residues (14-16).Full-length proteins were compared.Residues involved in the cap recognition are indicated as follows: , residues binding the guanine by  –  interactions; *G*, residue recognizing the guanine ring; *P*, residues interacting with the phosphate groups; *m*, W recognizing the cap methyl group. *Asterisks* indicate residues equivalent to W43 and W56 of the human protein used to classify the eIF4E-family members into three classes (13). Residues identical to human 4EHP are shaded in black boxes. Conservative changes are in grey boxes. Changes of W56 to Y and the change K/R162 to I, V, or L that define Class II proteins are highlighted in red. Gaps are represented by dashes. Purple boxes highlight conservation of phosphorylated amino acids S2, S15, and S28 of *S. cerevisiae* eIF4E (14), and S209 of human/mouse eIF4E (15,16). Phosphorylation of *S. cerevisiae* S28 was demonstrated to increase eIF4G affinity (18). Phospho-mimicking E or D residues in the phosphorylatable positions (18,19) are also highlighted in gray boxes. Human 4EHP (acc. numb. AF047695) (29); Human eIF4E (acc. numb.M15353) (20); *Ascomycota* species: *S. cerevisiae* eIF4E (acc. numb. NP_014502.1) (25,26); Bg, *Blastomyces gilchristii*; Hc, *Histoplasma capsulatum*; Pc, *Pneumocystis carinii*; Dv, *Didymosphaeria variabile*; Ep, *Endocarpon pusillum*; Cy, *Cladophialophora yegresii*; Tr, *Trichophyton rubrum*; Ci, *Coccidioides immitis*;Pe, *Penicillium canariense*; Ap, *Aspergillus pseudotamarii*; Sb, *Sporothrix brasiliensis*; Tt, *Thermothelomyces thermophilus*.

Human eIF4E -----MA---------------------TVEPETTPTPNPPTTEEEKTESNQEVANPEHY 34

Yeast eIF4E -----MSVEEVSKKFEENVSV----------DDTTATPKTV------LSDSAHFD----- 34

XP_035350331.1 Tr -----MPEPIIQISSPE-------------DYPTPNRAPPPTRK----TLHQN--ILGKL 36

XP_002543851.1 Ur -----MSNPTIQTTGIDPEP----------EASSNDTKLPATRK----TLHQN--IIGKL 39

XP_003067007.1 Cp MPSSTMSNPSIRTTGPDLQP----------DESNDDTKSPATRK----SLHQN--IIGKL 44

XP_025536811.1 Ac -----MSTIPSVHINDK--SPIEDRD--------TSSTSPTTRK----SLHQN--IFGKL 39

XP_024698967.1 As -----MPLPKLHINDASPVTDELAS-------PEEPTITPISRK----SLHQN--IFGKL 42

XP_041149499.1 Af -----MALPRVHVNDKSPLSENSNPKDSDINKRPSAQIIAPTRK----SLHQN--IFGKL 49

XP_002795454.1 Pl -----MNIPKNLGTNAEPVA-----------KDGSSITPPISRK----TLHQK--ILGKL 38

XP_010757654.1 Pb -----MNIPKNLGTNAEPVA-----------KDGSSLAPPISRK----TLHQK--ILGKL 38

XP_003175831.1 Ng -----MTIQPPKFTALEPVS-----------EDECHAL-NPTRK----TLHQN--IIGKL 37

XP_003018918.1 Tv -----MTIQPPKFTALEPVT-----------EDEVQAL-NPTRK----TLHQN--IIGKL 37

*

* ****

Human eIF4E IKHPLQNRWALWFFKNDKSK-------------TWQANLR-LISKFDTVEDFWALYNHIQ 80

Yeast eIF4E VKHPLNTKWTLWYTKPAVDKS---------ES--WSDLLRPVTS-FQTVEEFWAIIQNIP 82

XP_035350331.1 Tr RPLPLQYHWTFWFDKHSEPVPS------SSPEEEYGSRLTVLYEDVADIATFYRVYNNYP 90

XP_002543851.1 Ur RPLPFQYRWVVWHEKHV-------------ESANYHDRLYLLHEDVADIATFYRIYNNYP 86

XP_003067007.1 Cp RPLPFQYRWVVWYEKQV-------------ESTNYDERLYLLHGDVADIATFYRVYNNYP 91

XP_025536811.1 Ac RPLPFQYHWTVWYDRHTDAPAAAASASGATATADYDSRLYVLHEDVADIATFYRVYNNYP 99

XP_024698967.1 As RPLPFQYHWTVWYDKHSDPP------------GDYENRLYVLHEDVADIATFYRVYNNYP 90

XP_041149499.1 Af RPLPFQYHWTVWYDKHS-------------DSTDYDNRLYVLHEDVADIATFYRVYNNYP 96

XP_002795454.1 Pl RPLPFQYHWTVWYEKHSK-------------SANYDERLFVLHEDVADIGTFYRVYNNYP 85

XP_010757654.1 Pb RPLPFQYHWTVWYEKHSK-------------SVNYDERLFVLHEDVADIGTFYRVYNNYP 85

XP_003175831.1 Ng RPLPFQYRWAVWHEKHS-------------ESVNYGDRLYPLHDDVADIATFYRIYNNYP 84

XP_003018918.1 Tv RPLPFQYRWAVWHEKHS-------------ESTNYGDRLYPLHDDVADIATFYRIYNNYP 84

**G P**

Human eIF4E LSSNLMPGCDYSLFKDGIEPMWEDEKNKRGGRWLITLNKQQRRSDLDRFW-LETLLCLIGE 140

Yeast eIF4E EPHELPLKSDYHVFRNDVRPEWEDEANAKGGKWSFQLRGKGA--DIDELW-LRTLLAVIGE 140

XP_035350331.1 Tr WDK-IRPRDTVHIFRKGIKPVWEDPDNLNGGCWTFRVPKANSQP----FFHELAILVMASE 146

XP_002543851.1 Ur WEK-VRLRDSVHIFRKGTKPIWEDPENVNGGCWTFQVPKAKSQS----FFHELAILCMANE 142

XP_003067007.1 Cp WDK-VKLRDSVHIFRKGTKPIWEDPENANGGCWTFQVPKAKCHA----FFHELAILCMANE 147

XP_025536811.1 Ac WDK-IRLRDTVHIFRKGVRPVWEDPENQNGGCWRFRVPKSKAQA----FFHEIAILCMANE 155

XP_024698967.1 As WNK-VRLRDTVHIFRKGVRPVWEDPENLKGGCWRFRVPKSKAQA----FFHEIAILCMANE 146

XP_041149499.1 Af WDK-IRLRDTVHIFRKGVRPVWEDQENLKGGCWRFRVPKSKAQE----FFHEIAILCMANE 152

XP_002795454.1 Pl WDK-VKLRDTVHIFRKGTKPVWEDPENFKGGCWTFRVPKAKSQA----FFHEIAILCMANE 141

XP_010757654.1 Pb WDK-VKLRDTVHIFRKGTKPVWEDPENLKGGCWTFRVPKVKSQA----FFHEIAILCMANE 141

XP_003175831.1 Ng WEK-IRARDTVHIFRRETKPVWEDPQNLKGGCWTFRVPKSKSQA----FFHEVAILCMANE 140

XP_003018918.1 Tv WGK-IKVRDTVHIFRRETKPVWEDPQNLKGGCWTFRVPKSKSQA----FFHEVAILCMANE 140

**P P m**

Human eIF4E ---SFDDYSDDVCGAVVNVRAKGDKIAIWTTECENREAVTHIGRVYKERLGLPPKIV--- 194

Yeast eIF4E ---TIDEDDSQINGVVLSIRKGGNKFALWTK-SEDKEPLLRIGGKFKQVLKLTDDGHLEF 196

XP_035350331.1 Tr LQAAIEGEHDHVLGVSTSTRYNNNLISVWNKHGSNEKSIKILENTILNRLSPELRPIPN- 205

XP_002543851.1 Ur LQAAVQGEHDHVLGVSTSVRFKTHLISVWNKSGRNEKSIRALGDTIIERLSEDLRPAST- 201

XP_003067007.1 Cp LQAAVQGEHDHVLGVSSSVRFKTHLISVWNKAGRNEKSIRILGETIIERLSPELRPASS- 206

XP_025536811.1 Ac FQAVLEKEHDHVLGVSTSVRFNSHLISVWNKLGSNERSIKALEQTIIDRLSPELRPTGTG 215

XP_024698967.1 As FQAVLEKEHDHVLGVSTSVRFNSHLISVWNKLGSNEKSIKVLERTILDRLSPDLRPTGSG 206

XP_041149499.1_Af FQAVLEQEHDHVLGVSTSVRFNTHLISVWNKLGSNERSIKALERTILDRLSPELRPTGTG 212

XP_002795454.1_Pl FQAALEAEHDHVLGVSTSVRFNSHLISIWNKSGSNSKAIKALEDTIIKRLSPDLRPSST- 200

XP_010757654.1_Pb FQAALEAEHDHVLGVSTSVRFNSHLISIWNKSGSNSKAIKALEDTIIERLSPDLRPSST- 200

XP_003175831.1_Ng FQAAVQSERDHVLGVSTSARFNSNLISIWNKQGYNPKAIKALEDIILQRLSPELRPTAE- 199

XP_003018918.1_Tv FQAAVQSERDHVLGVSTSARFNSNLISVWNKQGYNLKAIKALEEVILQRLSPELRPTAE- 199

Human eIF4E ------------IGYQSHADTATKSGSTTKNRFVV------------------ 217

Yeast eIF4E ------FPHSSANGRHPQPSITL------------------------------ 213

XP_035350331.1 Tr SNSYFYKRHADREGYQGAIKQAPPDSKK------------------------- 233

XP_002543851.1 Ur -KSYYYKRHDEHDGFAAALEAVRKSQENSTS---------------------- 231

XP_003067007.1 Cp -KTYYYKRHDEHDGYEAAMKAAMKNKEESNNATNKETSQCPSKDPGGGDEAGK 258

XP_025536811.1 Ac STAYFYKRHDENEGFQEAVERSKT----------------------------- 239

XP_024698967.1 As SNSYFYKRHDENEGYQEAVEQLALKN--------------------------- 232

XP_041149499.1 Af SNAYFYKRHDENEGYQEAVERTALKD--------------------------- 238

XP_002795454.1 Pl -QSYFYKRHDEHEGYKAALETQDGSTGNSEDKTESW----------------- 235

XP_010757654.1 Pb -QSYFYKRHDEHEGYKAALETQDGSTGNSVLR--------------------- 231

XP_003175831.1 Ng -KSYFYKRHDEHDGYQAAIEAVLGSSSD------------------------- 226

XP_003018918.1 Tv -KSYFYKRHDEHDGYQAAIEAALGSATHRSNTLE------------------- 232

**Supplementary Fig. S11.** **Sequence comparison of human eIF4E and selected F-Class IV orthologs from the *phylum Ascomycota****.* Yeast *S. cerevisiae* eIF4E was also included to identify the reported phosphorylated residues (14).Full-length proteins were compared.Residues involved in the cap recognition in eIF4E (9-12) are indicated as follows: , residues binding the guanine by  –  interactions; *G*, residue recognizing the guanine ring; *P*, residues interacting with the phosphate groups; *m*, W recognizing the cap methyl group. *Asterisks* indicate residues equivalent to W43 and W56 of the human protein used to classify the eIF4E-family members into three classes (13). Residues identical to human eIF4E are shaded in black boxes. Conservative changes are in grey boxes. Purple boxes highlight conservation of phosphorylated amino acids S2, S15, and S28 of *S. cerevisiae* eIF4E (14), and S209 of human/mouse eIF4E (15,16), respectively. Phosphorylation of *S. cerevisiae* S28 was demonstrated to increase eIF4G affinity (18). Phospho-mimicking E or D residues in the phosphorylatable positions (18,19) are also highlighted in gray boxes. Y equivalent to human W56, K/R112 change to C, and K/R162 change to L that define F-Class IV proteins are highlighted in red. Gaps are represented by dashes. Human eIF4E (acc. numb.M15353) (20). *Ascomycota* species: *S. cerevisiae* eIF4E (acc. numb. NP_014502.1) (25,26); Ur, *Uncinocarpus reesii*; Ng, *Nannizzia gypsea*; Pl, *Paracoccidioides lutzii*; As, *Aspergillus steynii*; Af, *Aspergillus flavus*; Ac, *Aspergillus costaricaensis*; Tr, *Talaromyces rugulosus*; Cp, *Coccidioides posadasii*; Tv, *Trichophyton verrucosum*; Pb, *Paracoccidioides brasiliensis*.

Human eIF4E ------------------------------------------------------------ 0

Yeast eIF4E MS---------------------------------------------------------- 0

XP_033542872.1 Li ------------------------------------------------------------ 0

XP_003839901.1 Pl --MAETARFIAMIRASASQNANVDKSKDRDQSSY-----RPIREGPAAARKPTSALLLGE 53

XP_040789446.1 Cb MT--ESARLISMLRASAVHKANCDKPLDYGYSPCATTSQVPQVETAPIRLKVIPVSLLEG 58

XP_051590366.1 Ap MTMNETIRIIGMLRASAAHNADHNKISEREAFFASANESGKKLRTAAAARKPLSVLLLEG 60

XP_051328791.1 Ac MTMNETIRIIGMLRASAAQNADQAKLKEREAFLASANETRKKLRTAAAARKSTSVSLLED 60

XP_049202463.1 Av MTMNETIRIIGMLRASAAQNADQAKLKEREAFFASANETRKKLRTAAAARKSTSVSLLED 60

XP_007683573.1 Bo ------------------------------------------------------------ 0

XP_014077276.1 Bm ------------------------------------------------------------ 0

XP_056073742.1 Dv MS--ETARLIGMLKASAAQRIPYAQPLDLNLLEDPAFIS-------------------IK 39

XP_033653890.1 Wo ------------------------------------------------------------ 0

XP_038798412.1 Ar ------------------------------------------------------------ 0

XP_046001131.1 Be --MAEAIRLIAMLRASATSSANFDKPLQLDNP---TYSL-------------------ES 36

Human eIF4E ------------------------------------------------------------ 0

Yeast eIF4E ------------------------------------------------------------ 0

XP_033542872.1 Li ------------------------------------------------KETTLDVTNDLA 12

XP_003839901.1 Pl LIHSIKNGAPLPNKPLNTETKEGLDRILDLLNNSLLQTPTATPVTHLSKHK-S-TFKDID 111

XP_040789446.1 Cb LIHASKHGVPRPNQPLSVDFKVGLDQILNTLNTTFSRDASVVVPS---KETAHDLVNDLA 115

XP_051590366.1 Ap LVHATKHGVPYPSNPLTYESKVGINRIIVALNRAIPRDADIVPPNVHSKDTTQDLANDLA 120

XP_051328791.1 Ac LIYATKHGVPYPNEPLTYEAKVAADRILVTLNKAIARETILVVPNVQPKETSEDLANDLA 120

XP_049202463.1 Av LIYATKHGVPYSNEPLTYEAKVAADRILVTLNKAIARETILVVPNVQIKETSEDLANDLA 120

XP_007683573.1 Bo ------------------------------------------------------------ 0

XP_014077276.1 Bm ------------------------------------------------------------ 0

XP_056073742.1 Dv TGHTTYKGVT--------QAPNTFSEALHSIADGQPL--------PPPPN---------- 73

XP_033653890.1 Wo ------------------------------------------------------------ 0

XP_038798412.1 Ar ------------------------------------------------------------ 0

XP_046001131.1 Be D------------------TRGGLQHVLHLLRSSAPRNITSVHPHARPVETTSDLLNDLA 78

Human eIF4E -----------------------------------MATVEPETTPTPNPPTTEE------- 19

Yeast eIF4E --------------------------------------------VEEVSKKFEENV-SVDD 18

XP_033542872.1 Li VATSKHILVAAQNLQELERVACNLGVTKLENVHPSDEADDKMAGRFPLLSTSNLPAASAE- 72

XP_003839901.1 Pl C-----------VTSTRKLNTTEITTPL---------HATTMANRFPALSTSDLPRASAE- 151

XP_040789446.1 Cb IENCKRIFVVRSSTTQLENIANNLGIAL---------LSPTMAGRFPVLSTSDLPRASAE- 166

XP_051590366.1 Ap IENSKRIFTARSNIKELKLISSNLGIPV---------TSPTMAGRFPVLSTSDLPKASAE- 171

XP_051328791.1 Ac IENSKRIFTARSNIKELQLISSNLGIPI---------TSPAMAGRFPVLSTSDLPKASAE- 171

XP_049202463.1 Av IENSKRIFTARSNIKELHLISSNLGIPI---------TSPAMAGRFPVLSTSDLPKASAE- 171

XP_007683573.1 Bo -----------------------------------------MAGRFPVLSTSNLPAASSE- 19

XP_014077276.1 Bm -----------------------------------------MAGRFPALSTSNLPAASSE- 19

XP_056073742.1 Dv -APFGKEFLPRLDFVIT-TLNNHTVRTI---------SKTKMAGRLPALSTSDLPSASPS- 122

XP_033653890.1 Wo -----------------------------------------MAGRFPVLSTSNLPLASST- 19

XP_038798412.1 Ar -----------------------------------------MAGRFPVLSTSNLPPASAS- 19

XP_046001131.1 Be IATCKRIFAACLDVNKLQIIALSISTSP---------SASEMASRFPVLSTSDLPPASAS- 129

*

* ****

Human eIF4E -EK--TESNQEV----ANPEHYI-KHP-LQNRWALWFFKND--KS-----K---T--WQAN- 59

Yeast eIF4E ---TTATPK--T-VL-SDSAHFDVKHP-LNTKWTLWYTK------PAV-DK---SESWSDL- 6s

XP_033542872.1 Li EARNTASPARGTAMLNS--IFKSVRVPEFRFKWQFWAEKGQSGSTPAPKDKA-SIDEYASRP 131

XP_003839901.1 Pl EQSATASPARGTAMLNS--IFKSVRVPEFRFKWLFWAEKGQAGAP---ASKA-GAEDFASRP 207

XP_040789446.1 Cb EQAATASPARGTAMLNS--IFKSVRVPEFRFKWLFWAEKGQQSTP---KDKAANSEEYSSRP 223

XP_051590366.1 Ap EQSATASPARGTAMLNN--IFKSVRVPEFRFKWMFWAEKGQQAAP---KDKV-ASEEYLTRP 227

XP_051328791.1 Ac EQSATASPARGTAMLNN--IFKSVRVPEFRFKWMFWAEKGQQAAP---KDKA-ASEEYLTRP 227

XP_049202463.1 Av EQSATASPARGTAMLNN--IFKSVRVPEFRFKWMFWAEKGQQAAP---KDKA-ASEEYLTRP 227

XP_007683573.1 Bo EQAATASPARGTAMLNN--IFKSVRVPEFRFKWMFWAEKGQQAPP---KDKAAASEEYLTRP 76

XP_014077276.1 Bm EQAATASPARGTAMLNN--IFKSVRVPEFRFKWMFWAEKGQQAPP---KDKAAASEEYLTRP 76

XP_056073742.1 Dv EASATASPARGSAMLNS--IFKSVRVPEFKYRWQFWAEKGQQQPPPKTSSTTAGAEEYANRP 182

XP_033653890.1 Wo EQSATASPARGSAMLNS--IFKSVRVPEFRFKWQFWAEKGQQSVPSS-NDKSSTSEDFASRP 78

XP_038798412.1 Ar EQSATASPARGSAMLNS--IFKSVRVPEFRFRWMFWAEKGQQSGS---KDKTAQLEEYASRP 76

XP_046001131.1 Be EQSATASPARGSAMLNS--IFKSVRVPEFRFRWMFWAEKGQQSGP---KDKSSQSEEYASRP 186

**G P**

Human eIF4E LRLISKFDTVEDFWALYNHIQLSSNLMPGCDYSLFKDGIEPMWEDEKNKRGGRWLITLNK 119

Yeast eIF4E LRPVTSFQTVEEFWAIIQNIPEPHELPLKSDYHVFRNDVRPEWEDEANAKGGKWSFQLRG 121

XP_033542872.1 Li KPLGETIISVKEFYQHFNNIPTDSLK-LRDSIHLFHLGVKPLWEDPRNARGGAWYFKVK- 189

XP_003839901.1 Pl KPLGDTIISVKEFYQHFNNIPVENLK-LRDSIHLFHLGVKPVWEDPRNVKGGAWYFKVSK 266

XP_040789446.1 Cb KPLGEQIISVREFYQHFNNIPVESLK-LRDSIHLFHLGIKPVWEDPRNARGGAWYFKVSK 282

XP_051590366.1 Ap KPLGDQIVSVKEFYQHFNNIPVENLK-LRDSIHLFHLGVKPVWEDPRNTRGGAWYFKVSK 286

XP_051328791.1 Ac KPLGDQIVSVKEFYQHFNNIPVENLK-LRDSIHLFHLGVKPVWEDPRNTRGGAWYFKVSK 286

XP_049202463.1 Av KPLGDQIVSVKEFYQHFNNIPVENLK-LRDSIHLFHLGVKPVWEDPRNTRGGAWYFKVSK 286

XP_007683573.1 Bo KPLGDQIVSVKEFYQHFNNIPVENLK-LRDSIHLFHLGVKPVWEDPRNTRGGAWYFKVSK 135

XP_014077276.1 Bm KPLGEQIVSVKEFYQHFNNIPVENLK-LRDSIHLFHLGVKPVWEDPRNTRGGAWYFKVSK 135

XP_056073742.1 Dv KPLGEQIISVKDFYQHFNNIPVESLK-LRDSIHLFHLGVKPLWEDPRNTRGGAWYFRIGK 241

XP_033653890.1 Wo KPLGEQIITIKEFYQHFNNIPTESLK-LRDSIHLFHVGIKPLWEDPRNTRGGAYYFRIPK 137

XP_038798412.1 Ar KPLGEQIISIKEFYQHFNNIPVESLK-LRDSIHLFHMGIKPVWEDPRNARGGAWYFKVTK 135

XP_046001131.1 Be KPLGEQIISVKEFYQHFNNIPVESLK-LRDSIHLFHLGVKPVWEDPRNAKGGAWYFKVTK 245

**P P**  **m**

Human eIF4E QQRRSDLDRFWLETLLCLIGESFD--------DYSDDVCGAVVNVRAKGDKIAIWTTECE 171

Yeast eIF4E KGA--DIDELWLRTLLAVIGETID--------EDDSQINGVVLSIRKGGNKFALWTKSED 171

XP_033542872.1 Li EN----AAQIWHEICLLAVGDVLQGAVETKRETFNDDICGVSYSVRWNTVQIAVWNRDAE 245

XP_003839901.1 Pl DV----ASQFWHEMCLLAVGDILQGAVETKRVSFNDDICGISYSVRWNAVQIAVWNRDAD 322

XP_040789446.1 Cb EL----AAQFWHEMCLLAVGDILQSAVETKRDSFNDDICGITYSVRWNAVQIAVWNRDAE 338

XP_051590366.1 Ap DL----AAQFWHEMCLLAVGDVLQGAVETKRASFNDDICGISYSVRWNAVQIAVWNRDAE 342

XP_051328791.1 Ac DV----AAQFWHEMCLLAVGDVLQGAVETKRASFNDDICGISYSVRWNAVQIAVWNRDAE 342

XP_049202463.1 Av DV----AAQFWHEMCLLAVGDVLQGAVETKRASFNDDICGISYSVRWNAVQIAVWNRDAE 342

XP_007683573.1 Bo DL----AAQFWHEMCLLAVGDVLQGAVETKRASFNDDICGISYSVRWNAVQIAVWNRDAE 191

XP_014077276.1 Bm DL----AAQFWHEMCLLAVGDVLQGAVETKRASFNDDICGISYSVRWNAVQIAVWNRDAE 191

XP_056073742.1 Dv EQ----ASQFWHEICLLAVGDVLQGAVETKRASFNDDICGVSYSVRWNAVQIAIWTRDAD 297

XP_033653890.1 Wo DI----AEQFWHEMCLLAVGDVLQGAVETKRESFNDDICGISYSVRWNAVQIAVWNRDAD 193

XP_038798412.1 Ar DV----APQFWHELCLLAVGDVLQGAVETKRASFNDDICGLSYSVRWNAVQIAVWNRDAD 191

XP_046001131.1 Be EV----APQFWHELCLLAVGDVLQGAVETKRASFNDDICGLSYSVRWNAVQIAVWNRDAD 301

Human eIF4E NREAVTHIGRVYKERLG--LPPKIVIGY--QSHADTATKSG--STTKNRFVV--------- 217

Yeast eIF4E KEPLLRIGGKFKQVLKLTDDGHLEFFPHFP--HSSANGRHPQPSITL-------------- 213

XP_033542872.1 Li NKEGIDRLLQVILEKLSDDIKPKGKDSYWYKAHSSHKDYVAPAETQTRDA----------- 295

XP_003839901.1 Pl NEAGKEKLLAVILEKLSEELKPKKEDNYWYKAHREHKGFI---EQ---------------- 364

XP_040789446.1 Cb NVAGREKLLAIILDKLSPELQPKKEDSYWYKPHKEHKGFV---EQQ--------------- 381

XP_051590366.1 Ap NEDGRQKLLEVILDKLSEELRPKKEDSYWYKAHKEHKGFI---EQ---------------- 384

XP_051328791.1 Ac NEDGRQKLLAVILDKLSEELRPKKEDSYWYKAHKEHKGFI---EQ---------------- 384

XP_049202463.1 Av NEDGRQKLLAVILDKLSEELRPKKEDSYWYKAHKEHKGFI---EQ---------------- 384

XP_007683573.1 Bo NEEGRQKLLAVILDKLSEELRPKKEDSYWYKAHKEHKGFI---EQQ--------------- 234

XP_014077276.1 Bm NEEGRQKLLAVILDKLSEELRPKKEDSYWYKAHKEHKGFI---EQQ--------------- 234

XP_056073742.1 Dv NEAGKEKLLQTILEKLSPELEPKK-DSYWYKAHKEHKGFAAP-E----------------- 339

XP_033653890.1 Wo NEAGKEKLLAVILDKLSEELRPKK-ESYWYKAHKEHKGFIAPGAA---------------- 237

XP_038798412.1 Ar NKAGREKLLAVILEKLSPELQPKKEDSYWYKAHNEHKGFIAQ-EETNA------------- 238

XP_046001131.1 Be NETGREKLLKVILEKLSPELQPKKEDSYWYKAHNEHKGFITE-EAKA-------------- 347

**Supplementary Fig. S12.** **Sequence comparison of human eIF4E and selected F-Class V orthologs from the *phylum Ascomycota***. Yeast *S. cerevisiae* eIF4E was also included to identify the reported phosphorylated residues (14). Full-length proteins were compared.Residues involved in the cap recognition (9-12) are indicated as follows: , residues binding the guanine by  –  interactions; *G*, residue recognizing the guanine ring; *P*, residues interacting with the phosphate groups; *m*, W recognizing the cap methyl group. *Asterisks* indicate residues equivalent to W43 and W56 of the human protein used to classify the eIF4E-family members into three classes (13). Residues identical to human eIF4E are shaded in black boxes. Conservative changes are in grey boxes. Purple boxes highlight conservation of phosphorylated amino acids S2, S15, and S28 of *S. cerevisiae* eIF4E (14), and S209 of human/mouse eIF4E (15,16), respectively. Phosphorylation of *S. cerevisiae* S28 was demonstrated to increase eIF4G affinity (18). Phospho-mimicking E or D residues in the phosphorylatable positions (18,19) are also highlighted in gray boxes. The conservative change of W56 to Y is highlighted in gray, and the non-conservative changes R/K112 to A and the K/R162 to Q that define F-Class VI proteins are highlighted in red. Gaps are represented by dashes. Human eIF4E (acc. numb.M15353) (20). *Ascomycota* species: *S. cerevisiae* eIF4E (acc. numb. NP_014502.1) (25,26); Li, *Lindgomyces ingoldianus*; Pl, *Plenodomus lingam*; Cb, *Cucurbitaria berberidis*; Ap, *Alternaria postmessia*; Ac, *Alternaria conjuncta*; Av, *Alternaria ventricosa*; Bo, *Bipolaris oryzae*; Bm, *Bipolaris maydis*; Dv, *Didymosphaeria variabile;* Wo, *Westerdykella ornata*; Ar, *Ascochyta rabiei*; Be, *Boeremia exigua*.

Human eIF4E --MAT-VEP--------------------------------------------ETTP--- 10

Yeast eIF4E MS-----------------------------------------------VEEVSKK-FEE 12

NP_594228.1 Spo ---MQTEQPP-------------------------------------------KESQTEN 14

NP_595451.1 Spo --MADAEDSRHSKNE---------------GFPNT---SLITEKLDLLDLFGSPKVKTER 40

XP_047779270.1 Rr MSS------LEASQAA---IADSIAEN-------------KPNGMNGENGLE-PGEIQEV 37

XP_009541196.1 Hi MT-TVSSLPGPALQAARSAVQAALAEHPEANVPASLATSTIVEEKGTGDALE-PGEIQE- 57

XP_007265467.1 Fm MT-SASSLPASAMQASRAALSAALSEAKESDGTSDVTN-ASPEAATNGDDME-DGEIQE- 56

XP_037222000.1 Mi MSVLSGPAPSG----------AAL---------------SDPPKDATIDDSE-PGEVQE- 33

XP_041300350.1 Sd --MA--ALPASFQHANKAAIAAALAEN-------------SE----SIGADVE-PGEIQE- 38

XP_007763399.1 Cp MSATSSPLPPAALQASKAALSAVLAEN-----------AQTTASPENSDNE-QGEVQE- 46

XP_025600987.1 Tw --MATSALPAAPAAASSAAVSALASE-----TP---------------------ASPSAS 32

XP_029738848.1 Sg MSATTSDLPKAPADASKAALDSAVDQTVNGDAAASTSDEA--------------AAAADA 46

XP_014653600.1 Ma MS-GTTDLPKAPAEASKVALDSAVAQTLNGDAPATSEGG-------------------EA 40

XP_007406414.1 Mlp ------------------------------------------------------------ 0

XP_014568616.1 Mo MS-AATLPEPAKAAVS-ASIESAVVQA---DKA---------------------ADKQSD 34

*

Human eIF4E -TPNPPTTEEEKTESNQEVANPEHYIKHPLQNRWALWFFKND------------------ 51

Yeast eIF4E NVSVDDTTATPK---TVLSDSAHFDVKHPLNTKWTLWYTKPAVD---------------- 53

NP_594228.1 Spo TVSEPQEKALR---TVFDDKINFNLKHPLARP-WTLWFLMPPTP---------------- 54

NP_595451.1 Spo EGR-PARLLEGL---SAVNAETAFVKTHPLQHEWTLWFLKPPTQ---------------- 80

XP_047779270.1 Rr EVDMQAHAESIR---TVFNDPTNFNVKHPLYSPWALWFDSPATKGRNLPQTPMSSFPQTP 94

XP_009541196.1 Hi -VNMEAQAETIR---TVFSDPKNFNVKHPLYSPWTLWFDSPATKGRNLPQTPSTAFPQTP 113

XP_007265467.1 Fm -VDMESQAEGIR---TVFSDPTNFNVKHPLFSPWTLWFDSPSTKGRNLPQTPMTAAPQTP 112

XP_037222000.1 Mi -VDMQTQAETIR---TVFSDPTNFNVKHPLYSPWTLWFDSPQTKGRNMPQTPISAFPQTP 89

XP_041300350.1 Sd -VDMQAQADDIR---TVFSHPTSFNVKHPLYSPWTLWFDSPATKGRNLPQTPISSFPQTP 94

XP_007763399.1 Cp -IDMQAQADEIR---TVFSDPTNFNVKHPLYSPWTLWFDSPATKGRNLPQTPISSFPQTP 102

XP_025600987.1 Tw SSGAAGGDAEVR---TVLHDAQNFNVKHPLYNTWTLWFDNPSAKGTSAA----------- 78

XP_029738848.1 Sg SSNTTGNANEYR---TVFQDASNFNVKHPLYNSWTLWFDNPSQKGMASA----------- 92

XP_014653600.1 Ma ASSSDVNAAEYR---TVFQDATNFNVKHPLYNSWTLWFDNPSQKGMASA----------- 86

XP_007406414.1 Mlp --------NALI---TVFSSQTEFNVKHPLYSTWTLWFDNASKND--------------- 34

XP_014568616.1 Mo TAADAKADKAVK---TVFDDKLHFNVVHPLNSTWTLWFDNASKQD--------------- 76

*

** G**

Human eIF4E --------KSKTWQANLRLISKFDTVEDFWALYNHIQLSSNLMPGCDYSLFKDGIEPMWE 103

Yeast eIF4E --------KSESWSDLLRPVTSFQTVEEFWAIIQNIPEPHELPLKSDYHVFRNDVRPEWE 105

NP_594228.1 Spo ---------GLEWNELQKNIITFNSVEEFWGIHNNINPASSLPIKSDYSFFREGVRPEWE 105

NP_595451.1 Spo ---------GLEWSDLLKEIISFKTVEEFWGIFKTISKASMLPAKSDYSYFLKGIRPEWE 131

XP_047779270.1 Rr LPQTPSVA-AAGWMEDIKRVIGFDSVEEFWGLYNNIIKPSELPPKANYYLFKESIIPAWE 153

XP_009541196.1 Hi LPQTPGAAAAMGWMEDIKRVITFDSVEEFWGLHNNIVPPSQLPPKANYYLFKEGIIPAWE 173

XP_007265467.1 Fm LPQTPGAAAAMGWMEDIKRVINFDSVEEFWGLYNNIVPPSQLPQKANYYLFKEGIIPAWE 172

XP_037222000.1 Mi VAQTPGVAAAQGWMEDIKRVISFDSVEEFWGLYNNIVPPSQLPQKANYYLFKEGIIPAWE 149

XP_041300350.1 Sd VPQTPSAAAAQGWMEDIKRVVSFDSVEEFWGLYNHIVPPSQLPQKANYYLFKEGIIPAWE 154

XP_007763399.1 Cp LPQTPGNVAALGWMEDIKRVITFDSVEEFWGLYNHIVAPSHLPQKANYYLFKEGIIPAWE 162

XP_025600987.1 Tw ------KGGKDSWGEEMNKVVSFDSVEEFWGLYNNIIPPSELPQKANYYLFKEGIQPAWE 132

XP_029738848.1 Sg ------RGTKDSWGDDMNKVVDFDSVEEFWGLYNNVVPPSELPQKANYYLFKQGVKPAWE 146

XP_014653600.1 Ma ------RGTKESWGDDMNKVVDFDSVEEFWGLYQNIVPPSELPQKANYYLFKQGVKPAWE 140

XP_007406414.1 Mlp --------KAKNWDELIQRVMEVESVEEFWGLYHNIVPPSLIHIGSNYYLFKEGIKPAWE 86

XP_014568616.1 Mo --------KSRSWEDSLQQVMEINTVEEFWGLYNNIVPPSHIAISSNYYLFRKGIKPAWE 128

**P**

Human eIF4E DEKNKRGGRWLITLNKQQRRSDLDRFWLETLLCLIGESFDDY-------------SDDVC 150

Yeast eIF4E DEANAKGGKWSFQLR-GKG-ADIDELWLRTLLAVIGETIDED-------------DSQIN 150

NP_594228.1 Spo DVHNKTGGKWAFQNK-GRGGNALDEMWLTTVLAAIGETLDPT-------------GQEVM 151

NP_595451.1 Spo DPQNMNGGKWAYQSK-HKG-SNLDELWLYMVLAAIGETLDPT-------------GKEVT 176

XP_047779270.1 Rr DEANKNGGKWSIQLPKDKNRHKIDDMWLNTMLAAIGETFDPALTEAESE--DSAPQSLIT 211

XP_009541196.1 Hi DEANKNGGKWSIQLPKDKNRNHVDKMWLYTMLAAIGETFDPYLTNPDTP---AGTPSLVT 230

XP_007265467.1 Fm DEANKDGGKWSIQLPKDKNRGNVDKMWLYTMLAAIGETFDSSGAST--D--ELAPQSLVT 228

XP_037222000.1 Mi DEANKNGGKWSIQLPKDKNRGNVDKMWLYTMLAAIGETFDPSLTSADPA--GSPPSSLIT 207

XP_041300350.1 Sd DEANKNGGKWSIQLPKEKNRSQVDKMWLYTMLAAIGETFDPHLTSGEAT--EPNTQSLIT 212

XP_007763399.1 Cp DDANKNGGKWSIQLPKDKNRSNIDKMWLFTMLAAIGETFDPYLTSGEAT--EPHPQSLVT 220

XP_025600987.1 Tw DAANTNGGKWSIQLPREKSRADIDRLWLNTMLSAIGETLESPYDSSSPA--ASNTAEAIT 190

XP_029738848.1 Sg DPANTNGGKWSIQLPRDKTRAQIDRLWLYTMLAAIGETLEAPFPNGTPPPSSSPDDELIT 206

XP_014653600.1 Ma DPANTNGGKWSIQLPRDKTRAAIDRLWLFTMLAAIGETLEAPFPDGVPPPSSSPQDELVT 200

XP_007406414.1 Mlp DPSNHKGGKWSVQLPRDKNRETIDKWWLYTMLAAIGETFETPYTSNGKSPSEMTFTDEVT 146

XP_014568616.1 Mo DPANAKGGKWAVQLPRDKTAGNVDNFWLYTMLAAIGETFETPFDGSTAGT-SAEVREEVT 187

**P P m**

Human eIF4E GAVVNVRAKGDKIAIWTTECEN---------REAVTHIGRVYKE-RLGLPPKI------- 193

Yeast eIF4E GVVLSIRKGGNKFALWTKSE-D---------KEPLLRIGGKFKQ-VLKLTDDG------- 192

NP_594228.1 Spo GVVINMRKGFYRLAVWTKSCNN---------REVLMEIGTRFKQ-VLNLPRSE------- 94

NP_595451.1 Spo GVVCNMRKGFYRIAVWTRNCND---------KDVLEKIGLRFKE-VLGISDKE------- 219

XP_047779270.1 Rr GVIVSTRPQFYRISIWTRIAPGPD---DEELRKRIESVGKHFKTAVLGFPESQKLA---G 265

XP_009541196.1 Hi GVIVSTRPQFYRLSIWTRLAPGNSGADDEALRERIEAVGKHFKIGVLGYTDAQKLA---G 287

XP_007265467.1 Fm GVIVSTRPQFYRISIWTRLAPSNQTD-DEKLRKRIETVGKHFKTQVLGYADGAKLA---G 284

XP_037222000.1 Mi GVIVSTRPQFYRLSIWTRLAPSGS--EDDKLRERIEGVGKHFKTSVLGYADHAKLA---G 262

XP_041300350.1 Sd GVIVSTRPQFYRLSIWTRLAPSGD---EDKLRERIETVGRHFKINVLGYPETQKLA---G 266

XP_007763399.1 Cp GVIVSTRPQFYRLSIWTRLAPTGQGDEDDKLRERIEAMGRNFKTSVLGYSESQKLQ---G 277

XP_025600987.1 Tw GIIMSARPNLYRIAIWTRTADESA---SGELDPALLNIGKHFKVGILGYQLNQQVGG--G 245

XP_029738848.1 Sg GVIMSARANFYRIAIWTRKAEDT-----DELAAKLLDIGKQFKVNVLGYDLDAKIG---Q 258

XP_014653600.1 Ma GVIMSARANFYRIAIWTRKAEDT-----EELAAKLLDIGKQFKVNVLGYDIDAKIG---A 252

XP_007406414.1 Mlp GVIVSSRKAFYRISIWTRSSE--T-------KALAENIGRHFKYGVLGMPEGKKIASDGH 197

XP_014568616.1 Mo GVIISSRKVFWRINVWTKTATDSD-------KPRLEVIGKHIKYGILGVPVGVKLAAAGA 240

Human eIF4E ----VIGYQSHADTATKSGSTTKNRFVV- 217

Yeast eIF4E ----HLEFFPHSSANGRHPQPS----ITL 213

NP_594228.1 Spo ----TIEFSAHEDSSKSG-STRAKTRMSV 218

NP_595451.1 Spo ----TIEYSAHEDSSKAG-SMRAKTRMSL 243

XP_047779270.1 Rr PLATEVEFLSHKDSEKKGKQSKKPMTV-- 292

XP_009541196.1 Hi PLATEVEFLSHKDSEKKGKTAKKMIV--- 313

XP_007265467.1 Fm PLATEVEFLSHKDSEKKGKSKKIVV---- 309

XP_037222000.1 Mi PLSTEVEFLSHKDSEKKGGKSRKIVV--- 288

XP_041300350.1 Sd PLATEVEFLSHKDSEKKGKAAKKLVI--- 292

XP_007763399.1 Cp PLATEVEFLSHKDSEKKGKAAKKIVI--- 303

XP_025600987.1 Tw GMQSDVEFQSHKDSERKGKGRKFVV---- 270

XP_029738848.1 Sg GLTSDVEFQSHKESEKKKGKKTVV----- 282

XP_014653600.1 Ma GLTSDVEFQSHKESEKKKGKKTVV----- 276

XP_007406414.1 Mlp RLASDCEFQSHADSMKRK----------- 215

XP_014568616.1 Mo TVGTDVEFESHKDSMSGSRSNANKWKV-- 267

**Supplementary Fig. S13.** **Sequence comparison of human eIF4E and selected Class I orthologs from the *phylum* *Basidiomycota*.** Full-length proteins were compared.eIF4E from the ascomycetes *S. cerevisiae*(yeast) and *S. pombe* (Spo) (27,28) were also included to identify the reported phosphorylated residues (14). Residues involved in the cap recognition (9-12) are indicated as follows: , W binding the guanine by  –  interactions; *G*, residue recognizing the guanine ring; *P*, residues interacting with the phosphate groups; *m*, W recognizing the cap methyl group. *Asterisks* indicate W43 and W56 of the human protein used to classify the eIF4E-family members into three classes (13). Residues identical to human eIF4E are shaded in black boxes. Conservative changes are in grey boxes. Purple boxes highlight conservation of phosphorylated amino acids S2, S15, and S28 of *S. cerevisiae* eIF4E (14), and S209 of human/mouse eIF4E (15,16). Phosphorylation of *S. cerevisiae* S28 was demonstrated to increase eIF4G affinity (18). Phospho-mimicking E or D residues in the phosphorylatable positions (18,19) are also highlighted in gray boxes. Gaps are represented by dashes. Human eIF4E (acc. numb.M15353) (20); *S. cerevisiae* eIF4E (acc. numb. NP_014502.1) (25,26); *S. pombe* (Spo) (27,28) eIF4E; *Basidiomycota* species: Rr, *Rhodofomes roseus*; Hi, *Heterobasidion irregulare*; Fm, *Fomitiporia mediterranea*; Mi, *Mycena indigotica*; Sd, *Suillus discolor*; Cp, *Coniophora puteana*; Tw, *Tilletiopsis washingtonensis*; Sg, *Sporisorium graminicola*; Ma, *Moesziomyces antarcticus*; Mlp, *Melampsora larici-populina*; Mo, *Mixia osmundae*.

Human 4EHP ----------------------------------------------------MNNKFD-- 6

Yeast eIF4E MS---------------------------------------------------------- 0

XP_047799979.1 Ps MSSPNQSNQNSAMNNQNLPKNLSLTTANSNPLHQTTPTLGLFTNSQSLASPNLLGFNKSN 60

XP_031863710.1 Ks ------------------------------------------------------------ 0

XP_019014281.1 Kp ------------------------------------------------------------ 0

XP_019006202.1 Km ------------------------------------------------------------ 0

XP_009268741.1 Wi MT---------------------------------------------------------- 0

XP_006461928.1 Abi ----------------------------------------------------MASYFNSN 8

XP_007327623.1 Abu ----------------------------------------------------MASYFNSN 8

XP_008038323.1 Tv ----------------------------------------------------MAGYFS-N 7

XP_041171213.1 Sp ----------------------------------------------------MAGYFS-N 7

XP_001831439.1 Cc MS---------------------------------------------------------N 3

XP_007770843.1 Cp MS-----------------------------------------------------YFS-N 6

XP_047747127.1 Pc ----------------------------------------------------MASYFS-N 7

Human 4EHP -------ALKD------------DDSGDHD-------QN------------EENSTQKDG 28

Yeast eIF4E ----------------------------------------------VEEVSKKFEENVS- 15

XP_047799979.1 Ps FQTFARVGNSQP---------------------PLP---------------GLLSPNST- 83

XP_031863710.1 Ks ----------------------------------MAQGTAPSRRTTSHSGSARLSLSVG- 25

XP_019014281.1 Kp ----------------------------------MAQGNVPTKRTTSLSGSARLSLSVG- 25

XP_019006202.1 Km ----------------------------------MAQGTVPTRRTTSLSGSARLSLSVG- 25

XP_009268741.1 Wi ------------------------------------------------------------ 0

XP_006461928.1 Abi HATSRFLASNTP-------------------AAPAPNPNTPKSR---PPASKLFSTSLH- 45

XP_007327623.1 Abu HATSRFLASNTP-------------------PAPAPNPNTPKSR---PPASKLFSTSLH- 45

XP_008038323.1 Tv HSQSRFLANSTPNAATTTTTTTTAQN-----PPPVTPPVQPRSR---VTSSKHFSTFVS- 58

XP_041171213.1 Sp HSQFQSRFATAASSTTTTTT--------TTATTTPAISVETRPR---PPTSRHFSTSVI- 55

XP_001831439.1 Cc --------------------NSPNNSAQTTTGAPTTSANNGKNR---VSSSKHFSTSVS- 39

XP_007770843.1 Cp HSQSTRHSAIPSD-------------PQHP-SAP---SLAPRSR---APSSKHFSTSLQ- 45

XP_047747127.1 Pc HSASRFLANSTTTPATTSTALSPSNSSAQP-TAP--ATTPSRQR---IPSSKHFSTSVN- 60

*

Human 4EHP EKEKTERDKNQ-SSSKRKAVVPGPAEHPLQY-NYTF-WYS-RRTP----GRP-------- 72

Yeast eIF4E -----VDDTTATPKTVLSDSAHFDVKHPLNT-KWTL-WYTKPA-------VD-------- 53

XP_047799979.1 Ps -------TKKPQPTVRNQPTAAPVTNHPISRSGFADKETINQENEDQSITQPE-DNLIES 135

XP_031863710.1 Ks -------------KATAAAPSPASGRHPLRQ-DWSISY-VHRPP-----AA--------- 56

XP_019014281.1 Kp -------------KSAQNTSNTASGRHPLRQ-DWSISY-VHRPP-----GA--------- 56

XP_019006202.1 Km -------------KTNNNAPSPASGRHPLRQ-DWSISY-VHRPP-----GA--------- 56

XP_009268741.1 Wi --------------------------LKL-STSWTV-WGI-HRPP----SS--------- 20

XP_006461928.1 Abi QPDRAP--------AALVTAVPPSAVHPLRN-TWVF-YFRQQRSP----GN--------- 82

XP_007327623.1 Abu QPDRAP--------ATLVAAVPPSAVHPLRN-TWVF-YFRQQRSP----GN--------- 82

XP_008038323.1 Tv NAPGDEKAQGKDKAAGNGAANGSLSVHPLRNTSWVF-WFRQQRAP----GN--------- 104

XP_041171213.1 Sp HLPHEDRDRGA-------DVPAPLSVHPLRN-TWVF-WFRQQRAP----GN--------- 93

XP_001831439.1 Cc ALNNGERSKSKDSSENNS-VSSTPKVHPLRN-TWVF-WFRQQRAP----GN--------- 83

XP_007770843.1 Cp TSLE-EKSQG--KDKPIT-PSISGGVHPLRH-TWVF-WFRQQRAP----GN--------- 86

XP_047747127.1 Pc VSQP-VASKDPTSSSTTS-SASASQVHPLRN-TWVF-WFRQQRAP----GN--------- 103

*

****

Human 4EHP -------TSSQSYEQNIKQIGTFASVEQFWRFYSHMVRPGDLTGH--------------- 110

Yeast eIF4E --------KSESWSDLLRPVTSFQTVEEFWAIIQNIPEPHELPLK--------------- 90

XP_047799979.1 Ps KDELTDSKKNREYGKAMRRLGSCDTVEQFFSLYLHIKRPSQHLPI--------------- 180

XP_031863710.1 Ks ---------KVDYEKEIRRVATFGSIESFLHLYSHLTPPNELPAV--------------- 92

XP_019014281.1 Kp ---------KVEYEKEIRKVATFGSIESFLHLYSHITPPNELPPV--------------- 92

XP_019006202.1 Km ---------KVEYEKEIRKVATFGSIESFLHLYSHITPPNELPPV--------------- 92

XP_009268741.1 Wi --------KIIDYQKDLIKIAGFDSLPAFWNTYAYLKPPSTLPVV--------------- 57

XP_006461928.1 Abi --------KNVNYEEGIKKISAFSSVESFWSLWTHLASPSNLQPT--------------- 119

XP_007327623.1 Abu --------KNVNYEEGIKKISAFSSVESFWSLWTHLASPSNLQPT--------------- 119

XP_008038323.1 Tv --------KITNYEEGIKKISAFSSVESFWSLWTHVHQPSSLLPT--------------- 141

XP_041171213.1 Sp --------KITNYEEGIKKIASFSSVESFWALQTHLSAPSALVPT--------------- 130

XP_001831439.1 Cc --------KVIDYEEGIKKIAAFSSVESFWSLWTHLSPPSGLQPT--------------- 120

XP_007770843.1 Cp --------KITNYEEGIKKIASFSSVESFWSLWTHLYSPSALLPT--------------- 123

XP_047747127.1 Pc --------KIISYEEGIKKIAAFSSVESFWSLWTHLTPPSSLQPT--------------- 140

**G**  **P**

Human 4EHP --------SDFHLFKEGI-KP-MWEDDANKNGGKWIIRLRKGL--ASRCWENLILAMLGE 158

Yeast eIF4E --------SDYHVFRNDV-RP-EWEDEANAKGGKWSFQLRGKGADIDELWLRTLLAVIGE 140

XP_047799979.1 Ps --------SDLHIFGDSI-KP-AWEDPENVGGVKWKIRLKKGL--ANRLWETLIMSLVEG 228

XP_031863710.1 Ks --------TDVLVFVSRIGRPGVWEEM--RDGGRFTIRLVHPI--TPLLYESLLLALIGD 140

XP_019014281.1 Kp --------TDILVFVSRIGRPGVWEEM--RDGGKFTIRLVHPI--TPLLFENLLLALIGD 140

XP_019006202.1 Km --------TDILVFVSRIGRPGVWEEM--RDGGKFTIRLVHPI--TPLLFENLLLALIGD 140

XP_009268741.1 Wi --------TDYQLFRSGV-RP-VWEDPENIKGGKWILRLRKGI--VDQLWEDLLLSIIGC 105

XP_006461928.1 Abi --------TDYLLFHTGVRRP-VWEDHLNISGGKWIIRLRKGV--ADRLWEDLVLAIIGD 168

XP_007327623.1 Abu --------TDYLLFHTGVRRP-VWEDHLNISGGKWIIRLRKGV--ADRLWEDLVLAIIGD 168

XP_008038323.1 Tv --------TDYLLFHSGIRRP-VWEDPLNLSGGKWIIRLRKGV--ADRLWEDLVLAVIGD 190

XP_041171213.1 Sp --------TDYLLFHTGVRRP-VWEDPLNRAGGKWIVRLRKGV--ADRVWEDLVMGVVGD 179

XP_001831439.1 Cc --------TDYLLFHSNVRRP-VWEDPLNLAGGKWIIRLRKGV--ADRLWEDLVLAVIGD 169

XP_007770843.1 Cp --------TDYLLFHSGIRRP-VWEDPLNIDGGKWIIRLKKGV--ADHIWEDLVLAIIGD 172

XP_047747127.1 Pc --------TDYLLFHAGIRRP-VWEDPLNITGGKWIIRLKKGI--ADRFWEDLVLAIIGD 189

**P P m**

Human 4EHP QFM-------------------------------VGEEICGAVVSVRFQEDIISIWNKTA 187

Yeast eIF4E TID------------------------------EDDSQINGVVLSIRKGGNKFALWTKSE 170

XP_047799979.1 Ps GLEKLIQNDNNSSSSGED--DDQDDQDEGEEGWEKRREICGVVLSIRRDEDILVVWHKTG 286

XP_018270336.1 Rg RIG--------------------------GDDERVGDKVNGVVLSVRRDEDILSLWVAPS 189

XP_031863710.1 Ks QFE-------------------------------ESDNVVGCVLSVRQAEDILSVWVEEE 169

XP_019014281.1 Kp QFD-------------------------------ESDNVVGCVLSVRQTEDILSVWVEEE 169

XP_019006202.1 Km QFD-------------------------------ESDNVVGCVLSVRQAEDILSVWVEEE 169

XP_009268741.1 Wi NMV------------------------------DDINDLCGAVVSIRAAEDIISVWIRRE 135

XP_006461928.1 Abi QFDQY-------------------------DSSAAYNEICGCTISVRQNEDIISLWNRYE 203

XP_007327623.1 Abu QFDQCGNGPVP----------TNGENEEEDDSSAAYNEICGCTISVRQNEDIISLWNRYE 218

XP_008038323.1 Tv QFDGVDDGEGSQPDA----------AALEGVPPGEWPEICGCTISVRQNEDIISLWNRSD 240

XP_041171213.1 Sp MFDECGTQG--------------------EGEEGVWPEICGCTISVRQSEDIVSLWNRVD 219

XP_001831439.1 Cc LFDDCRSTTSSPTAA-K------ADGGENSEDENQYPEICGCTISVRQSEDIISLWNRVE 222

XP_007770843.1 Cp QFEDCRTVTASSET----------TNGDSRSTPSEWPEICGCTISVRQSEDIVTLWNRVD 222

XP_047747127.1 Pc QFDNCRSKVEDSPKG-KSGVSATGKSEDGSEGGSEWPEICGCTLSVRQSEDIVTVWNRVD 248

Human 4EHP S-----DQATTARIRDTLRRVLNLPPNT-IMEYKTHTDSIKMPGR--LGPQRLL-----F 234

Yeast eIF4E D-----K-EPLLRIGGKFKQVLKLTDDG-HLEFFPHSSANGRHPQPSITL---------- 213

XP_047799979.1 Ps TPGSC-DVKKAKQVKLSLQTVLQLPLNC-HLVYKLNVDCLP--TN----VDLTTIVNNIQ- 338

XP_031863710.1 Ks S-----DGVRNGALKEKILSLLSLPPTT-TCDYRANRALLEVAAKPPFNQTNSGNPAALP 223

XP_019014281.1 Kp S-----DSVRSGALKEKILTLLSLPSTT-SCEYRANRIFLEATSKPAFNNVNPINAINEH 223

XP_019006202.1 Km S-----DSVRSGALK--------------------------------------------- 179

XP_009268741.1 Wi ------DPDLVNSVRDSIYKSLNQSPSTLEMQFKSNSESLQDKTP--QLNTPK------- 180

XP_006461928.1 Abi A-----DFKVRERIRDTLRRVLNLPPTT-TLEYKTNNDSMLDQSS--FRTTGLH-----D 250

XP_007327623.1 Abu A-----DFKVRERIRDTLRRVLNLPPTT-TLEYKTNNDSMLDQSS--FRTTGLH-----D 265

XP_008038323.1 Tv G-----NPKSKEKIKETIRRVLNLPPAT-IMEYKSNNDSMQDKSS--FRVNQTD------ 286

XP_041171213.1 Sp G-----DAKVREKIRDTLRVVLNLPPST-VMEYKSNNDSMQDKSS--FRNSAIE------ 265

XP_001831439.1 Cc A-----NRQVREKIRDTIRRVLNLPPST-IMEYKTNNDSMQDKSS--FRNSAID------ 268

XP_007770843.1 Cp A-----DVKVREKIRDTLRTVLNLPPST-IMEYKSNNDSMQDKSS--FRNSAID------ 268

XP_047747127.1 Pc G-----DPKLREQIRDTLRKVLNLPPST-IMEYKSNNDSMQDKSS--FRRTAID------ 294

Human 4EHP QNLWKPRLNVP------------------------------------------------- 245

Yeast eIF4E ------------------------------------------------------------ 213

XP_047799979.1 Ps QNPYNNNNNHNHHHHNNQHYHH-------HGQNKHNNYRQQNHTNCHS------------//432

XP_031863710.1 Ks ETPNSHNHNNP--HHNHNHEHHPRVHHV------HQH---DR—PHRERERERGGDRDRE-//316

XP_019014281.1 Kp --QNEHHSNN-----NNHHQQHPRQHHS------HHNYIHDKHNVDRSDRGERGER----//309

XP_019006202.1 Km ------------------------------------------------------------ 179

XP_009268741.1 Wi ------------------------------------------------------------ 180

XP_006461928.1 Abi RTPHT------------------------------------------------------- 255

XP_007327623.1 Abu RTPHT------------------------------------------------------- 270

XP_008038323.1 Tv RTPLS------------------------------------------------------- 291

XP_041171213.1 Sp RMPITAAVS--------------------------------------------------- 274

XP_001831439.1 Cc RATLS------------------------------------------------------- 273

XP_007770843.1 Cp RTPLA------------------------------------------------------- 273

XP_047747127.1 Pc KTPMSPAS---------------------------------------------------- 302

**Supplementary Fig. S14.** **Sequence comparison of human 4EHP with selected Class II orthologs from the *phylum* *Basidiomycota*.** Full-length proteins were compared.eIF4E from the ascomycete *S. cerevisiae*(yeast) was also included to identify the reported phosphorylated residues (14). Residues involved in the cap recognition (9-12) are indicated as follows: , W binding the guanine by  –  interactions; *G*, residue recognizing the guanine ring; *P*, residues interacting with the phosphate groups; *m*, W recognizing the cap methyl group. *Asterisks* indicate W43 and W56 of the human protein used to classify the eIF4E-family members into three classes (13). Residues identical to human 4EHP are shaded in black boxes. Conservative changes are in grey boxes. Purple boxes highlight conservation of phosphorylated amino acids S2, S15, and S28 of *S. cerevisiae* eIF4E (14), and S209 of human/mouse eIF4E (15,16). Phosphorylation of *S. cerevisiae* S28 was demonstrated to increase eIF4G affinity (18). Phospho-mimicking E or D residues in the phosphorylatable positions (18,19) are also highlighted in gray boxes. Gaps are represented by dashes. // indicates that some amino acids are not included. Human human 4EHP (acc. numb. AF047695) (29); *S. cerevisiae* eIF4E (acc. numb. NP_014502.1) (25,26). *Basidiomycota* species: Ps, *Puccinia striiformis tritici*; Ks, *Kwoniella shandongensis*; Kp, *Kwoniella pini*; Km, *Kwoniella mangroviensis*; Wi, *Wallemia ichthyophaga*; Abi, *Agaricus bisporus bisporus*; Abu, *Agaricus bisporus* *burnettii*; Tv, *Trametes versicolor*; Sp, *Suillus paluster*; Cc, *Coprinopsis cinerea* *okayama*; Cp, *Coniophora puteana*; Pc, *Psilocybe cubensis*.

*

Human eIF4E KHPLQNRWALWFFKN---------------------------------------------DKS 53

XP_046039764.1 Ff LHPLQHTWTLYHDTGRPITDQDMFVSQSGTTLS-----------MKL---GRTIGDEKKNKIE 387

XP_047805662.1 Pst IHPLQHSWTLYFDTRLTTKRSST-------------------------------SGGGGTTGG 163

XP_025350281.1 Pg THPLQHEWTLYFDSKEGGPSSVSSSPLGAATRAGNSPTKAT-S-PSLESSAAPSGPSKGASNS 388

XP_025373151.1 Cg DHPLQHKWTLYFDSKTFNPSSQASTPATGHTADGPGTPGAA-LPGASQNPISPLPPTPNTANI 331

XP_001729563.1 Mg KHPLENEWTLYYDLQKLHGS----------------------------------------ASS 153

XP_017990509.1 Mp KHPLEHAWTLYYDLQRHHGQ----------------------------------------SSS 240

XP_029740494.1 Sg TYPLQHEWTLFFDSRATAPPT----------------PGFAPSPADVASCSSSSVPST-PTSL 343

XP_012191249.1 Ph THPLQHEWTLFFDTRSAAAPST---------------PTLAPPASPL------VQPSTPTQSA 263

XP_018270971.1 Rg DLPLSASWTLFFSDTSGAAKSNSAA-----------------------------------ATK 215

XP_019032290.1 Cw VHPLDRPWTLYFSDSSEKAESQ--------------------------------------QSA 892

XP_018997288.1 Ca VHPLDRLWTLYFSDSSEKAQSQ--------------------------------------QSA 900

XP_018263647.1 Kd IYPLNNSWTLYYSNTSHQRKVS--A-------------------------PIPVPALGFHPNA 649

XP_019002155.1 Km FHPLTHSWTMYFSNTAHQHQRKVSMPA-----------------------LSPLNPLSSHPNA 512

*

****

Human eIF4E KTWQANLRLISKFDTVEDFWALYNHI--------QL-----------------SSNLMPG 88

XP_046039764.1 Ff MEFESRLLILGDFRTVEDFGKTFGQTPDQ---KRNY-----------------PSQMNSG 427

XP_047805662.1 Pst QAYEAGLQPIGTFKSVEQFCGFFNWT--------VL-----------------PSQMEMN 198

XP_025350281.1 Pg ETWEAALKKVGEYRTVESFMSVFGTI--------KR-----------------PSTLERN 423

XP_025373151.1 Cg QSWEDTLKMLGVYKTVEGFMNVFATL--------RR-----------------PSQLERN 366

XP_001729563.1 Mg DQYEATLKRVGHFTTLESFFDTFATL--------HR-----------------PSRLEKN 188

XP_017990509.1 Mp DQYEATLKCVGEFTTLESFFDTFATL--------HR-----------------PSRLEKN 275

XP_029740494.1 Sg SAWEANLRTIGSYSHVSTFLSCFSKL--------HR-----------------PSQLERH 378

XP_012191249.1 Ph SSWEANLRCIGAYTTVEAFLSCFAKL--------RR-----------------PSQLERH 298

XP_018270971.1 Rg EAYHEGINPIFSAKTVPELCGQYKAFKQAPKSKRAKAGDPE-----------TLGLTRPG 264

XP_019032290.1 Cw REYDSGLVKVFHAACIEDLFGSWKALRRAIAYSKGREIEPEGRPL-EGGGGLGMWLMGDD 951

XP_018997288.1 Ca REYDSGLVKVFHAACIEDLFGSWKALRRAIANSKGREIEPEGRPL-EGGGGLGMWLMGDD 959

XP_018263647.1 Kd VDYSSHLFTIFSANNLEDLFGSWKALRRSIARSKGRNIEPLGDSMMKGGPGLGTHFFPDE 709

XP_019002155.1 Km SDYSSHLVTLFKADNLEDLFGGWKALRRSIAKSKRREIEPIGDSVQKGGSGLGTHLFQEE 572

**G P**

Human eIF4E CDYSLFKDGIEPMWEDEKNKRGGRWLITLNKQQ--------RRSDLDRFWLETLLCLIGE 140

XP_046039764.1 Ff SNLHLFKDGIRPMYEDPANKQGGRWTLVLQN----------LPGLLDRVWQNLVCGMIGE 477

XP_047805662.1 Pst SSVQIFKSHIKPMWEDPANSKGGKWTITIKSSS--------NLALLDKLWTYLVLGLVGE 250

XP_025350281.1 Pg ANYHLFKDGIKPMWEDPANARGGKWVLTFDRK-------LSNPALVDRSWIWLVLALIGE 476

XP_025373151.1 Cg CNYHLFKSGIKPMWEDPANASGGKWVLTLRGT---------SGALLDRSWMWLVLALIGE 417

XP_001729563.1 Mg SNYHFFKNGVKPLWEDPENASGGRWVITLRDRGQTAGSRAGHEALLDRSWMWLVLALIGE 248

XP_017990509.1 Mp ANYHLFKSGVKPMWEDPANAEGGRWVITLRDRAQTAGSRAAHEALVDRSWMWLVLALIGE 335

XP_029740494.1 Sg SSYHVFKDGIKPMWEDPRNADGGKWVITFRQR---------NAALVDRSWLWLVLGLIGE 429

XP_012191249.1 Ph SSYHCFKDGIKPMWEDARNANGGKWTLTFRQR---------HPALVDRSWLWLVLGLIGE 349

XP_018270971.1 Rg MNLHFFRAGINPTWEDPYNEKGGRITISPS------------AALFDNIYERLVFLLAGA 312

XP_019032290.1 Cw TNFHLFADGIKPMWEDPMCAKGGKLMMAGD------------AKKMDDVFLELCLLLVGG 999

XP_018997288.1 Ca TNFHLFADGTKPMWEDPMCAKGGKLMMAGD------------AKKMDDVFLELCLLLVGG 1007

XP_018263647.1 Kd TNFHFFKSGIKPMWEDKMCQKGGKIMIAGE------------AVVIDNLFLEFVLLLISG 757

XP_019002155.1 Km TNFHMFKSHIRPMWEDKYCQKGGKIMIAGE------------ASAMDDLFLELIFLLISG 620

**P P m**

Human_eIF4E SFDD------------------------YSDDVCGAVVNVRAKGDKIAIWTTE-CEN--- 172

XP_046039764.1 Ff QIDE-------------------------GDDICGAVISIRPKVQRIQVWVRN-KNN--- 508

XP_047805662.1 Pst QVENAHHS----------------KGEEEDHFVCGAIVATRPRGNRIQIWVKE-KDN--- 290

XP_025350281.1 Pg ELDA-------------------------DDEVTGAVVSTRPKFDRIALWVRG-KQD--- 507

XP_025373151.1 Cg ELDD-------------------------ANHITGAVVSTRSKGDRITLWIRN-KDD--- 448

XP_001729563.1 Mg TLEE-------------------------NDLVTGAVCSLRGKGDRITVWTRR-KEP--- 279

XP_017990509.1 Mp TLDD-------------------------DGLVTGAVCSLRGKGDRIALWTRA-KEP--- 366

XP_029740494.1 Sg ELDG-------------------------ADECCGAVCSVKPRGDRIALWIKKGVSG--- 461

XP_012191249.1 Ph EMDE-------------------------GDEVCGAVCSVKPRGDRISLWVRD-RSD--- 380

XP_018270971.1 Rg ALELGTSDLLSTEGPSPGSKRPPTPGPPQEGQINGVVASRRARGDRIEIWLGG-REKKTA 371

XP_019032290.1 Cw NLEVDMPP---------------L----TKPSVCGAIVSRRKTTTRIEVWLGG-R-DV-- 1036

XP_018997288.1 Ca NLEVDTPP---------------L----TKPSVCGAIISRRKTTTRIEVWLGG-R-DV-- 1044

XP_018263647.1 Kd EIDETIPP---------------P--AGSSSTICGVVLSRRK-LTRLELWLGG-K-TS-- 795

XP_019002155.1 Km DLEEEVLP---------------P--PGSSSTICGLVLSRRK-LTRIELWLGG-AHVI-- 659

Human eIF4E --REAVTHIGRVYKERLGLPPK--IV--IGYQSHADTATK-SGSTTKNRFVV-------- 217

XP_046039764.1 Ff --FDTVEQIGQTLLRVLDIAEP-NVVTSFEFAGNLGPPASDRPHLQIGKV----IKNGP-//628

XP_047805662.1 Pst --VDKINGLGKRLINLLEINEHSGVS--VDFSGHTGGTHGSSRFISIQPV-AGSGGAGGG//581

XP_025350281.1 Pg --VKRVNEIGRKLIDLLQVESEPGVA--LEFSSNSTGSHSRSAHYGSQIF-LGFSNG-A-//638

XP_025373151.1 Cg --VDLVNRLGKKLVHLLDIEHEPGVS--LEFTLNSGPGPANAAAQANR-Y-VHFSNA-P-//714

XP_001729563.1 Mg --VDEINSLGQRLLELLELQDEPGIQ--MDFSVNSGSKESQQQSYMR----KHLTSH-A-//377

XP_017990509.1 Mp --VDELNKLGKKLLQLLELENEPSAQ--LEFGVNFGSKDP---GYIK----QHLGGK-S-//456

XP_029740494.1 Sg --VDGCNRVGRRLVELLELEREPGVL--VEFSSHSSAAGA---------AKEGLWTL-N-//673

XP_012191249.1 Ph --VDKVNRIGKKLASLLEVENEPGVT--LEFSAHSDRSEQ---------KLEGLYSL-Q-//563

XP_018270971.1 Rg VPIEWLDRFKEVLAVELDMPEL-KS---SKYKKHF------------------------- 402

XP_019032290.1 Cw PDKRWVNDVHDKLSNF--FPQI-RV---LPYKSFHRN----------------------- 1067

XP_018997288.1 Ca PDKRWVNDVHDKLSNW--FPQI-RV---LPYKSFHRN----------------------- 1075

XP_018263647.1 Kd PDREWVGKVTRFIEER--FRGS-KV---YGFKSFGKN----------------------- 826

XP_019002155.1 Km PDKRWIEQVTRYIQMH--FKEW-RV---YPYKAFGKS----------------------- 690

**Supplementary Fig. S15.** **Sequence comparison of human eIF4E with selected F-Class VI orthologs from the *phylum* *Basidiomycota*.** The core of the proteins and part of the carboxy-terminal was compared. Residues involved in the cap recognition (9-12) are indicated as follows: , residues binding the guanine by  –  interactions; *G*, residue recognizing the guanine ring; *P*, residues interacting with the phosphate groups; *m*, Trp recognizing the cap methyl group. *Asterisks* indicate W43 and W56 of the human protein used to classify the eIF4E-family members into three classes (13). Residues identical to human eIF4E are shaded in black boxes. Conservative changes are in grey boxes. W/Y/F residues equivalent to human W56 that define F-Class VI proteins are highlighted in red. Gaps are represented by dashes. The phosphorylated amino acids S2, S15, and S28 of *S. cerevisiae* eIF4E (14), and S209 of human/mouse eIF4E (15,16) are not conserved (not shown). Human eIF4E (acc. numb.M15353) (20). // indicates that some amino acids are not included. *Basidiomycota* species: Ff, *Filobasidium floriforme*; Pst, *Puccinia striiformis tritici*; Pg, *Pseudomicrostroma glucosiphilum*; Cg, *Ceraceosorus guamensis*; Mg, *Malassezia globosa*; Mp, *Malassezia pachydermatis*; Sg, *Sporisorium graminicola*; Ph, *Pseudozyma hubeiensis*; Rg, *Rhodotorula graminis*; Cw, *Cryptococcus wingfieldii*; Ca, *Cryptococcus amylolentus*; Kd, *Kwoniella dejecticola*; Km, *Kwoniella mangroviensis*.

Human eIF4E ------------------------MATVEPETTP-----------TPNPP-TTEEEKTES 24

Yeast eIF4E MS----------------------------------VEE---VSKKFEENVSVDDTTATP 23

NP_594228.1 Spo ----------------------------MQTEQPP-------KESQTENTVSEPQEKAL- 24

NP_595451.1 Spo MADAEDSRH-----SKNEGFPNTSLITEKLDLLDLFGSP----KVKTEREGR-PARLLEG 50

XP_031025024.1 Sm -------------------------------MAP---------AEEKTAETTTESPATDI 20

XP_052967612.1 Pa ------------------MPHSKD---IDHDEDPQNVEEEQQQIQDDSTDQQVAEEEAAL 39

XP_006679878.1 Bd ------------------------------------------MAAADDETQSVEQAVEEQ 18

XP_052921665.1 Fj -----------------------------------------------------------M 1

XP_016612132.1 Sp MATTAASLSATDDSFQNGAPHPSNHNPIAESDTPR-------TSSPEPDQVS-DDESSSF 52

XP_031026772.1 Sm MSAPVTSNGVANRIVQQQSDAE-------ARKDGMH------VLRTPWHQ-AIPQDLST- 45

XP_052926586.1 Fj MA-------------------------------------------------AAPAIG--- 8

XP_006682495.1 Bd MATSLETSV-----VTTNND-------------------------------SIPDED--- 21

XP_052965882.1 Ps MHSSHNPTS-----LSNGSA-------------------------------ETTIADST- 23

*

* ****

Human eIF4E NQEVAN-P-EHYIKHPLQNRWALWFFKND---K--SKTWQANLRLISKFDTVEDFWALYN---- 77

Yeast eIF4E KTVLSD-SAHFDVKHPLNTKWTLWYTKPAVD-K--SESWSDLLRPVTSFQTVEEFWAIIQ---- 79

NP_594228.1 Spo RTVFDD-KINFNLKHPLARPWTLWFLMPPTP--G--LEWNELQKNIITFNSVEEFWGIHN---- 79

NP_595451.1 Spo LSAVNA-ETAFVKTHPLQHEWTLWFLKPPTQ--G--LEWSDLLKEIISFKTVEEFWGIFKGIFK 109

XP_031025024.1 Sm ITVFDD-PINFTHKHPLQHKWCVWFDSAQGKQN--QKNWMDNLKNLVTFDSVEDFWGIMN---- 77

XP_052967612.1 Pa VTVFED-PVNFTVKHPLQSRWTLWYLNSQRKAN--QNNWNQNLKKIITFETVEDFWGVFN---- 96

XP_006679878.1 Bd VTAFDD-IDSMTVKHPLQSAWTLWFDSPNKKSS--VKEWSSNLKEIISFDTVEDFWGVIN---- 75

XP_052921665.1 Fj LTVFDN-PSSFNVKHPLQNRWTLWFDNPQKRTG--MHNWEKSLKNLITFDTVEDFWGVYN---- 58

XP_016612132.1 Sp KTIFDD-PKNFNAKHPLQNRWTMWFDNPGKRTN--QHNWSNNLKNLITVDTVEDFWGVYN---- 109

XP_031026772.1 Sm INFFDAVYRESSQRHIIV--YTRFM-HRPPGQKIISDDYLSGVKEVGTFATIEEFWGLYS---- 102

XP_052926586.1 Fj --AWSNTKPNTLATHPLRYPWVFWFMHREAGAKI--ENYNNSIKKISTFSTVEEFWGVYN---- 64

XP_006682495.1 Bd ---TQASSHSSSEFHALHYSWVFWFMHRSPGAKI--QDYTNEIKHVCTFSTVEEFWGAFS---- 76

XP_052965882.1 Pa QPSPELIVPFPSKGHLLKSGWAFWFMHREPGSKL--QDYNSAIKRIAGFASIEEFWAVYS---- 81

**G P**

Human eIF4E HIQLSSNLMPGCDYSLFKDGIEPMWEDEKNKRGGRWLITLNKQQRRSDLDRFWLETLLCL 137

Yeast eIF4E NIPEPHELPLKSDYHVFRNDVRPEWEDEANAKGGKWSFQLRGKG--ADIDELWLRTLLAV 137

NP_594228.1 Spo NINPASSLPIKSDYSFFREGVRPEWEDVHNKTGGKWAFQNKGRGG-NALDEMWLTTVLAA 138

NP_595451.1 Spo TISKASMLPAKSDYSYFLKGIRPEWEDPQNMNGGKWAYQSKHKGS-N-LDELWLYMVLAA 163

XP_031025024.1 Sm HIMKASALPAGSNYHVFKEGIQPMWEDPQNKKGGKWVVSITKKHR-GQMDTWWLNTLMGM 136

XP_052967612.1 Pa NIIKPSQTAPGSDYHLFKEGIQPTWEDPANEQGGKWVCTIRK-KD-EDLDTKWLNTMLLC 154

XP_006679878.1 Bd NVIGALDIPLAGNYHLFRKGIEPAWEDKANSKGGKWSFAITKQRRVQDLDKFWLNTMMAV 135

XP_052921665.1 Fj NIVNSSQLAHGANYHLFKEGVRPMWEDPMNENGGKWVVPLPKGKR-DNLDDYWLHTMLAC 117

XP_016612132.1 Sp NVVKASQLTHGSNYHIFKEGVQPMWEDPHNANGGKWVVQLPKSKR-SELDQMWLFSVLAA 168

XP_031026772.1 Sm RMRRPNELPNISDIHLFRKGTRPVWED--NPKGGKWIVRLKKGL----SSRYWENLVIAV 156

XP_052926586.1 Fj RLTRPMELNNVCDFHLFKQGIRPIWED--NLNGGKWIVRLKKGL----ASRYWESLVMAM 118

XP_006682495.1 Bd HMKRPGELSNISDYHFFKKGIRPIWED--NLTGGKWIIRLKKGI----ASRYWEDLLLAI 130

XP_052965882.1 Pa HLNRPHDLPTISDYHLFKSGLRPIWED--NIKGGKWIVRLKKGL----ASRYWESLVISL 135

**P P m**

Human eIF4E IGESFDDYSDDVCGAVVNVRAKGDKIAIWTTECENREAVTHIGRVYKERLGLPPKIVIGY 197

Yeast eIF4E IGETIDEDDSQINGVVLSIRKGGNKFALWTKSE-DKEPLLRIGGKFKQVLKLTDDGHLEF 196

NP_594228.1 Spo IGETLDPTGQEVMGVVINMRKGFYRLAVWTKSCNNREVLMEIGTRFKQVLNLPRSETIEF 198

NP_595451.1 Spo IGETLDPTGKEVTGVVCNMRKGFYRIAVWTRNCNDKDVLEKIGLRFKEVLGISDKETIEY 223

XP_031025024.1 Sm IGEAFED-GAEIMGAVVSARRSYDRISLWTKTGAIQDVQERIGKQFKQIMGVDSDITLGY 195

XP_052967612.1 Pa VGAMLPD-YDQVNGVVISIRRQQDRIALWTATN-EKDACERSGRQLKRVLELPENVRIGF 212

XP_006679878.1 Bd IGEQFTY-SDEITGIVVSNRRAHDRISVWTRDAEDREKCLKIGEELKKFL--PTTEIISY 192

XP_052921665.1 Fj VGENFPD-SDEICGAVVSIRKQQDRLALWTRNALKEDETKAAGTIWKTVLQLPDSETIGY 176

XP_016612132.1 Sp IGETFPD-SDEICGIVVSIRKQQDRLSLWTKSALDEDKCRASGQHWKGVLGLGDGEKIGY 227

XP_031026772.1 Sm IGDNFGD-SSEITGAVISIRNNEDILSLWNTNASEGRIGLRIRDTMKKLLDLPGNCTMEY 215

XP_052926586.1 Fj IGDQFDV-GNEICGAVISIRHSEDILSLWNLSADEGRVNLRIRDTLKRVLNLPPNCIMEY 177

XP_006682495.1 Bd VGDQFDV-GDEICGAVVSIRHSEDIVSLWNKSADEGRINLRIRDTLKRVLSLPANCVMEY 189

XP_052965882.1 Pa IGDQFDV-GDEICGAVISIRHSEDILSLWNRNADDSRVNLKIRDTLKKVLNLPANCVMEY 194

Human eIF4E QSHADTATKSGSTTKNRFVV--- 217

Yeast eIF4E FPHSSANGRHPQPSITL------ 213

NP_594228.1 Spo SAHEDSSKSG-STRAKTRMSV-- 218

NP_595451.1 Spo SAHEDSSKAG-SMRAKTRMSL-- 243

XP_031025024.1 Sm QMHDTALKTNSSYGNDDIYQV-- 216

XP_052967612.1 Pa QAHADAAAKNSSFANDDLYTV-- 233

XP_006679878.1 Bd LSHSDPQKKKSSSFNSDKYTI-- 213

XP_052921665.1 Fj QAHSDALKKNSRYVSCDFGVHS- 198

XP_016612132.1 Sp QAHSDALRKNSSFSNQDMYTV-- 248

XP_031026772.1 Sm KAHTAALTDGSSFKNTETYR--- 235

XP_052926586.1 Fj KAHKSSVADNSSFRNTDLYL--- 197

XP_006682495.1 Bd KAHKAAVADNSSFRNTETYR--- 209

XP_052965882.1 Pa KAHKSSITDNSSFRNTEMFR--- 214

**Supplementary S16. Sequence comparison of human eIF4E with Class I and Class II orthologs of the *phylum Chytridiomycota.*** eIF4E from the ascomycetes *S. cerevisiae*(yeast) *S. cerevisiae* and *S. pombe* (Spo) were also included to identify the reported phosphorylated residues (14). Full-length proteins were compared. Residues involved in the cap recognition (9-12) are indicated as follows: , Trp binding the guanine by  –  interactions; *G*, residue recognizing the guanine ring; *P*, residues interacting with the phosphate groups; *m*, Trp recognizing the cap methyl group. *Asterisks* indicate W43 and W56 of the human protein used to classify the eIF4E-family members into three classes (13). Residues identical to human eIF4E are shaded in black boxes. Conservative changes are in grey boxes. Purple boxes highlight conservation of phosphorylated amino acids S2, S15, and S28 of *S. cerevisiae* eIF4E (14), and S209 of human/mouse eIF4E (15,16). Phosphorylation of *S. cerevisiae* S28 was demonstrated to increase eIF4G affinity (18). Phospho-mimicking E or D residues in the phosphorylatable positions (18,19) are also highlighted in gray boxes. Gaps are represented by dashes. Class I and II species are in black and red, respectively; Class II-specific residues are highlighted in red. Human eIF4E (acc. numb.M15353) (20); *S. cerevisiae* eIF4E (acc. numb. NP_014502.1) (25,26); *S. pombe* (SPo) (27,28) eIF4E.*Chytridiomycota* species: Sm, *Synchytrium microbalum*; Pa, *Polychytrium aggregatum*; Bd, *Batrachochytrium dendrobatidis*; Fj, *Fimicolochytrium jonesii*; Sp, *Spizellomyces punctatus*.

Human eIF4E --------------------------------------MATVEPE------------TTP 10

Yeast_eIF4E --------------------------------------------------MSVEEVSKKF 10

NP_594228.1 Spo --------------------------------------MQTEQPPKESQ----------- 11

NP_595451.1 Spo ---------------------MADAEDSRHSKNEGFPNTSLITEKLDLLDLFGSPKVK-- 37

XP_013239191.1 Md --------------------------------------------------MTLLCIE--- 7

XP_008074794.1 Vf ------------------------------------------------------------ 0

XP_051345132.1 Nh --------------------------------------------------MS-------- 2

XP_052904897.1 Na --------------------------------------------------MS-------- 2

XP_047770386.1 Nj --------------------------------------------------MD-------- 2

XP_051332673.1 Nm --------------------------------------------------MS-------- 2

XP_024332261.1 Vc ------------------------------------------------------------ 0

XP_014563204.1 Oc ------------------------------------------------------------ 0

XP_003073379.1 Ei ------------------------------------------------------------ 0

NP_597220.1 Ec --------------------------------------------------ME-------- 2

XP_003887769.1 Eh ------------------------------------------------------------ 0

XP_009265002.1 Er ------------------------------------------------------------ 0

XP_013237606.1 Md MVYTSSAVTSSSGSMGSSGESLFCGPNAVSNPYSFLQELGSGETSEVPSLCSSTETLSEE 60

XP_013239128.1 Md MVYTSSAVTSSSGSMGSSGESLFCGPNAVSNPYSFLQELGSGETSEVPSLCSSTETLSEE 60

XP_008074945.1 Vf --------------------------------------------------MT-------- 0

XP_051345334.1 Nh --------------------------------------------------MS-------- 2

XP_047772122.1 Nj ------------------------------------------------------------ 0

XP_051334052.1 Nm --------------------------------------------------MT-------- 0

XP_013058026.1 Np ------------------------------------------------------------ 0

XP_052905898.1 Na ------------------------------------------------------------ 0

*

* ****

Human eIF4E TPNPPTTEEEKTESNQEVANPEHYIKHPLQNRWALWFFKN--DK---SKTWQANLRLISK 65

Yeast_eIF4E EENVSVDDTTATPKTVLSDSAHFDVKHPLNTKWTLWYTKP---AVDKSESWSDLLRPVTS 67

NP_594228.1 Spo ENTVSEPQEKALR-TVFDDKINFNLKHPLARPWTLWFLMPPTP----GLEWNELQKNIIT 66

NP_595451.1 Spo TEREGRPARLLEGLSAVNAETAFVKTHPLQHEWTLWFLKPPTQ----GLEWSDLLKEIIS 93

XP_013239191.1 Md KNLDSTGSG-----TPSLTSEAAKQSYPLKYPWTMYYDTPN-KR-TTQENYNTNLQALAT 60

XP_008074794.1 Vf ------------------------MEYPTKTEWTMWYDCQA-KRNLSSENYSSCLNEIST 35

XP_051345132.1 Nh -----------------MGIEPVSQRHQLNESWTLWYDFQE-KKYVNTDNWSDNLQQLGI 44

XP_052904897.1 Na -----------------MDMENMPARHKLNEEWVLWFDYQD-KKYTNNDNWSDSLHKLGT 44

XP_047770386.1 Nj -------------------TENASSRHKLNEEWTLWYDYQE-KKYTYSSNWSENLQKLGA 42

XP_051332673.1 Nm -----------------MEVETKTPRHRLNENWTFWYDFQE-KKYVNADNWSDNLQKLGV 44

XP_024332261.1 Vc --------------------------MKLQSKWVIWQNTSE---DSDVKSWADDLKNVGE 31

XP_014563204.1 Oc --------------------------MNLESSWVLWQNGND---DSNIRSWGEDILEVGE 31

XP_003073379.1 Ei --------------------------MNLASQWVLWQNNND---ESNAKSWGDDLMVVGE 31

NP_597220.1 Ec ----------------------------LASQWVFWQNSND---ESNAKSWGDDLVAVGE 31

XP_003887769.1 Eh --------------------------MNLASQWVLWQNNND---ESNAKSWGDDLVAVGE 31

XP_009265002.1 Er --------------------------MNLASQWVLWQNNND---ESNAKSWGDDLVAVGE 31

XP_013237606.1 Md TPNLSS---ILNSLQKEKGTDALQLKTKLPYAWVFWYLRPPQGLKSTQLNFESLLKYIGT 117

XP_013239128.1 Md TPNLSS---ILNSLQKEKGTDALQLKTKLPYAWVFWYLRPPQGLKSTQLNFESLLKYIGT 117

XP_008074945.1 Vf --------------------------HPLFVPFTVKTITR-NTTKKEPFDFCNSLKKLCT 35

XP_051345334.1 Nh ------------------------PIQTLAFPLVLSSVSRTVHTKGETEDFRSKIKAEAV 38

XP_047772122.1 Nj ------------------------MPSTLSSALVLSSVFRSVNIKGDSEDFKSKIKQEAK 36

XP_051334052.1 Nm -------------------------QERLGSALILSSVFRAVNIKGDTEDFKSKIKQEAK 37

XP_013058026.1 Np -----------------------MANQKLASTLILSSVFRCLTVKGDTEDFKSKIKQEAT 37

XP_052905898.1 Na -----------------------MNSQKLASTLILSSVFRCMTVKGDTEDFKSKIKQEAI 37

**G P**

Human eIF4E FDTVEDFWALYNHIQLSSNLMPG-CDYSLFKDGIEPMWEDEKNKRGGRWLITLNKQQRR- 123

Yeast_eIF4E FQTVEEFWAIIQNIPEPHELPLK-SDYHVFRNDVRPEWEDEANAKGGKWSFQLR-GKG-- 123

NP_594228.1 Spo FNSVEEFWGIHNNINPASSLPIK-SDYSFFREGVRPEWEDVHNKTGGKWAFQNK-GRG-- 122

NP_595451.1 Spo FKTVEEFWGIFKTISKASMLPAK-SDYSYFLKGIRPEWEDPQNMNGGKWAYQSK-HKG-- 149

XP_013239191.1 Md VDYVYTACYMIQNIPDASELPSG-TSLSFFRSDIKPSWEDSHNANGGKWTCSIHKDGS-- 117

XP_008074794.1 Vf IDDLPQLLYLLDNLEKASAWPLN-SNVHFFRRGIQPLWEDKANMKGGKWVIEIPKGFGTV 94

XP_051345132.1 Nh ADNAEMFWAIIDKIGETQDLPIS-SNLHFFRKDILPMWEDQRNLEGGKWVLELPSPQLA- 102

XP_052904897.1 Na VSDIEAFWSTLKEIGDLALLPVS-SNLHFFRNGIEPMWEDPRNSAGGKWVLELPFGQNP- 102

XP_047770386.1 Nj VSDVETFWGIMKEIGDVTMLPIS-SNLHFFRTGIEPMWEDKKNAHGGKWVLEIPGGSPV- 100

XP_051332673.1 Nm APDVETFWSIVKEVGDVSSLPIS-SNIHFFRNGIAPMWEDKRNANGGKWVLELPAGVPS- 102

XP_024332261.1 Vc FTQIEEFKFFADELKEKKLDKLL-S-LKIFKSGIKPMWEDPRNMNGGRLVIDIPTASG-- 87

XP_014563204.1 Oc VSTVPEFLYMCDMIVKNGVENLC-S-MNLFKKGIKPMWEDEANVNGGRLILDIPMMTR-- 87

XP_003073379.1 Ei VSTIPEFLYLCDEISNVGVGKLC-T-MNLFKKGIKPMWEDEGNIEGGRIIMDVPVVG-K- 87

NP_597220.1 Ec VSTVPEFLYLCDEISNAGIGRLC-T-MNLFRKGIKPMWEDEANIDGGRIIMDVPVSGK-- 87

XP_003887769.1 Eh VSTVPEFLYLCDEISNVGIGKLC-T-MNLFRKGVKPMWEDEANIDGGRIIMDVPVVGR-- 87

XP_009265002.1 Er VSTIPEFLYLCDEISNAGIGKLC-T-MNLFKKGVKPMWEDEANIDGGRIIMDVPVVGR-- 87

XP_013237606.1 Md MQTLQEFWSIYLTLKRPDELSVGTTDYSFFKENIRPVWEDPANQHGGKLVIRLRK--NS- 174

XP_013239128.1 Md MQTLQEFWSIYLTLKRPDELSVGTTDYSFFKENIRPVWEDPANQHGGKLVIRLRK--NS- 174

XP_008074945.1 Vf LKSVENLLYFLNHV-NFDHIEGI-TDISIFKDGIEPLWEDKSNIKGGKWIIKLRREV-S- 91

XP_051345334.1 Nh IKNINEFLYVIRRLHKLVDIKPI-TDVCLFREGVQPMWEDPENLEGGKWIFKMRKNTAE- 96

XP_047772122.1 Nj ITTPEEFLYVIRRLLKLQDIKTI-TDLSLFKEGIEPMWEDPSNLKGGKWIVKIKRNTAE- 94

XP_051334052.1 Nm VESAEEFLYLLRRLHKLQDIKPI-TDLSLFKDGIEPMWEDPCNLNGGKWIIKIKKNTAE- 95

XP_013058026.1 Np VETQEEFLYLIRRLQKLQDIKPI-TDLSLFKKGIEPMWEDPSNLNGGKWIIKIKKNTAE- 95

XP_052905898.1 Na IETKEEFLYLIRRMQKLQEIKPI-TDLSLFKSGIEPMWEDPCNINGGKWIIKIKKNTAE- 95

**P P**

Human eIF4E -SDLDRFWLETLLCLIGESFD--------------------DYSDDVCGAVVNVRAKGDK 162

Yeast_eIF4E -ADIDELWLRTLLAVIGETID--------------------EDDSQINGVVLSIRKGGNK 162

NP_594228.1 Spo GNALDEMWLTTVLAAIGETLD--------------------PTGQEVMGVVINMRKGFYR 162

NP_595451.1 Spo -SNLDELWLYMVLAAIGETLD--------------------PTGKEVTGVVCNMRKGFYR 188

XP_013239191.1 Md -KKISKLWKDAIFSCIGSEYE---------------------HPETINGVVLSIRQKTDR 155

XP_008074794.1 Vf NNEIISIWERTILYAASEMVA----------------------EKGICGCVLSPRRSFDR 132

XP_051345132.1 Nh ----HQVWTDTLLFCISELCMLRTVQMEGSELVINESTIDEKLAGVICGAVLSPRKNCVR 158

XP_052904897.1 Na ----EQIWTNTLLFCISESFLVRAPNGVLQDSIISNSTIDRALSGAICGAVFSPRKNYIR 158

XP_047770386.1 Nj ----QDVWTNTLLFCISETVMTRPSSGASTDFIISDATIDRSLAGVICGAVFSPRKNYTR 156

XP_051332673.1 Nm ----SQIWLNTLLFCISESVIVRSSNGITKDFIISDATVDTSLNGVICGAVLSPRKNYTR 158

XP_024332261.1 Vc -YNPEEVFLLTVAFCISNTAA------------------------GICGCVVMSKHEFIK 122

XP_014563204.1 Oc -EDLNGIWKKTMAFCVSNCIE------------------------NICGCVFNEKQSVYK 122

XP_003073379.1 Ei -DSIDELWKRTMAFCVSNTVD------------------------NICGCVLSEKQSFYK 122

NP_597220.1 Ec -DNVGELWKRTMAFCVSNTVD------------------------NICGCVFNEKQSFYK 122

XP_003887769.1 Eh -DNVGDLWKKTMAFCVSNSVD------------------------NVCGCVLSEKQSFYK 122

XP_009265002.1 Er -DNIGELWKKTMAFCISNVVD------------------------NVCGCVLSEKQSFYK 122

XP_013237606.1 Md -SVSTRIWELLLLALIGDEFS--------------------DLQEHICGVVFSIRPYENF 213

XP_013239128.1 Md -SVSTRIWELLLLALIGDEFS--------------------DLQEHICGVVFSIRPYENF 213

XP_008074945.1 Vf ----TRLFQKLLIRMVRQPFD----------------------KIDVNGIVISFRMKNVI 125

XP_051345334.1 Nh ----QRLFESLIMWMGACPFK----------------------TMIVNGVLISVRGHQTI 130

XP_047772122.1 Nj ----QRLFESVFLWMALVPFK----------------------TMDVNGAVISVRGHHTI 128

XP_051334052.1 Nm ----QRLFESLCIWMALVPFK----------------------TMEVNGIVVSVRGHHTI 129

XP_013058026.1 Np ----QRLFESLFIWMALVPFS----------------------TMDVNGIVVSVRGHHTI 129

XP_052905898.1 Na ----QRLFESLFVWMALVPFA----------------------SMEVNGIVVSVRGHHTI 129

**m**

Human eIF4E IAIWTTECENR-EAVTHIGRVYKERLGLPPKIVIGYQSHADTATKS--GST----T-KNRF 215

Yeast_eIF4E FALWTK-SE-DKEPLLRIGGKFKQVLKLTDDGHLEFFPHSSANGRH----PQPSITL--- 213

NP_594228.1 Spo LAVWTK-SCNNREVLMEIGTRFKQVLNLPRSETIEFSAHEDSSKS---GSTRAKTRMSV-- 218

NP_595451.1 Spo IAVWTR-NCNDKDVLEKIGLRFKEVLGISDKETIEYSAHEDSSKA---GSMRAKTRMSL-- 243

XP_013239191.1 Md IAFWISDASHD-DALKHIASFIKTYLELPADFTLSFQLHADALKGN---SRDH----LIF- 207

XP_008074794.1 Vf IALWTKDT--D-EDVVDIGKEWKKVVGYS--KELAFKVHENAMK----GFRERN---NNLY 181

XP_051345132.1 Nh ISIWTSIK--D-NRVLQVGKLWRENGKIPDSFKILFKAHENAIR----GSRDIS-S--DVY 209

XP_052904897.1 Na ISIWTSIK--D-KKVTRIGELWKEFAEIPGNVKISFKVHESAIK----GSKDSS-S--DVY 209

XP_047770386.1 Nj ISIWTSLK--D-RRVTRIGEQWKSFAGIEDSYKLNFKAHESAIK----GSRDPS-T--DVY 207

XP_051332673.1 Nm ISIWTSIK--D-RRVTRIGELWKSFSEISDSIKLNFKAHESAIK----GSRDSS-S--DVY 209

XP_024332261.1 Vc ISLWIENEQY----HDDIMAKWKDVLVRY-DLNIYFLLHKKGI-DGNKGRKTWNKKKAYF- 176

XP_014563204.1 Oc ISIWFGRDYN----QDMIKEKWESVLGNI-NLPIYSFLHKKSL-DGTKGGKKKWGGKREK- 176

XP_003073379.1 Ei VAIWFGKDYN----QDSIKDMWQEALGGG-KLSIYSFLHKKSL-D----SNKGKKKWGGRR 173

NP_597220.1 Ec IAIWFGKDYN----QDVIKDMWQEALGPG-RLSIYSFLHKKSL-D---SSKGKKKW-GGRR 173

XP_003887769.1 Eh LAIWFGKDYN----QDTIKEMWQEALGGG-NLSVYSFLHKKSL-D----SNKGKKKWGGRR 173

XP_009265002.1 Er LAIWFGKDYN----QDTIKEMWQETLESG-SLSVYSFLHKKSL-D----SNKGKKKWGGRR 173

XP_013237606.1 Md IAIWMPKSDDL-PQIQKL-----SLFGLAPDTLIEYKAHNYAKEGVAP-ST--ARS---KA 262

XP_013239128.1 Md IAIWMPKSDDL-PQIQKL-----SLFGLAPDTLIEYKAHNYAKEGVAP-ST--ARS---KA 262

XP_008074945.1 Vf LAVWTKDSTGK-DSFKDVLMEIKKVLDVKFFLSVEYKDNDESLKDN--SSFRN--TKNLYV 181

XP_051345334.1 Nh LSLWTKTCPGSPAEFAAQEAEIRDVLKLKQMIPVIFKGNDESLRDR--SSFRQ--PV---- 183

XP_047772122.1 Nj LSLWTKTCPSD-GEMLEQEAEIREKLEIKPVIPVAFKGNDESLKDK--SSFR--HTV--IV 182

XP_051334052.1 Nm LSFWTKTCPNE-EEMAEQEKEIREKLELKPVIPVSFKGNDESLKDK--SSFR--HTV---- 181

XP_013058026.1 Np LSLWTKSCPSE-EERHIQEKEIRNTLELKQIIPVSFKGNDESLKDK--SSFR--H------ 179

XP_052905898.1 Na LSLWTKTCGTE-EEMAAQEKEIRDKLELKHAIPVTFKGNDESLKDK--SSFRY------IV 181

Human eIF4E VV----------- 217

Yeast_eIF4E ------------- 213

NP_594228.1 Spo ------------- 218

NP_595451.1 Spo ------------- 243

XP_013239191.1_Md ------------- 207

XP_008074794.1_Vf TLA---------- 184

XP_051345132.1_Nh TL----------- 211

XP_052904897.1_Na SL----------- 211

XP_047770386.1_Nj SL----------- 209

XP_051332673.1_Nm SL----------- 211

XP_024332261.1 Vc ------------- 176

XP_014563204.1 Oc ------------- 176

XP_003073379.1 Ei ------------- 173

NP_597220.1 Ec ------------- 173

XP_003887769.1_Eh ------------- 173

XP_009265002.1_Er ------------- 173

XP_013237606.1_Md SIDAPSSVQANKE 275

XP_013239128.1_Md SIDAPSSVQANKE 275

XP_008074945.1 Vf Q------------ 182

XP_051345334.1 Nh --KPTS------- 187

XP_047772122.1 Nj --KEKK------- 184

XP_051334052.1 Nm --KEKPTKQ---- 188

XP_013058026.1 Np --KDKISKQ---- 188

XP_052905898.1 Na --KDKVPK----- 187

**Supplementary Fig. S17.** **Sequence comparison of human eIF4E with Class I and Class II orthologs of the *phylum Rozellomycota****.* eIF4E from the ascomycetes *S. cerevisiae*(yeast) *S. cerevisiae* and *S. pombe* (Spo) were also included to identify the reported phosphorylated residues (14). Full-length proteins were compared. Residues involved in the cap recognition (9-12) are indicated as follows: , residues binding the guanine by  –  interactions; *G*, residue recognizing the guanine ring; *P*, residues interacting with the phosphate groups; *m*, Trp recognizing the cap methyl group. *Asterisks* indicate to W43 and W56 of the human protein used to classify the eIF4E-family members into three classes (13). Residues identical to human eIF4E are shaded in black boxes. Gaps are represented by dashes. Conservative changes are in grey boxes. Purple boxes highlight conservation of phosphorylated amino acids S2, S15, and S28 of *S. cerevisiae* eIF4E (14), and S209 of human/mouse eIF4E (15,16). Phosphorylation of *S. cerevisiae* S28 was demonstrated to increase eIF4G affinity (18). Phospho-mimicking E or D residues in the phosphorylatable positions (18,19) are also highlighted in gray boxes. Class I, F-Subclass IB, and Class II species are in black, blue, and red, respectively. F-Subclass IB- and Class II-specific residues are highlighted in blue and red, respectively. Human eIF4E (acc. numb.M15353) (20); *S. cerevisiae* eIF4E (acc. numb. NP_014502.1) (25,26); *S. pombe* (Spo) eIF4E (27,28). *Rozellomycota* species: Md, *Mitosporidium daphnia*; Vf, *Vavraia culicis floridensis;* Nh, *Nematocida homosporus*; Na, *Nematocida ausbeli*; Nj, *Nematocida major*; Nm, *Nematocida minor*; Vc, *Varimorpha ceranae*; Oc, *Ordospora colligate*; Ei, *Encephalitozoon intestinalis*; Ec, *Encephalitozoon cuniculi*; Eh, *Encephalitozoon hellem*; Er, *Encephalitozoon romaleae*; Np, *Nematocida parisil*.

* ****

Human eIF4E MA//-----TEEEKTESNQEVANPEHYIKHPLQNRWALWFFKND---------KSKTWQANLRLISKF 66

Yeast_eIF4E MS//VSVDDTTA-TPK-TVLSDSAHFDVKHPLNTKWTLWYTKPA-------VDKSESWSDLLRPVTSF 69

NP_594228.1 Spo MQ//VSEPQEKALR---TVFDDKINFNLKHPLARPWTLWFLMPP--------TPGLEWNELQKNIITF 69

NP_595451.1 Spo MA//R-PARLLEGL---SAVNAETAFVKTHPLQHEWTLWFLKPP--------TQGLEWSDLLKEIISF 94

XP_052931884.1 Ma MA-------AAAVTPEVLKLTETIDYTNKHPLQNKWTLWFDNPGK------KTNAQSWADNLKEIITV 55

XP_023463717.1 Rm MA//-----AA------HTEVAQVDYSVKHSLQNTWTLWFDNPGK------KTSAQSWADNLKEIINF 48

XP_051385155.1 Cr MA//------TTLSPEVVQLASTMDYSVKHPLQNTWTFWFDNPGK------KANVQSWADNLKEIVSF 54

XP_051463075.1 Mm MA//-----AAARSAEVQELCQTIDYSVKHPLQNTWTLWFDNPGK------KASAQSWADNLKEIINI 55

XP_051438517.1 Gp MT---------TLPAEVVELCQTIDYSAKHPLQNTWTLWFDNPGK------KANAQSWADNLKEIINI 53

XP_051443732.1 Ur MA-DVNEKVVVVENKNAQDPVLQLDYSIKHPLHNTWTLWFDNPGR------KNSAQNWAQNLKEVFSF 61

XP_051398225.1 Hr MT//EWHNISQDNHKYTVPIPEGLDLTVSHPLQNAWTIWFDNPLT------NTNAQSWAENLKELVTV 68

XP_021882321.1 Lt MA//LTVPTTGSDDKYVTVFNDPVNFNAKHPLHHSWTLWFDNPGK------RSNENTWEQSLKELITF 64

XP_021880830.1 Lt MA//NTLAVPTENTKYVTVFNDPVNFNVKHPLNNSWTLWFDNPGK------KTNPNTWEQSLKELITV 67

XP_051411976.1 Gm MA//NSLQVPTGDNKYVTVFNDPVNFNAKHPLNNSWTLWFDNPGK------KSNANNWEQSLKELITF 68

XP_051416653.1 Gm MA//NSLTVPTEGGKYVTVFNDPVNFNAKHPLNNSWTLWFDNPGK------KSNANNWEQSLKELITF 69

XP_025182496.1 Ri MT//NNSSAFPFDGEMKTVFNDPVNFNVKHPLFNAWTLWFDNPGK------KANTASWSQNLKELITF 84

XP_023466841.1 Rm MS//TNNEKQDDDGAPKTVFDDPKEFNVKHPLQNTWTLWFDNPGK------KANATNWSENLKEIVNV 101

XP_051418803.1 Rs MS//EQPSNAAQET-QGTGQNSNLDLSVKHRLQHEWTLWFDKPGK------KADSESWSQNLKEIVTV 66

XP_052976982.1 Zm MA//AAESNDKKEEPVTTVFHDPINYNVKHPLHNAWTLWFDNPGK------KANTQSWSQNLKEIVTV 102

XP_058341318.1 Lo MA//TNATESGQEQPVTTVFHDRINYNVKHPLHNSWTLWFDNPHK------KANAASWSQNLKEIVTV 90

XP_051448585.1 Ur MS//STETKGSEDDQPVTIFHDRMNYNVKHPLHNEWTLWFDNPGK------KANVQSWSQNLKEIVTF 88

XP_018290543.1 Pb MA//GKTNDQENDS-MKTVFHDREHYNVKHPLQNTWTLWFDNPGK------KANVTSWSQNLNEVVSV 88

XP_018287057.1 Pb MT//KSADAVADNS-ITTVFHDRSNYNVKHPLQNTWTLWFDNPGK------KANTQSWSQNLKEIVSI 81

XP_052941272.1 Ma MT//PVQEDTTKEDRPITVFEDPKNYNVKHPLQNTWTLWFDNPGK------KASATNWSQNLKEIVDV 68

XP_051386388.1 Cr MT//PSEQ-QNDES-LKTVFHDPKNYNVKHPLQNTWTLWFDNPGK------KANAASWSQNLKEIVDV 72

XP_051458734.1 Mm MT//VEQGNNDDNS-VRTVFHDAKNYNVKHPLQNTWTLWFDNPGK------KANAASWSQNLKEIVNV 74

XP_051431805.1 Gp MT//EQQQQQISDEPIKTVFHDSKNYNVKHPLQNTWTLWFDNPGK------KANAASWSQNLKEIVNV 69

XP_051401788.1 Hr -------------------INYNVKHPLQNTWTLWFDNPGK------KANTQSWSQNLKEIVSM 39

XP_051423503.1 Rs MC//KS-ANATGDEPITTVFHDRLNYNVKHPLHNTWTLWFDNPGK------KANAQSWSQNLKEIVSV 113

** G P**

Human eIF4E DTVEDF---WALYNH--IQLSSNLMP--GCDYSLFKDGIEPMWEDEKNKRGG-RWLITLNK 119

Yeast_eIF4E QTVEEF---WAIIQN--IPEPHELPL--KSDYHVFRNDVRPEWEDEANAKGG-KWSFQLR- 133

NP_594228.1 Spo NSVEEF---WGIHNN--INPASSLPI--KSDYSFFREGVRPEWEDVHNKTGG-KWAFQNK- 133

NP_595451.1 Spo KTVEEF---WGIFKT--ISKASMLPA--KSDYSYFLKGIRPEWEDPQNMNGG-KWAYQSK- 138

XP_052931884.1 Ma DTVEDF---WSTFNN--VAKVNHLTP--NANYHFFKEGVRPEWEDPANAEGG-KFTIQFPK 108

XP_023463717.1 Rm DTVEDF---WSTFNN--VSKINHLSP--NSNFHLFKQGIRPEWEDPSNAEGG-KFGIQLPK 101

XP_051385155.1 Cr DTVEDF---WSTMNN--VAKVNHLAL--NSNYHLFKQGVRPEWEDEANAEGG-KFSIQFPK 107

XP_051463075.1 Mm DTVEDF---WSAFNN--IAKVNHLGP--SSNYHLFKQGIRPEWEDESNAEGG-KFGIQFPK 108

XP_051438517.1 Gp DTVEDF---WSAFNN--IAKVNHLAP--NSNYHLFKQGVRPEWEDEANAEGG-KFGIQFPK 106

XP_051443732.1 Ur STVEDF---WSSWSN--ITKVTRLDV--GSNCFVFKKGIRPEWEDPINENGG-KFSVQFQR 114

XP_051398225.1 Hr DTVEEF---WGAFNN--LTKVDQLEP--NSNYHFFKKGIRPEWEDPANANGG-KFSIQFPR 121

XP_021882321.1 Lt DTVEDF---WGVYNN--IMKACDLSI--NSNYHLFKHGIKPMWEDPANKHGG-KWSIQLPR 117

XP_021880830.1 Lt ETVEDF---WGVYNN--VMKACDLAI--NSNYHLFKQGIKPMWEDPANKRGG-KWSIQLPR 120

XP_051411976.1 Gm DTVEDF---WGVYNN--IMKTCDLSV--SSNYHLFKQGIKPMWEDSANKHGG-KWSIQLPR 121

XP_051416653.1 Gm DTVEDF---WGVYNN--IMKTCDLGI--SSNYHLFKQGIKPMWEDPANKRGG-KWSIQLPR 122

XP_025182496.1 Ri DSVEEF---WGVYNN--VAKAIDLSP--GSNYHLFKQGIKPMWEDPVNELGG-KWVIQFPR 137

XP_023466841.1 Rm NTVEDF---WGVYNN--IPKVSILEV--NSNYHVFKKGVRPEWEDPFNANGG-KFSVQLPR 154

XP_051418803.1 Rs DTVEDF---WGVYNN--ITKVNKLEAGSSCNYHFFKKGIRPEWEDPANAKGG-KFGIQFPK 121

XP_052976982.1 Zm ETVEDF---WGVYNN--IAKVNHLES--NSNYHFFKKGVRPEWEDPANANGG-KFSIQFPR 155

XP_058341318.1 Lo ETVEDF---WGVYNN--IAKVNHLES--NSNYHFFKKGIRPEWEDPANAEGG-KFSIQFPR 143

XP_051448585.1 Ur KTIEDF---WAVFNN--IVKVSRLDI--SSNYHLFKKGVRPEWEDPANEHGG-KFSVQLPK 141

XP_018290543.1 Pb STVEDF---WGVYNN--VAKVNHLEI--SSNYHFFKKGVRPEWEDPANSKGG-MFSIQLPR 141

XP_018287057.1 Pb DTVEDF---WGVYNN--ISKVNHLEI--SSNFHFFKKGVRPEWEDPMNAEGG-KFGIQFPR 134

XP_052941272.1 Ma NTVEDF---WGVHNN--IVKVNHLEI--SSNYHIFKKGVRPEWEDPVNASGG-KFSIQFPR 121

XP_051386388.1 Cr NTVEDF---WGVHNN--IVKVNHLEI--SSNYHVFKKGIRPEWEDPANANGG-KFSIQFPR 125

XP_051458734.1 Mm DTVEDF---WGVHNN--IVKVNHLEI--SSNYHVFKKGIRPEWEDAANANGG-KFSIQFPR 127

XP_051431805.1 Gp DTVEDF---WGVHNN--IVKVNHLEI--SSNYHVFKKGIRPEWEDPANANGG-KFSIQFPR 122

XP_051401788.1 Hr DAVEDF---WGVYNN--IVKVDRLDL--SSNYHLFKKGVRPEWEDPANAKGG-KFSIQFPR 92

XP_051423503.1 Rs DTVEDF---WGVYNN--IVKVNHLDV--SSNYHIFKKGIRPEWEDAANANGG-KFSIQLPR 166

**P P m**

Human eIF4E QQ-RRSDLDRFWLETLLCLIGESFDDY---SDDVCGAVVNVRAKGDKIAIWTTECENRE- 174

Yeast_eIF4E GKG--ADIDELWLRTLLAVIGETIDED---DSQINGVVLSIRKGGNKFALWTKSE-DKE- 173

NP_594228.1 Spo GRGG-NALDEMWLTTVLAAIGETLDPT---GQEVMGVVINMRKGFYRLAVWTKSCNNRE- 175

NP_595451.1 Spo -HKG-SNLDELWLYMVLAAIGETLDPT---GKEVTGVVCNMRKGFYRIAVWTRNCNDKD- 101

XP_052931884.1 Ma TK-AGEAINEYWMSILMSVVGEQLA-T---EEEICGVVVSIRKSFYRLALWIKTSKDEE- 162

XP_023463717.1 Rm SK-AGDAINEHWMNLLLAVIGEQLA-T---EDEICGAVVSVRKSFYRIALWVKTSKDEE- 155

XP_051385155.1 Cr NK-AGDAINEYWTYLLLAVIGEQLA-T---EEEICGAVISVRKTFYRIALWIKTSNDSE- 161

XP_051463075.1 Mm NK-AGEAINDYWMYLLLAVIGEQLA-S---DEEICGAVISVRKSFYRIALWVKTSDDEE- 162

XP_051438517.1 Gp NK-AGEAINEYWMYLLLAVIGEQFP-S---D-EVCGAVISVRKSFYRIALWVKSSDDEE- 159

XP_051443732.1 Ur TKQMGEAVNQLWLNAILACIGEQFK-L---EHEICGVVLSVRKSFFRLALWTKSANNRD- 169

XP_051398225.1 Hr AR-SGNTINLFWLNMVLAVIGEQFV-----DDEVCGTVIAIRRNYFRIALWTKTAQRNE- 174

XP_021882321.1 Lt NK-TITEIDNIWLYTMLACIGEAFE-H---ENEVCGVVVSVRKGFFRIALWTRSSDNRD- 171

XP_021880830.1 Lt NK-TISEIDNIWLYTMLACIGEAFE-H---ENEVCGAVVSVRKAFFRIALWTRSSDNQE- 174

XP_051411976.1 Gm NK-TMSDIDNIWLYTMLACIGEAFE-H---ESEVCGVVVSVRKAFFRIALWTRSSDNRD- 175

XP_051416653.1 Gm NK-TMNEIDNIWLYTMLACIGEAFE-Q---ESEVCGAVVSVRKAFFRIALWTRSSDNYE- 176

XP_025182496.1 Ri NK-TGEDINTLWLYTMLACIGEGFD-Y---ADEVCGAVVSVRKIFYRISLWTRTSNNRE- 191

XP_023466841.1 Rm NR-TGEAINDYWLNLMLAMFGEQFQ-Y---EDEICGAVVSVRKVFYRIALWTKTSQRNE- 208

XP_051418803.1 Rs NK-TGEAINTYWLYLLLALIGEQFD-N---ADEICGAVVSVRKMFFRIALWIKDSEQTK- 175

XP_052976982.1 Zm NR-TGEGINDYWLSLLLAMIGEQFA-H---ENEVCGAVISVRKVFFRVALWIRSSERNE- 209

XP_058341318.1 Lo NR-TGETINKYWLDMLLAMIGEQFA-H---EHEICGAVISVRKVFFRLALWIRSSDRNE- 197

XP_051448585.1 Ur NR-TGEAINDLWLYTLLACIGEQLP-N---EDEVTGAVVSVRKVFFRISLWTKTSDNRE- 195

XP_018290543.1 Pb NR-TGEGVNEYWLQMLLAVIGEQYK-Y---EDEICGAVVSVRKVFYRIALWIKTSDNGD- 195

XP_018287057.1 Pb NR-TGEAINDYWLHLLLAMIGEQFA-K---EDEICGAIVSVRKFFFRVSLWVKHSEKNE- 188

XP_052941272.1 Ma NR-TGEAINDYWLNLILAMLGEQFQ-Y---EDEICGAVVSVRKVFYRVALWIKSSERNE- 175

XP_051386388.1 Cr NR-TGEAINDYWLNLILTMLGEQFK-Y---EDEICGAVVSVRRVFYRVALWIKSSEKNE- 179

XP_051458734.1 Mm NR-TGESINDYWLNLILAMLGEQFQ-Y---EDEICGAVVSVRKVFYRVALWIKSSEKNE- 181

XP_051431805.1 Gp NR-TGEAINDYWLNLILAMLGEQFE-Y---EDEICGAVVSVRKVFYRVALWIKSSEKNE- 176

XP_051401788.1 Hr NR-TGEAINNYWLHTILAMIGEQFA-Y---EDEICGAVVSVRKVFFRIALWIKSSENEQ- 146

XP_051423503.1 Rs NR-TGEAINDYWLYTILAMIGEQFA-Y---EDQICGAVVSVRKVFYRIAVWIKSSDDNE- 220

Human eIF4E --A-VTHIGRVYKERLG-LPPKIV---IGYQSHADTATKSG-STTKNRFVV--------- 217

Yeast_eIF4E --P-LLRIGGKFKQ-V--LK-LTDDGHLEFFPHS-SANGRHPQPSITL------------ 213

NP_594228.1 Spo --V-LMEIGTRFKQ-V--LN-LPRSETIEFSAHEDS-SKSG-STRAKTRMSV-------- 218

NP_595451.1 Spo --V-LEKIGLRFKE-V--LG-ISDKETIEYSAHEDS-SKAG-SMRAKTRMSL-------- 243

XP_052931884.1 Ma --K-IEALRSEVRQALS-LHEEIP---IEFSVHADATAKVTAS-LAATSLDENEKAAS--…229

XP_023463717.1 Rm --K-IASISKQLRDALN-LPEAIT---IDFVPHGDATAKLAAE-LAATQLEEQAENKS--…230

XP_051385155.1 Cr --K-IEKISEQLRETLN-LSEDIP---IEFHIHNDASAKAAAA-LAAASLEDKKEDNA--…233

XP_051463075.1 Mm --K-IEKISQQLREVLN-LAEDIP---IEFTAHGESPAKVAAA-LAAASLEENAATEA--…229

XP_051438517.1 Gp --K-VNKISQQIRDLLS-LSDEIP---VEFTPHRESPAKVAAA-LAAASLEDNTDAPK--…227

XP_051443732.1 Ur --L-AEALGDQGGFGIR-GS---------YCRV------H------ATW----------- 193

XP_051398225.1 Hr --A-LESIGHNIRELLN-LPMHLT---MDFIPHESAQIH---E----KFTL--------- 211

XP_021882321.1 Lt --V-IMGIGRTLKSQTS-ID--GQ---FDFQSHHDSKG-GA------KWTV--------- 206

XP_021880830.1 Lt --I-VMNIGRTLKRSAN-IH--GT---LEFQSHHDSTPKA--E----PWTV--------- 210

XP_051411976.1 Gm --V-AMSIGRTLKGQIG-VD--GA---FEFQPHHDTKTGGK------PWVV--------- 211

XP_051416653.1 Gm --T-AMSIGRILKRAAN-LN--GT---LEFQSHHESSNTGK------AWTV--------- 212

XP_025182496.1 Ri --I-CETLGRQLKQTLG-LTPVQQ---LEFQPHSDSIKSGNHN-KEHFFV---------- 233

XP_023466841.1 Rm --K-IETIG--------------------------------------------------- 214

XP_051418803.1 Rs --E-IEHIKQQLEQILA-IPDNVQ---IDFVKHGEPAPKRP-SVSQ-------------- 213

XP_052976982.1 Zm --I-TEKLGAQIKEFLT-VPPNMT---IEFTPHGESAAKS--S---QKFTV--------- 248

XP_058341318.1 Lo --T-TETLGRQIKEFLD-IPSNLN---LEFTPHGESAAKSA-----QKFTL--------- 236

XP_051448585.1 Ur --V-LDGIGKKLKETLN-IP-NMP---LEFTPHSDAATKGP-SETTGRFTI--------- 237

XP_018290543.1 Pb --I-VKTIGTQIKEFLS-VPSNIK---VEFTPHGDSATKS--S--PNRRII--------- 235

XP_018287057.1 Pb --T-LEALGRQVKEVMN-VPDNIP---VEFTPHGETPSENA----V-KFVVQ-------- 228

XP_052941272.1 Ma --K-IETIGRQLKEFLN-LNSNLT---VEFTPHSDSSNKSG-E---NRFTV--------- 215

XP_051386388.1 Cr --T-TETIGRQLKEFLN-LNSSLV---VEFTPHGDSAAKSG-E---NKFTI--------- 219

XP_051458734.1 Mm --K-IETIGRQLKEFLN-LNNTLV---VEFTPHGDSAAKS--S--ENRFTI--------- 221

XP_051431805.1 Gp --K-IETIGRQLKEFLQ-LQNTLV---VEFTPHGDSAVKSN-E---NRFTI--------- 216

XP_051401788.1 Hr --I-VQTIGQQLKEFLE-VPPNLQ---VEFAPHGDAPA---------------------- 177

XP_051423503.1 Rs --I-IEKLGRQLKEFLS-VPNNIP---VEFTPHGDHGPAKG----ANKITI--------- 260

**Supplementary Fig. S18.** **Sequence comparison of human eIF4E with Class I orthologs of the *phylum* *Mucoromycota****.* eIF4E from the ascomycetes *S. cerevisiae*(yeast) *S. cerevisiae* and *S. pombe* (Spo) were also included to identify the reported phosphorylated residues (14). Full-length proteins were compared. Residues involved in the cap recognition are indicated as follows: , residues binding the guanine by  –  interactions; *G*, residue recognizing the guanine ring; *P*, residues interacting with the phosphate groups; *m*, W recognizing the cap methyl group. *Asterisks* indicate W43 and W56 of the human protein used to classify the eIF4E-family members into three classes (13). Residues identical to human eIF4E are shaded in black boxes. Conservative changes are in grey boxes. Purple boxes highlight conservation of phosphorylated amino acids S2, S15, and S28 of *S. cerevisiae* eIF4E (14), and S209 of human/mouse eIF4E (15,16). Phosphorylation of *S. cerevisiae* S28 was demonstrated to increase eIF4G affinity (18). Phospho-mimicking E or D residues in the phosphorylatable positions (18,19) are also highlighted in gray boxes. Gaps are represented by dashes. // indicates that some residues are not shown. Human eIF4E (acc. numb.M15353) (20); *S. cerevisiae* eIF4E (acc. numb. NP_014502.1) (25,26); *S. pombe* (Spo) (27,28) eIF4E. Mucoromycota species: Ma, *Mycotypha africana*; Rm, *Rhizopus microspores*; Cr, *Cokeromyces recurvatus*; Mm, *Mucor mucedo*; Gp, *Gilbertella persicaria*; Ur, *Umbelopsis ramanianna*; Hr, *Halteromyces radiatus*; Lt, *Lobosporangium transversale*; Gm, *Gamisella multidivaricata*; Ri, *Rhizophagus irregularis*; Rs, *Radiomyces spectabilis*; Zm, *Zychaea mexicana*; Lo, *Lichtheimia ornata*; Pb, *Phycomyces blakesleeanus*.

Human 4EHP ---MN--------------NKFDALKDDD-SG---DHDQNEENSTQKDGEKEK----T-- 33

Human eIF4E ------------------------MATVE--------------------------PETTP 10

Yeast eIF4E -------------------------MSVE------EVSKKFEENVSVD--------DTTA 21

XP_025189698.1 Ri -------------------------MSIS------GVGEDKDNKEKENGNNS-------- 21

XP_051408324.1 Rm ///--HGSKS---GVLSVAGNSSGLAAISGSGSGSGLGPGGGGSLSGSASG--------- 125

XP_021883259.1 Lt ///SSKLAHGISGNQSRPPAVTGGVSSS-SVESSGSGSGTGTSAAGSTVAGTSKST---- 147

XP_021882609.1 Lt ///ISQYAGGIESE-SAPTTHGALSNLGSFAGGNGELGKGSSGEELHASSKSTTTSLSTS 167

XP_051411806.1 Gm ---------------------------------------------------------MAA 3

XP_051440155.1 Ur MDTAQSAAWSSSKIPNQLIDNYNSASSKRPNANISLSPMTPLFA--SSTTTHHLSSKLGK 58

XP_052982541.1 Zm ---MN----------ASTESSL----SIQ------SI----------------------- 14

XP_058342203.1 Lo ---------------------M----DIQ------S------------------------ 5

XP_018298042.1 Pb ----------------------------------------MW----AN------------ 4

XP_052931499.1 Ma MQHTTSMLDTMSTMTNNNTTLNSSQHTM-DNQ---PFTMNLE----QH------------ 40

XP_051386935.1 Cr ----------------------------------------MH----QS------------ 4

XP_051438632.1 Gp ------------------------------------------------------------ 0

XP_023465093.1 Rm -------------------------MS------------FN-----GS------------ 6

XP_051455757.1 Mm ------------------------------------------------------------ 0

XP_058345801.1 Lo --------------------------------------MNFT----TT------------ 6

XP_051400110.1 Hr -------------------------MS--------------------------------- 0

XP_051427038.1 Rs -------------------------MT-------------------SL------------ 4

*

* ****

Human 4EHP ER------DKNQSSSKRKAVVPGPAEHPLQYNYTFWYSRRTPGRPTSSQSYEQNIKQIGT 87

Human eIF4E TPNPPTTEEEKTESNQEVANPEHYIKHPLQNRWALWFFKNDK-----SKTWQANLRLISK 65

Yeast eIF4E TP--------KTVLS---DSAHFDVKHPLNTKWTLWYTKPAVDK---SESWSDLLRPVTS 67

XP_025189698.1 Ri --------NGLVLTRQQFTPSPTSEIHPLHFTWVFWFMHRNPGSKI--LNYESSMKKIAA 71

XP_051408324.1 Rm -----------SVGSTALGSTGIVSRHPLHFNWVFWFMHRAPGSKI--LNYEGAMKKIAT 172

XP_021883259.1 Lt ----------AVPLT----GISTGSLHPLHFNWVFWFMHRAPGSKI--VNYESSMKKIAT 191

XP_021882609.1 Lt TP------T-LSSGNVLLHIPGSSETHPLHYNWVFWFMHRAPGSKI--LNYESSMKKITT 218

XP_051411806.1 Gm TP------AAIGGGG--GGAGVSPGIHPLQFNWVFWFMHRAPGSKI--LNYESSMKRIAG 53

XP_051440155.1 Ur SP------AETLTVKLLDQQIPEDQKHPLHYTWVFWFMHRPPGAKI--TNYESGMKKIAS 110

XP_052982541.1 Zm ------NTALAAVPS---AAQTQQDEHPLHYTWVFWFMHRLPKAKI--KDYEGSMKRIAA 63

XP_058342203.1 Lo --------------T--APAASVGTHHPLSSTWVFWFMHRLPKAKI--KDYEGSMKRIAS 47

XP_018298042.1 Pb -------NNHLELTS-QGQKASGTQTHPLHYTWVFWFMHRNPREKI--TNYEESMKRIAS 54

XP_052931499.1 Ma --------HYQQQQQQQQQTGAVQQYHPLKYGWVFWFMHRSRGEKI--TDYEAAMKRIAS 90

XP_051386935.1 Cr E--------NLDSIPQPNLRLVETKSHALHYGWVFWFMHRSPGEKI--VNYEGAMKKIAT 54

XP_051438632.1 Gp -----------------MNQPLEQHSHSLQYGWVFWFMHRSRGEKI--TNYEGAMKKIAT 41

XP_023465093.1 Rm --------VEL---S--NTPSTRPESHALRYGWVFWFMHRSPGEKI--VDYEGAMKKIAT 51

XP_058345801.1 Lo T------QTMLHDTSV---PKQQQEQHALRYTWVFWFMHRTRGAKI--TNYEEGMKRIAA 55

XP_051400110.1 Hr ----------PGKTSS--------LQHPLHYSWVFWFMHRSPGSKI--TNYEGNMKQIAS 42

XP_051427038.1 Rs T--------DSQDIS-VTEKSADSERHPLRYTWVFWFMHRPPRAKI--TNYEGAMKRIAA 53

**G**  **P**

Human 4EHP FASVEQFWRFYSHMVRPGDLTGHSDFHLFKEGIKPMWEDDANKNGGKWIIRLRKG----L 143

Human eIF4E FDTVEDFWALYNHIQLSSNLMPGCDYSLFKDGIEPMWEDEKNKRGGRWLITLNKQQRRSD 125

Yeast eIF4E FQTVEEFWAIIQNIPEPHELPLKSDYHVFRNDVRPEWEDEANAKGGKWSFQLRGKG--AD 125

XP_025189698.1 Ri FSSIEDFWAVYSHLRRPHELPNISDYHLFKQGVRPVWEDDTNINGGKWIVRLKKG----L 127

XP_051408324.1 Rm FGSAEDFWAVYSHLKRPHELPTVSDYHLFKQGVRPVWEDETNIHGGKWIVRLKKG----L 228

XP_021883259.1 Lt FGSVEDFWAVYSHLKRPHELPTVSDYHLFKQGVRPVWEDATNINGGKWIVRLKKG----L 247

XP_021882609.1 Lt FGSVEAFWAVYSHLRRPNELPHVSDYHLFKQGVRPVWEDPANISGGKWIVRLKKG----L 274

XP_051411806.1 Gm FGSVEAFWGIYSHLRRPHELPHVSDYHLFKKGVRPVWEDPVNINGGKWIVRLKKG----L 109

XP_051440155.1 Ur VSSIEDYWAVYSHLKRPKDLPNISDYHLFKQGVRPVWEDTMNVNGGKWIVRLKKG----L 166

XP_052982541.1 Zm FSSIEEFWAVYSHLRRPSDLPNISDYHLFKMGVRPVWEDEANIHGGKWIVRLKKG----L 119

XP_058342203.1 Lo FSTVEEFWAVYSHLRRPSDLPTISDYHLFKQGVRPVWEDDANIRGGKWIVRLKKG----L 103

XP_018298042.1 Pb FSSIEDFWAVYSHLRRPSDLPNISDYHLFKHGVRPVWEDEVNVNGGKWIVRLKKG----L 110

XP_052931499.1 Ma FKTVEEFWAVYSRLSRPSELPNISDYSLFKDGVRPVWEDNVNINGGKWIVRIKKG----L 146

XP_051386935.1 Cr FNTIEEFWAVYSRLRRPNELPNISDYHLFKEGVRPVWEDEANINGGKWIVRVKKG----L 110

XP_051438632.1 Gp FHTIEEFWAVYSHLRRPGELPNISDYHLFKEGVRPVWEDTANIHGGKWIVRVKKG----L 97

XP_023465093.1 Rm FKTVEEFWAAYSHLRRPSDLPNISDYHLFKQGVRPVWEDDVNINGGKWIVRVKKG----L 107

XP_058345801.1 Lo FSTVEDFWAVYSHLRRARDLPIVSDYQLFKQGVRPVWEDNANINGGKWIVRLKKG----L 111

XP_051400110.1 Hr FSSVEEFWAVYSHIKRPHELPTISDYHLFKHGVRPMWEDEANIQGGKWIVRLKKG----L 98

XP_051427038.1 Rs FSSIEDFWAVYSHLTRPHDLPNISDYHLFKHGVRPVWEDNANINGGKWIVRLKKG----L 109

**P P m**

Human 4EHP ASRCWENLILAMLGEQFMVGE-EICGAVVSVRFQEDIISIWNKTASDQATTARIRDTLRR 202

Human eIF4E LDRFWLETLLCLIGESFDDYSDDVCGAVVNVRAKGDKIAIWTTECENREAVTHIGRVYKE 185

Yeast eIF4E IDELWLRTLLAVIGETIDEDDSQINGVVLSIRKGGNKFALWTKSEDK-EPLLRIGGKFKQ 184

XP_025189698.1 Ri ASRYWESLVMAVIGDQFDVGT-EICGAVLSIRSSEDILSLWNQSAHEGRINLKIRDTMKR 186

XP_051408324.1 Gm ASRYWEDLVIAVIGDQFEVGT-EICGAVLSIRGSEDILSLWNQSAHEGRINLKIRDTMKR 287

XP_021883259.1 Lt ASRYWENLVMAVIGDQFDVGS-EICGAVLSIRGGEDILSLWNQSAHEGRINLKIRDTMKR 306

XP_021882609.1 Lt ASRYWENLAMAVIGDQFDVGS-EICGIVLSIRGAEDILSIWNKSADEGRINLKIRDTMKR 333

XP_051411806.1 Gm ASRYWENLVMAVIGDQFDVGE-EICGIVLSIRGAEDILSIWNQSADEGRINLKIRDTMKR 168

XP_051440155.1 Ur ASRYWESLVMAIIGDQFDVGQ-EICGAVLSIRNSEDILSVWNQSAHEGRTNLKIRDTMKK 225

XP_052982541.1 Zm ASRYWEQLVLAIIGEQFDVQG-EICGAVLSIRNSEDIISVWNRTASKGRINLKIRDTIKK 178

XP_058342203.1 Lo ASRYWEQLLLAIVGEQFDVGD-EICGAVLSIRSSEDIISVWNKTAANGRINLKIRDTIKK 162

XP_018298042.1 Pb ASRYWESLVLAIIGDQFDVND-EICGAVLSIRGSNDIVSVWNKTSSNGRINLKIRDTIKK 169

XP_052931499.1 Ma ASRYWESLVLAIIGDQFDVED-EICGLVLSIRGSEDIISVWNKTSSNGKINLKIRDTIKK 205

XP_051386935.1 Cr ANRYWESLVLAIIGDQFDVQD-EICGAVLSIRGSEDIISVWNKTSFDGKINLKIRDTIKK 169

XP_051438632.1 Gp AGRYWESLILAIIGDQFDVQD-EICGAVLSIRGSEDIISVWNKTSSNGKINLKIRDTIKK 156

XP_023465093.1 Rm ASRYWESLVLAIIGDQFDVND-EICGAVLSIRGSEDIISVWNKTSSNGKINLKIRDTIKR 166

XP_058345801.1 Lo ATRYWELLVLAIIGEQFDVND-EICGAVLSIRSSEDIISVWNKTSSNGRINLKIRDTIKK 170

XP_051400110.1 Hr ASRYWENLVLAIVGDQFDVQD-EICGAVLSIRNSEDIISVWNKTSSNGRINLKIRDTIKK 157

XP_051427038.1 Rs ASRYWESLVLAIIGEQFDVND-EICGLVLSIRSSEDIISVWNKTSSNGRINLKIRDTIKK 168

Human 4EHP VLNLPPNTIMEYKTHTDSIKMPGRLGPQRLLFQNLWKPRLNVP 245

Human eIF4E RLGLPPKIVIGYQSHADTATKSGSTTKNRFVV----------- 217

Yeast eIF4E VLKLTDDGHLEFFPHSSANGRHPQPSITL-------------- 213

XP_025189698.1 Ri VLNLPSETIMEYKTHNDALKDNSSFRNTDVFR----------- 218

XP_051408324.1 Gm VLNLPADTIMEYKTHNDALKDNSSFRNTDVFR----------- 319

XP_021883259.1 Lt VLNLPADTIMEYKTHNDALKDNSSFRNTDVFR----------- 338

XP_021882609.1 Lt VLDLPIDTVMEYKSHNDALKDNTSFRNTDIFR----------- 365

XP_051411806.1 Gm VLNLPADTIMEYKSHNDALKDNTSFRNTDVFR----------- 200

XP_051440155.1 Ur VLNLPADTIMEYKTHNDSLNDHSSFRNTDIFR----------- 257

XP_052982541.1 Zm VLSLPQDTTMEYKTHNDSLKDNSSFCNTDVFR----------- 210

XP_058342203.1 Lo MLNLPAETTMEYKTHNDSLRDKSSFCNTDVFR----------- 194

XP_018298042.1 Pb TLGLPQDTIMEYKSHNDALRDNSSFHNTDVFR----------- 201

XP_052931499.1 Ma YLNLPPDTTMEYKTHNDALRDNSSFRNTDVFR----------- 237

XP_051386935.1 Cr HLNLPAETTMEYKTHNDALRDNSSFRNTDVFR----------- 201

XP_051438632.1 Gp YLNLPVETTMEYKTHNDALRDNSSFRNTDVFR----------- 188

XP_023465093.1 Rm HLNLPADTIMEYKTHNDALRDKSSFRNTDVFR----------- 198

XP_058345801.1 Lo VLSLPADTVMEYKSHNDALKDRSSFRNTDIFR----------- 202

XP_051400110.1 Hr VLALPQDTTMEYKSHNVALRDNSSFRNTDVFR----------- 189

XP_051427038.1 Rs VLNLPQDTIMEYKTHNDALKDNSSFRNTDVFR----------- 200

**Supplementary Fig. S19.** **Sequence comparison of human 4EHP with selected Class II orthologs of the *phylum* *Mucoromycota****.* Full-length proteins were compared. Human and yeast *S. cerevisiae* eIF4Es were also included to identify the reported phosphorylated residues (14-16).Full-length proteins were compared with Clustal Omega.Residues involved in the cap recognition are indicated as follows: , residues binding the guanine by  –  interactions; *G*, residue recognizing the guanine ring; *P*, residues interacting with the phosphate groups; *m*, W recognizing the cap methyl group. *Asterisks* indicate residues equivalent to W43 and W56 of the human protein used to classify the eIF4E-family members into three classes (13). Residues identical to human 4EHP are shaded in black boxes. Conservative changes are in grey boxes. Changes of W56 to Y and the change K/R162 to I, V, or L that define Class II proteins are highlighted in red. Gaps are represented by dashes. /// iindicates that some residues are not shown. Purple boxes highlight conservation of phosphorylated amino acids S2, S15, and S28 of *S. cerevisiae* eIF4E (14), and S209 of human/mouse eIF4E (15,16). Phosphorylation of *S. cerevisiae* S28 was demonstrated to increase eIF4G affinity (18). Phospho-mimicking E or D residues in the phosphorylatable positions (18,19) are also highlighted in gray boxes. Human 4EHP (acc. numb. AF047695) (29); Human eIF4E (acc. numb.M15353) (20); *S. cerevisiae* eIF4E (acc. numb. NP_014502.1) (25,26). *Mucoromycota* species: Ri, *Rhizophagus irregularis*; Gm, *Gamsiella multidivaricata;* Lt, *Lobosporangium transversale*; Ur, *Umbelopsis ramanniana*; Zm, *Zychaea mexicana*; Lo, *Lichtheimia ornata*; Pb, Phycomyces blakesleeanus; Ma, *Mycotypha africana*; Cr, *Cokeromyces recurvatus*; Rm, *Rhizopus microsporus*; Gp*, Gilbertella persicaria*; Hr, *Halteromyces radiatus*; Rs, *Radiomyces spectabilis*.

Human eIF4E MA//-----TEEEKTESNQEVANPEHYIKHPLQNRWALWFFKND---------KSKTWQANLRLISKF 66

Yeast_eIF4E MS//VSVDDTTA-TPK-TVLSDSAHFDVKHPLNTKWTLWYTKPA-------VDKSESWSDLLRPVTSF 69

NP_594228.1 Spo MQ//VSEPQEKALR---TVFDDKINFNLKHPLARPWTLWFLMPP--------TPGLEWNELQKNIITF 69

NP_595451.1 Spo MA//R-PARLLEGL---SAVNAETAFVKTHPLQHEWTLWFLKPP--------TQGLEWSDLLKEIISF 94

XP_051421482.1 Rs MM//VRENPTRYSYVKQYMKENALPNTSSLELPFSVKFCFSDTSST-----KQKPVHYSSTIQHVFDC 516

XP_051438379.1 Gp ML//IAENPTRFQYLMAYLRQQQ--FPPSLPLQKSCLFYFSQNT-----------SDYVNAIQCMLQV 359

XP_023469098.1 Rm MQ//VKENPTRYEHAVSQLNEMHIPFDSALPLAVPCTFYFTDNSSK-----Q--ADSYLESVKPLFDC 272

XP_051451119.1 Mm MF//VQENPTRFEEATSYLRENQISFSSSLPLLKPCTFYFSDTNIK-----Q---HCYISAVNAVFQC 472

XP_051443983.1 Ur MY//MKEHPTEFQKAVAYLKENGIPFDSSLPLLHNVILYFSDTSTAK----AKSANSYSSTIRPMFMC 474

XP_021884632.1 Lt MS//AKGDAQLEELQRIRLFLAQRQYPDEMPLSDEWTLFFSDTSGAKDKT--GVQDAYSSAITPLFSC 845

XP_021877286.1 Lt MS//-------------------------------------------QQGMPDKAPPFIVE---L--- 290

XP_051413118.1 Gm MS//AKGDAQLEELERIRQFLAARQYPDEMPLSDEWTLFFSDTSRAKDKS--GVQDAYSSAITPLFSC 866

XP_051416333.1 Gm MP//SLNPPDQNSLNQQDHVSPMHTSHTSHPLQHAWTLYY-DTSAGYNRQSSSLH-NYENGLRDLGTF 431

XP_025167616.1 Ri MN//LTDSKDTIRIRRQLEIISKDNYPDSLPLSNKWTMYFADTSVTKTNTRIISKNKYSSTLNPLFEC 346

XP_025184179.1 Ri ------------------------------------------------VQFARY---------- 6

XP_051439445.1 Gp MS//---------YVITPETRQKLLAMGQLPLESEWTFWYDKFVP------NLSATDYEANLKIIATI 119

XP_051455009.1 Mm MV//---YKD-QLIILDEETRLKLTHMGQLPLDAEWTFWYDKFVP------NLPASDYESNLKVISTA 140

XP_052985340.1 Zm MT//---YKDE-VVILDKEQKEELLKVGEIGLQSEWTFWYDRYVP------NLPPTEYEANLQVISTV 215

XP_051401403.1 Hr MG//---YKD-KVVILDQERKEQLLKVGTILLQEEWTYWYDRYVP------NLPASEYEANLQIISTV 180

XP_058348974.1 Lo MP//---YKDEIVIL-DEKKKEELLKVGEIRLQSEWTFWYDRYVP------NLSASEYEANLQVISTT 189

XP_018290203.1 Pb ---------------------------------------------------------------- 0

XP_051426684.1 Rs ---------------------------------------------------------------- 0

XP_025185094.1 Ri MS//---NNSLRSSKESTTDKDNTKSSEQYPLTNEWTFYHDKYVA------NATPEEYEENLKSVATV 89

XP_021883158.1 Lt MP//---DNGEAKS-PAVDSNIVAAAATPIPLRHEWVFWHDKFVA------NATPAEYTENLREIADV 79

XP_051416061.1 Gm MP//---DNGESKSPSGSENGVSAAVAVPIPLKNEWVFWHDKFVA------NATPAEYTENLREIADV 78

** G P**

Human eIF4E DTVEDF---WALYNH--IQLSSNLMP--GCDYSLFKDGIEPMWEDEKNKRGG-RWLITLNK 119

Yeast_eIF4E QTVEEF---WAIIQN--IPEPHELPL--KSDYHVFRNDVRPEWEDEANAKGG-KWSFQLR- 133

NP_594228.1 Spo NSVEEF---WGIHNN--INPASSLPI--KSDYSFFREGVRPEWEDVHNKTGG-KWAFQNK- 133

NP_595451.1 Spo KTVEEF---WGIFKT--ISKASMLPA--KSDYSYFLKGIRPEWEDPQNMNGG-KWAYQSK- 138

XP_051421482.1 Rs TTVWQFSARWRLFKQL-RAKPSQLLP--NQNVYCFESDVEPMWEDPVNQHGG-RLTLC--P 571

XP_051438379.1 Gp DTVWKFSSCWRTLKG--YKKPSEFSI—-NQNLYCFVQGVQPMWEDPVNAKGGGRLVI---- 412

XP_023469098.1 Rm DTVWNFVCRWRLYKETCQKKPSQLAP--NQNIFCFVQGVKPMWEDPINKKGG-RLNVQ--V 328

XP_051451119.1 Mm DTVWQFSSRWRLYKQTYSKKPSQLLP--NQNLFCFINGVEPMWEDKVNEKGG-RLTITIQN 530

XP_051443983.1 Ur ETVWQFSSRWRKFKDR-FSAPSQMMP--NQNLYFFRQGVEPMWEDPINAKGG-RMTLSP-- 529

XP_021884632.1 Lt HTVPQFATSWKYVRE--RVRPATMKM--NQNLHWFKKGIKPMWEDPKNKYGG-RLTLCPPK 901

XP_021877286.1 Lt -----F--NW-------IEKPHKMEN--SANYHLFKDGIKPMWEDPANANGG-RWIVTLLN 338

XP_051413118.1 Gm HTVPQFATSWKYVRE--RVRPATMKV--NQNLHWFKKGIKPMWEDPKNKYGG-RLTLCPPR 922

XP_051416333.1 Gm TVELFARY-F--NW--IEKPHKMEN---NSNYHLFKDGIKPMWEDPANANGG-RWIVTLLD 484

XP_025167616.1 Ri TTVPELCSNLRKFSS--KIKPSDMKT--NANLSFFKGNIMPMWEDEANQKGG-RFTICP-- 400

XP_025184179.1 Ri SFCRYF--NW-------VKKPSQLDM--NTNFHIFKDKIKPMWEDPANANGG-KWVISM-- 49

XP_051439445.1 Gp SSVQKF---WSIYNN--IDGPERLGF--RSNYHFMKKGIKPIWEDPHNEYGG-SYHFKIPK 172

XP_051455009.1 Mm KTVQKF---WSIYNN--IDGPDRLGF--RSNYHFMKTGIKPIWEDPQNEYGG-SYNFKINK 193

XP_052985340.1 Zm GTVQKF---WSIYNN--IDGPDQLGF--RSNLHFMRKGIKPIWEDPQNEHGG-SYNFKIPK 268

XP_051401403.1 Hr GTVQKF---WSVYNN--IDGPEKLGF--RSNLHFMRKGIKPIWEDPKNEYGG-SFNFKVNK 233

XP_058348974.1 Lo GSVQKF---WSVYNN--IDGPDRLGF--RSNLHFMRKGIKPIWEDPQNENGG-SYNFKIPK 242

XP_018290203.1 Pb ----KF---WSVYNN--IDGPEKLGF--RSNLHFMRKGIKPIWEDPRNEYGG-SFNFKIPK 49

XP_051426684.1 Rs ---QKF---WSVYNN--IDGPEKLGF--RSNLHFMRKGIKPIWEDPHNEYGG-SYNFKIPK 50

XP_025185094.1 Ri NTVQSF---WSVYNN--IIGPDQLQF--RSSLHFMKSGIRPVWEDPHNENGG-AWSFRVNK 142

XP_021883158.1 Lt NTVQTF---WSVYNN--ITGPERLTM--RCSLHFIHKGIKPLWEDPKNEHGG-AWNFRTAK 132

XP_051416061.1 Gm NTVQSF---WSVYNN--ITGPERLSL--RCSLHFIHKGVKPLWEDPKNEHGG-AWNFRTAK 131

**P P m**

Human eIF4E QQ-RRSDLDRFWLETLLCLIGESFDDY---SDDVCGAVVNVRAKGDKIAIWTTECENRE- 174

Yeast_eIF4E GKG--ADIDELWLRTLLAVIGETIDED---DSQINGVVLSIRKGGNKFALWTKSE-DKE- 173

NP_594228.1 Spo GRGG-NALDEMWLTTVLAAIGETLDPT---GQEVMGVVINMRKGFYRLAVWTKSCNNRE- 175

NP_595451.1 Spo -HKG-SNLDELWLYMVLAAIGETLDPT---GKEVTGVVCNMRKGFYRIAVWTRNCNDKD- 101

XP_051421482.1 Rs P---KASLDSLFEWILCTFVGGNLM-----DHGVRGLVLSRRNRGDRIELWTDGQGAQH- 622

XP_051438379.1 Gp V---SHQLDNLFEWLLCAFVGGQLY-----DEGCVGVVVSKKAHGDRVELWFDQYMTQE- 463

XP_023469098.1 Rm N---LKLLDEVFESILFALVGAGLI-----MLGTVGVAVSKRYRGDRIELWLDESVTED- 379

XP_051451119.1 Mm N-GKHLDDVSEWIICALVGGGIY-------DYGVVGIVVSKRNRGDRIELWLDESNTTN- 581

XP_051443983.1 Ur -T--KMALDEIWEVVLAAFVGGTC------HNSVVGVVMSRRGRGDRIEIWMDEQGREA- 579

XP_021884632.1 Lt A-----LLDIVWETVLILMAGDVLD-L---NGEGTGAVIARRPRGDRVEVWVGAEDTPE- 951

XP_021877286.1 Lt KN--AELLDRCWMEMAYALVGEQLD-A---GDDICGAVLSRRIKADRLAVWVRDKENVE- 391

XP_051413118.1 Gm A-----LLDIVWETVLILMAGDVLD-H---HGEGTGAVFARRTRGDRVEVWLGADDTPE- 972

XP_051416333.1 Gm KN--SELLDRCWMELAYALVGEQLD-A---GDDICGAVLSRRTRADRLAVWVRDKENVE- 537

XP_025167616.1 Ri -P--RNQLNSLWDSIVLLLAGETID-D---KDLICGAVCARRDRGDRVELWISGDAYSR- 452

XP_025184179.1 Ri KS--PQLLDRCWSWLVYALVGEELD-E---NDDICGAVMSRRARGDRIAVWVRDKDNVP- 102

XP_051439445.1 Gp Q-----YTKTTWRDILVLLIGGQLEDSI--KNTIYGVSISSRQHVDNYQIWTAQNNTTL- 224

XP_051455009.1 Mc Q-----QSSLAWRDILVLLIGEKVEDWI--KNTVFGVSVSSRQHVDNYQIWTAHHNKNI- 245

XP_052985340.1 Zm A-----HSPLAWRDLLVLLIGEKIEGWV--GDTVCGVSVSTRQQFDNYQVWTAHTHHSEN 321

XP_051401403.1 Hr N-----QSPIVWRDLLVLLIGEKVEHWL--NDTVCGVSVSSRQHCDNYQIWVSNGQDKKT 286

XP_058348974.1 Lo H-----HSPLAWRDLLVLLIGEKVEGWL--GDVVCGVSVSSRQQCDNYQIWTAHNHQNDP 295

XP_018290203.1 Pb A-----QSPLAWRDLLVLLIGERVEGCI--DDTVCGVSVSSRQQCDSYQIWTANGHNSA- 101

XP_051426684.1 Rs S-----HSPIAWRDLLVLLIGEKVEGWL--EDTVCGVSVSSRQQCDNYQIWTAGPSDEA- 102

XP_025185094.1 Ri S-----ESKIVWRELLMLLIGEQFEDVVSKDDDIFGLSVSTRFNADIFTIWNKNAGAHE- 196

XP_021883158.1 Lt A-----DTAFVWRELLMALIGEQFEDTIAKGDQIFGLSVSARWNSDIFQIWNMDSSLKD- 186

XP_051416061.1 Gm G-----DTAYVWRELLMALIGEQFEDTIAKDDQIFGLSVSARWSSDIFQIWNMDSSLKE- 185

Human eIF4E --A-VTHIGRVYKERLG-LPPKIV---IGYQSHADTATKSG-STTKNRFVV--------- 217

Yeast_eIF4E --P-LLRIGGKFKQ-V--LK-LTDDGHLEFFPHS-SANGRHPQPSITL------------ 213

NP_594228.1 Spo --V-LMEIGTRFKQ-V--LN-LPRSETIEFSAHEDS-SKSG-STRAKTRMSV-------- 218

NP_595451.1 Spo --V-LEKIGLRFKE-V--LG-ISDKETIEYSAHEDS-SKAG-SMRAKTRMSL-------- 243

XP_051421482.1 Rs --A-MTTLKEKLYQLIP-QHQHAIIETARFKKHFDK------------------------ 654

XP_051438379.1 Gp --K-VPLLKEKLCSLLS-SSCHHEINSSRYKKHFL------------------------- 494

XP_023469098.1 Rm --K-IPELKNTLKTLLP-VSCHGEIDSSRYKKHFM------------------------- 410

XP_051451119.1 Mm --QSISQFKYVIKHNLL-ITSSLLILNFILENTFVLYYLLLATM----KLMQVDIKSIFN//693

XP_051443983.1 Ur --S------DEVK----------------------------------------------- 584

XP_021884632.1 Lt --A-LAHIRGVLIQELAPSGADEVVRVAKYKKHFDSRKEEKMKQ---LRET-AAATTI--//1142

XP_021877286.1 Lt --A-INGIGKRLIQILD-LAKERI--TLEFQITTDTRSSGPPKNYITLDAIRKALAQE--//525

XP_051413118.1 Gm --A-LAHIRGVLCQELAASGADELVKTAKYKKHFDGRREEKLKQ---QQLA-KEAAAAAG//1166

XP_051416333.1 Gm --V-INGIGYILRHDQL-YNSCAH----HFL-------------WLSL------------ 564

XP_025167616.1 Ri --D-IDRIRDLLSMELG-HEMK------EMKNVKYKKHLGKP------------------ 484

XP_025184179.1 Ri --V-INGIG--------------------------------------------------- 108

XP_051439445.1 Gp --S-DEVVKSALNELLQ--PTEIQ--SIYFKIHKTHADFRPSTPTFSKTLK--------- 268

XP_051455009.1 Mm --Q-DSIVRAKLEELLY--PADIQ--SFYFKMHKTHADFQKPTTPTSAHSP--K------…329

XP_052985340.1 Zm VA--DGKVRSKLTELLR--PAEIQ--SFYYKVHKNHAAFQKPSQHHNQHQH-QHQHHH--…451

XP_051401403.1 Hr DQD-NAAVKKALADLLD--PAEIQ--SFYFKIHKNHAAFQKDSSTNSLNRSTGNN----… 399

XP_058348974.1 Lo EA--DARVKAKLIEVLR--PAEIQ--SFYYKVHKNHAAFQKQQSPPNHANN-KDSN----…409

XP_018290203.1 Pb -Q--DVEVQNQLASLMK--PAEIQ--SFYFKSKLFFRFLLCKGVEKRY------------ 142

XP_051426684.1 Rs -R--EATIRSKVVDLLK--PAEIQ--SFYFKSK--------------------------- 128

XP_025185094.1 Ri ----NSKIMDKLQELLLQENIKLQ--SPYYKVHKEHAAFKKQDTNGKD------------ 238

XP_021883158.1 Lt ----NATVMDKVAEILK--DIQIQ--SPFYKAHKDHDHFKM------------------- 219

XP_051416061.1 Gm ----NATVMDKVGEILK--GVQIQ--SPFYKAHKDHDHFKM------------------- 218

**Supplementary Fig. S20.** **Sequence comparison of human eIF4E with F-Class** **VI and F-Class VII orthologs of the *phylum* *Mucoromycota****.* eIF4E from the ascomycetes *S. cerevisiae*(yeast) *S. cerevisiae* and *S. pombe* (Spo) were also included to identify the reported phosphorylated residues (14). F-Class VI and F-Class VII proteins are indicated in blue and green, respectively. Residues involved in the cap recognition are indicated as follows: , residues binding the guanine by  –  interactions; *G*, residue recognizing the guanine ring; *P*, residues interacting with the phosphate groups; *m*, W recognizing the cap methyl group. *Asterisks* indicate W43 and W56 of the human protein used to classify the eIF4E-family members into three classes (13). Residues identical to human eIF4E are shaded in black boxes. Conservative changes are in grey boxes. Purple boxes highlight conservation of phosphorylated amino acids S2, S15, and S28 of *S. cerevisiae* eIF4E (14), and S209 of human/mouse eIF4E (15,16). Phosphorylation of *S. cerevisiae* S28 was demonstrated to increase eIF4G affinity (18). Phospho-mimicking E or D residues in the phosphorylatable positions (18,19) are also highlighted in gray boxes. Gaps are represented by dashes. // indicates that some residues are not shown. Residues that define F-Class VI and F-Class VII are highlighted in blue and green, respectively. Human eIF4E (acc. numb.M15353) (20); *S. cerevisiae* eIF4E (acc. numb. NP_014502.1) (25,26); *S. pombe* (Spo) eIF4E (27,28). Mucoromycota species: Rs, *Radiomyces spectabilis*; Gp, *Gilbertella persicaria*; Rm, *Rhizopus microspores*; Mm, *Mucor mucedo*; Ur, *Umbelopsis ramanianna*; Lt, *Lobosporangium transversale*; Gm, *Gamisella multidivaricata*; Ri, *Rhizophagus irregularis*; Zm, *Zychaea mexicana*; Hr, *Halteromyces radiatus*; Lo, *Lichtheimia ornata*; Pb, *Phycomyces blakesleeanus*.

**
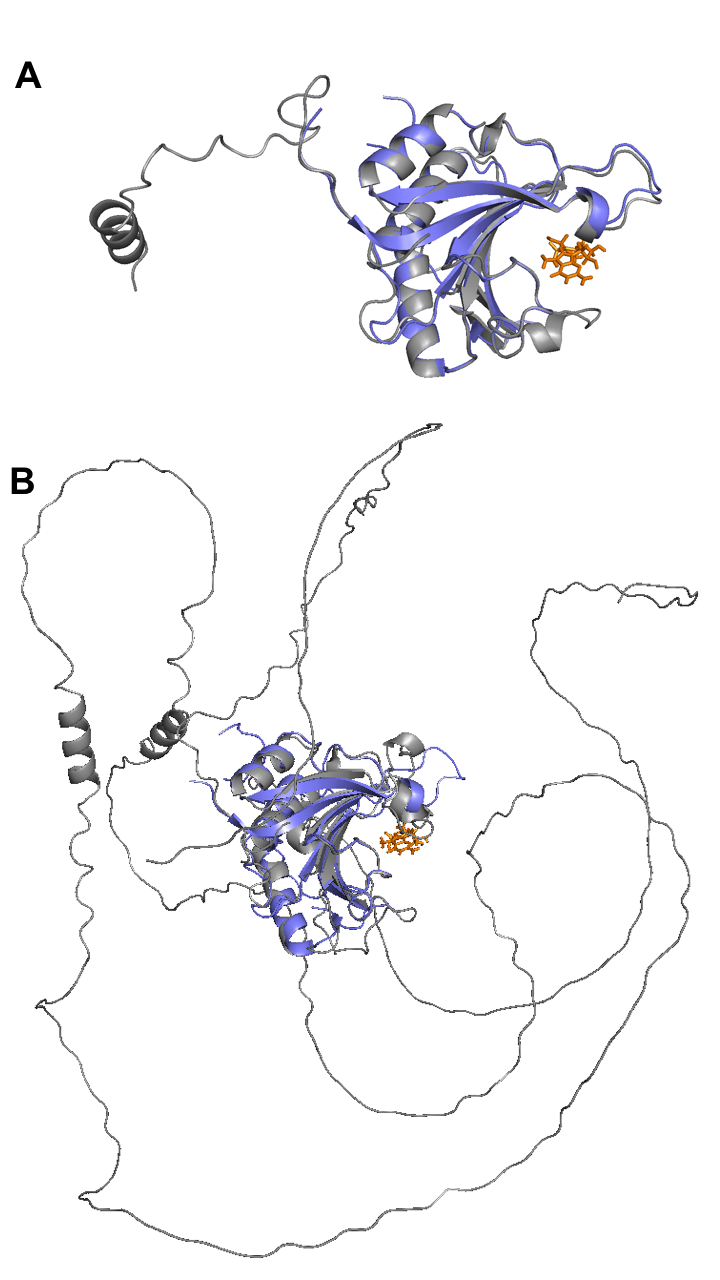
**

**Supplementary Fig. S21.** **Three-dimensional eIF4E structure prediction of *S. cerevisiae* and *Cryptococcus gattii* eIF4E.**The structures were predicted with Alphafold2 and were aligned with the PDB *S. cerevisiae* eIF4E structure 6FC1 (8) (blue) and the m^7^GTP cap (orange).**A)** *S. cerevisiae*,Class I (PDB structure 6FC1). **B)** *Cryptococcus gattii* (gray), F-Subclass IA. The cap structure size is 16–20 Å (1.6–2.0 nm).


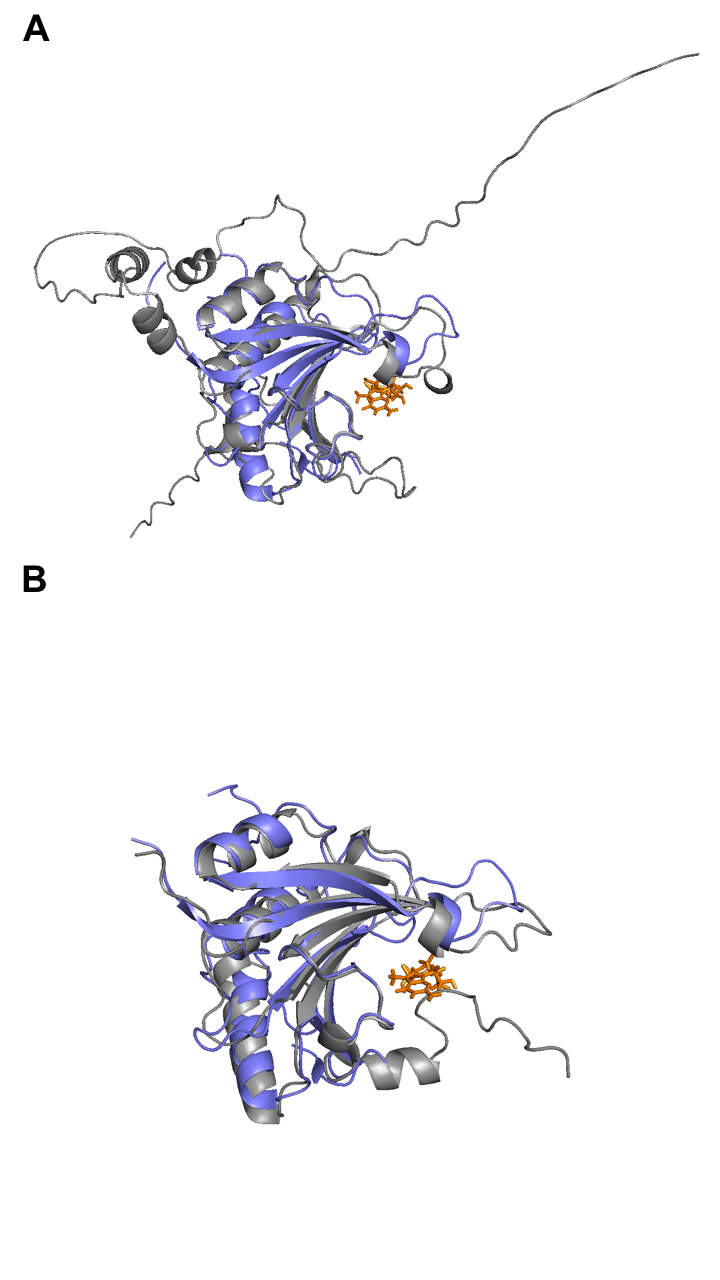


**Supplementary Fig. S22.** **Three-dimensional eIF4E structure prediction of *Mitosporidium daphnia* and *Nematocida major****.* The structures were predicted with Alphafold2 and were aligned with the PDB *S. cerevisiae* eIF4E structure 6FC1 (8) (blue) and the m^7^GTP cap (orange).**A)** *Mitosporidium daphnia* (gray), F-Sublcass IB. **B)** *Nematocida major* (gray), Class II. The cap structure size is 16–20 Å (1.6–2.0 nm).

**
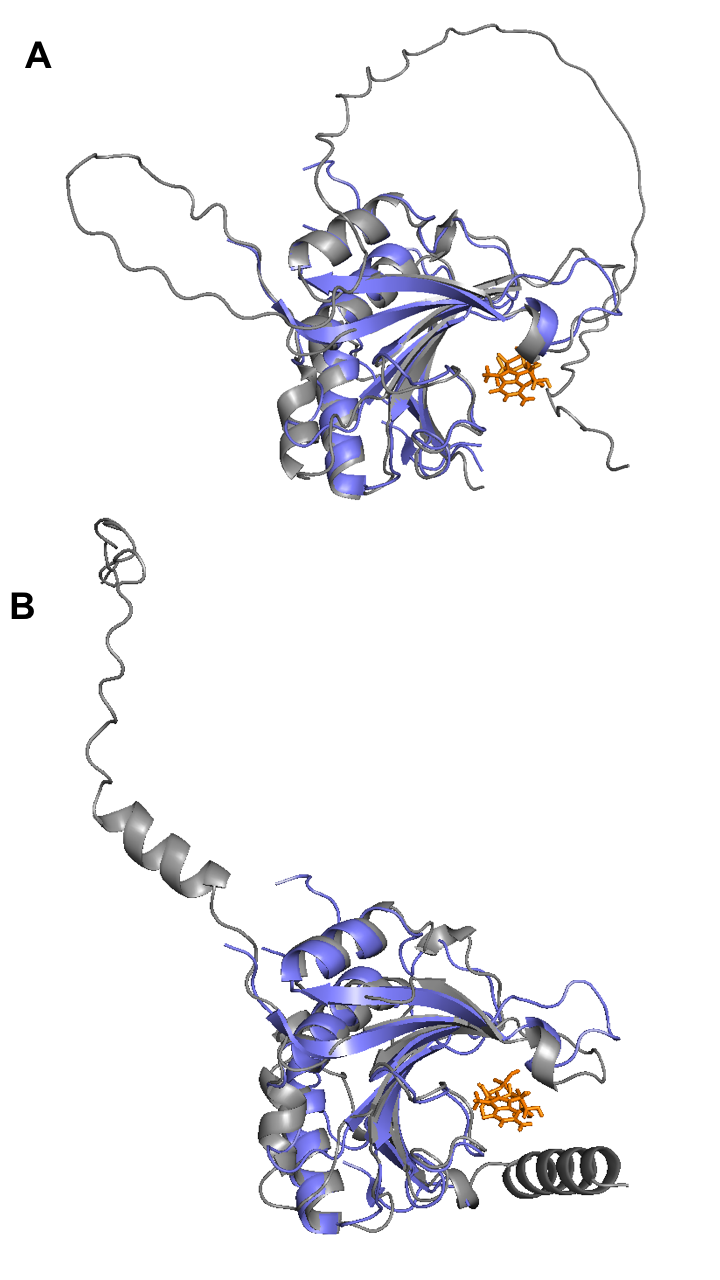
**

**Supplementary Fig. S23.** **Three-dimensional eIF4E structure prediction of *Punctularia strigosozonata* and *Uncinocarpus reesi*.**The structures were predicted with Alphafold2 and were aligned with the PDB *S. cerevisiae* eIF4E structure 6FC1 (8) (blue) and the m^7^GTP cap (orange).**A)** *Punctularia strigosozonata* (gray), Class II. **B)** *Unicarpus reesi* (gray), F-Class IV. The cap structure size is 16–20 Å (1.6–2.0 nm).


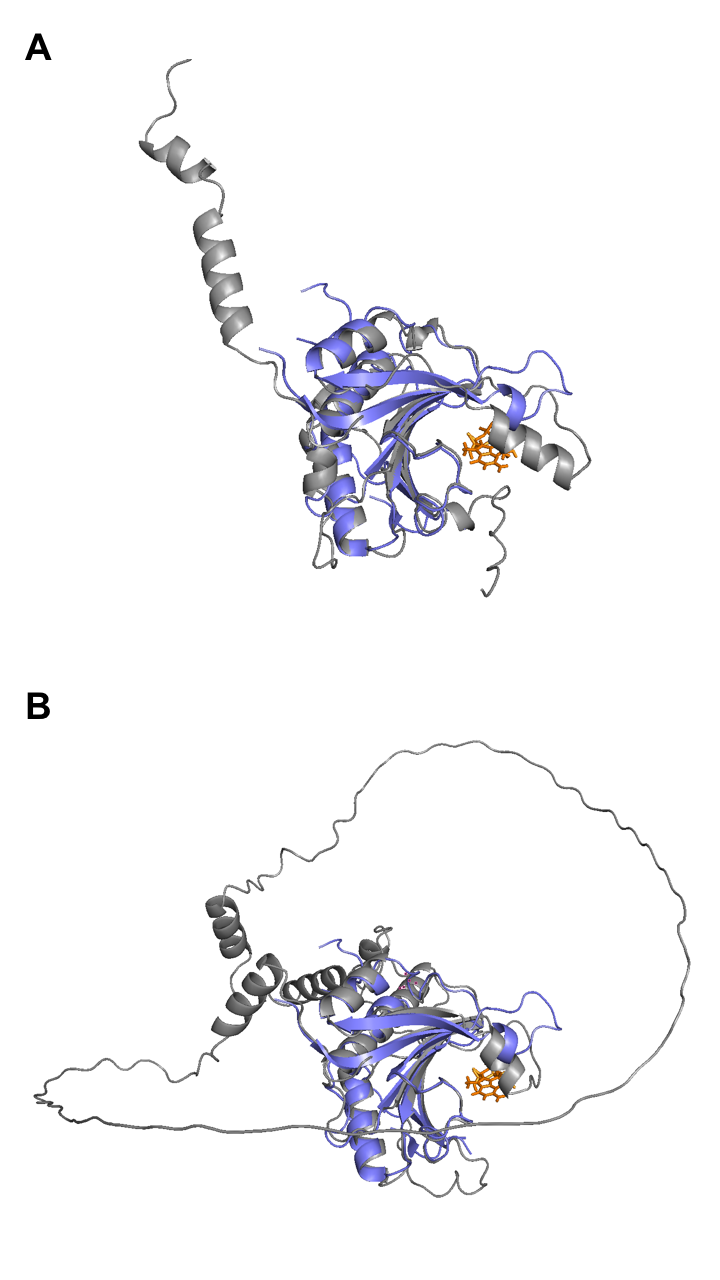


**Supplementary Fig. S24.** **Three-dimensional eIF4E structure prediction of *Ascochyta rabiei* and *Malassezia globose.*** The structures were predicted with Alphafold2 and were aligned with the PDB *S. cerevisiae* eIF4E structure 6FC1 (8) (blue) and the m^7^GTP cap (orange). **A)** *Ascochyta rabiei* (gray), F-Class V. **B)** *Malassezia globose* (gray), F-Class VI. The cap structure size is 16–20 Å (1.6–2.0 nm).


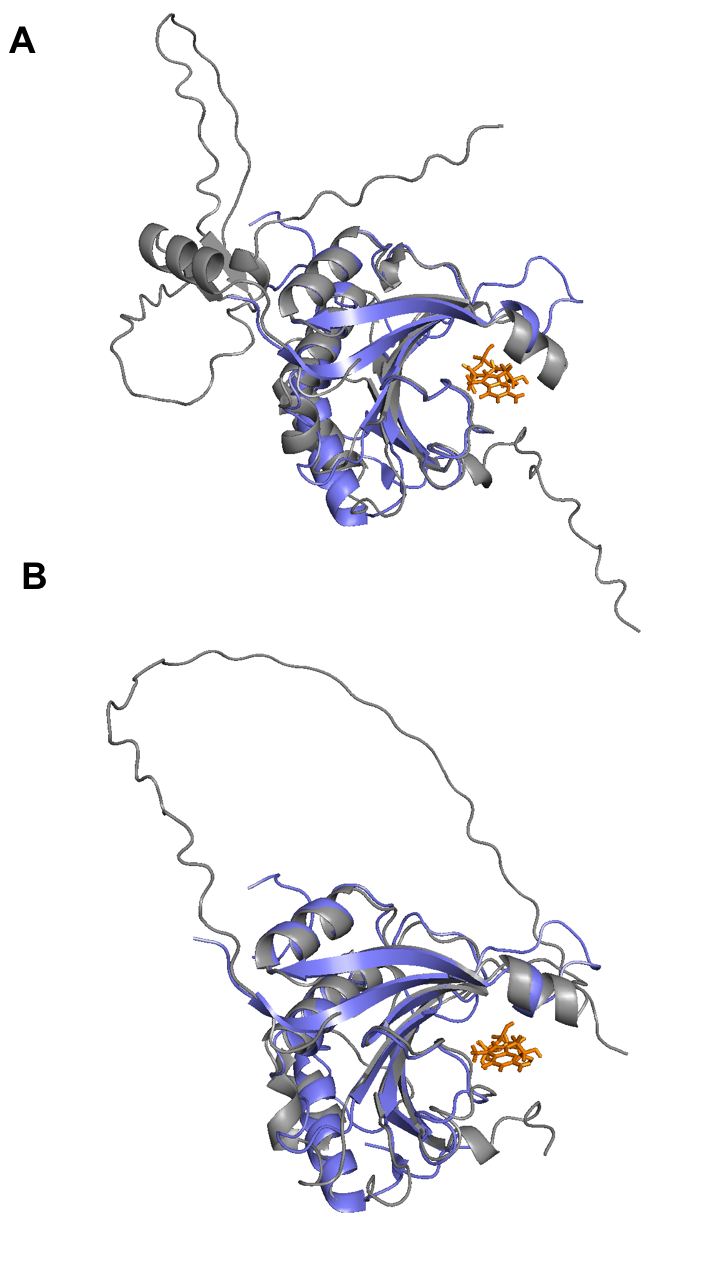


**Supplementary Fig. S25.** **Three-dimensional eIF4E structure prediction of *Gilbertella persicaria* and *Gamisella multidivaricata*.** The structures were predicted with Alphafold2 and were aligned with the PDB *S. cerevisiae* eIF4E structure 6FC1 (8) (blue) and the m^7^GTP cap (orange). **A)** *Gilbertella persicaria* (gray), F-Class VII. **B)** *Gamisella multidivaricata* (gray), F-Class VII. The cap structure size is 16–20 Å (1.6–2.0 nm).

*

eIF4E Human -MATV-EPETTPTPNPPT--TEEEK--TESNQEVANPEHYIKHPLQNRWALWFFK----- 49

eIF4E1 Tc -MSDVKSKEAGANPAASKSGAGEDVAETKLAVPRSLDTGSKKHPLNSSWTLWYDSLST-- 57

eIF4E1 Tb MMAESSAKEMEANQV---SAAGDQTA-KADDRYVIIDRGVKRHLLNRPWTLWYDSVST-- 54

eIF4E1 Ba ---------------------------MT---GSQTSVGKSSHPLHRSWTLWYDSPST-- 28

eIF4E1 Em ---------------------------MSAPSSAHPKKMTNLHKLQRSWTLWYDSPST-- 31

eIF4E1 Lm ---------------------------MSAPSSVPPHKMANLHKLQRAWTLWYDSPST-- 31

eIF4E2 Em ------------------------------------------------------------ 0

eIF4E2 Lm ---------MDPNTCAPA---------------SAVTDEQPLTLLWGTWEMWCDMPQRQQ 36

eIF4E2 Ba -----------------------------------MSEDASLTLLWGTWEMWCDLPALPQ 25

eIF4E2 Tc ----M-QTRLKSRPDEPK---------------LAVHPTSCITPLWGVWEMWCVLPDPVG 40

eIF4E2 Tb ----M-QTLLRPRPGEPT---------------LFGPPTNQCTQLWGVWEMWCVLPDGHE 40

*

****

eIF4E Human ----------------------NDKSKTWQANLRLISKFDTVEDFWALYNHIQLSSNLMP 87

eIF4E1 Tc -----------------------YDSERWELSLVEVITVRTVEEFFTMLHYCKPPHVLRV 94

eIF4E1 Tb -----------------------YDCKQWELSLIEVMTVRTVEDFFAMLHYCKPPHVLRV 91

eIF4E1 Ba -----------------------YNADSWELSLVPIMTVRTVEEFFVMLKYMKPLHALRT 65

eIF4E1 Em -----------------------YNTDNWEMSLVPIMTVHSVEEFFLMLRYMKPLHALRT 68

eIF4E1 Lm -----------------------YNTENWEMSLVPIMTVHSVEEFFVMLRYMKPLHALRT 68

eIF4E2 Em ------------------------------------------------------------ 0

eIF4E2 Lm GQS--------------------TENTNWLEQVKSIGLFDSAEGFWGIFNCTILPSQLPP 76

eIF4E2 Ba RKPAGHHDGKKTRIGK-GAVMSMANDKNWLDRVRSIGLFDSAEGFWGLAQCTLLPSQLPA 84

eIF4E2 Tc GAVGNNTGVRGSAVKKGCAVHKEGNRATWLDQVRSIGLFDNAEGFWGIATCTLPPSQLPP 100

eIF4E2 Tb AAT-ATPNVRGSGAKKGGMSKTKVKKATWLDQVRSIGLFDSAEGFWGIITCTLNPSQLPP 99

**G P**

eIF4E Human GCDYSLFKDGIEPMWEDEKNKRGGRWLITLNKQQR------------------------- 122

eIF4E1 Tc SAQYHFFREGVKPMWEDPSNKAGGKLWISLDDKPVGDVGGRKWENANTSGAAGGEKDNAS 154

eIF4E1 Tb SAQYHFFREGVKPMWEDPNNKAGGKLWVSLDDKTMTDKSG----------AAGGGKTR-- 139

eIF4E1 Ba SSQYHFFQEGVKPMWEDKANKEGGKLWSNLEIAKGNPNKNS------------------- 106

eIF4E1 Em SSQYHFFQEGVKPMWEDPANKKGGKLWVNLDIKSANGRSNNS------------------ 110

eIF4E1 Lm SSQYHFFQEGVKPMWEDPANKKGGKLWVNLDITSANGRSSNN------------------ 110

eIF4E2 Em -------------MWEHEANRRGGKWVIPFTGKVS------------------------- 22

eIF4E2 Lm NGSYYLFRKHIAPMWEHEANRRGGKWVIPFTGKAS------------------------- 111

eIF4E2 Ba GGSYYLFRKHIAPMWEHEANRRGGKWIISLHTSLDAHARKEE------------------ 126

eIF4E2 Tc GITYYMLRRNIAPMWEHEANRRGGRWVVRFQDQQHRQQRKGE------------------ 142

eIF4E2 Tb GFNYYLFRRNIAPMWEHEANRRGGRWVMRFRVXQHDDSPATA------------------ 141

eIF4E Human -----------------------RSDLDRFWLETLLCLIGESFDD-------YSDDVCGA 152

eIF4E1 Tc AATNNSNSNQNAKAKKEEQETEKKPELDTMWENLLMAMVGEYLDP-----EPDGDHIMGL 209

eIF4E1 Tb --------------KDNGTDADKKPELDTVWENVLIALVGEYLDY-----GVEGEHIMGV 180

eIF4E1 Ba -----------------DTASNEKVELDVMWENILMALVGEALEH----DTDGEPHIMGV 145

eIF4E1 Em -NNNNSNSN-SGATVADGGPMESKTDLDKAWENVLMATVGEYLECLDKKNTSAESFVTGI 168

eIF4E1 Lm -N------T-SGTSAADGSAAEAKTDLDKAWENVLMATVGEYLDCVDKKDTPTEPFVTGI 162

eIF4E2 Em ------------------RDGGDLQAVDVAWQTLCLSAIGELFPA-------DEVEICGV 57

eIF4E2 Lm ------------------RSEGDLQPVDEAWQTLCLSAIGELFPG-------DEEEICGV 146

eIF4E2 Ba ------------PR--KEGDGTVVMPVDVAWEKLCLAAIGEMFPA-------DEEEICGV 165

eIF4E2 Tc ------------NEEEI--TEDRSSQVDEAWESLCIALIGEQLPC-------PETEVCGV 181

eIF4E2 Tb ------------LDGAVAAAAEAQLPVDRAWEALCVAMIGEQLPG-------DETEICGA 182

**P P** **m**

eIF4E Human VVNVRA--------------------------KGDKIAIWTTECENREAVTHIGRVYKERL 187

eIF4E1 Tc VLSRRK--------------------------YHNRIALWLRDASASEAVARIEKKLVKEA 244

eIF4E1 Tb VLTKRK--------------------------YCNRIALWLKDASDSDAVAAIEKQLVKEA 215

eIF4E1 Ba VMAKRK--------------------------YVNRLAVWMRDANASEAVEGIKKKLVQEV 180

eIF4E1 Em VMSKRK--------------------------YHNRLAVWVSDASATEKIDALKKTLTKEA 203

eIF4E1 Lm VMSKRK--------------------------YHNRLAVWVSDASATDKIEALKKALTKEA 197

eIF4E2 Em TVS-RGRQRASPS------------GHTTSALSEWKLCLWTRSADDKDSQMRIAEYISAQL 105

eIF4E2 Lm TVS-RGRQRTLPS------------GHATSALSEWKLCLWTRSADNRGSQIRIAEYIRKQL 194

eIF4E2 Ba TVS-RAKGRHSSISSRHSYPGQPPQRTQSTSGGEWKLCLWTRTANDTETQMNIGRYLQALL 225

eIF4E2 Tc ALR-RAERR-----------------------REWKLSLWTRTAADRETQLCIGRFMKTLL 218

eIF4E2 Tb VVR-RAERR-----------------------RDWKLSLWTRTAADRCTQERIGFFVKDLL 219

eIF4E Human GLPPKI--------------------------------------VIGYQSHADTATKSGS 209

eIF4E1 Tc GLPPAMKF--------------------------------------IFTPHS-------S 258

eIF4E1 Tb GLLPTTKP--------------------------------------IFTAHGAS-----S 231

eIF4E1 Ba GIPSSATI--------------------------------------SFTKHEDK-----S 197

eIF4E1 Em NLTPIASI--------------------------------------VFTKHGDT-----S 220

eIF4E1 Lm SLAPIASM--------------------------------------VFTKHGEA-----S 214

eIF4E2 Em RLRFQ-KETNRNPISREHDALLSATRFQDRSSPAKTREVLGIPPTITYVSHRELMEAKQE 164

eIF4E2 Lm HLQPPSKEASRDGKSGEQDTLMEMPRSPDRSPVAKMREASGIPSAMTYVAHRDLMEAKQE 254

eIF4E2 Ba KPTDIQFGHTS-----------GTPTLQRLSGDEEDKKSFASDRVLPFMVHRDIMQAKMD 274

eIF4E2 Tc NLED---------------------------------------GVLQYLSHRELMQASKE 239

eIF4E2 Tb HLED---------------------------------------GSLQYFSHRELMQASEK 240

eIF4E Human TTKN----------RFVV--------- 217

eIF4E1 Tc TGK------------------------ 261

eIF4E1 Tb KA------------------------- 233

eIF4E1 Ba --------------------------- 197

eIF4E1 Em --------------------------- 220

eIF4E1 Lm --------------------------- 214

eIF4E2 Em FVKGGSSAAQTFRPKYTLVLDARADAV 191

eIF4E2 Lm FVKGGSSVAQAFRPKYTLAIDVRNEGV 281

eIF4E2 Ba SEAGA-VSTQSLQPRYRV--------- 291

eIF4E2 Tc GC-------WKVPPKYEL--------- 250

eIF4E2 Tb GS-------WDVPPLYQL--------- 251

**Supplementary Fig. S26.** **Sequence comparison of human eIF4E and selected Class I orthologs from Trypanosomatids.** Full-length proteins were compared.These proteins have been also termed Group 1 eIF4Es (32,33). Sequences were from Kinetoplastid Informatics Resources TriTrypDB (<https://tritrypdb.org>). Residues involved in the cap recognition (9-12) are indicated as follows: , W binding the guanine by  –  interactions; *G*, residue recognizing the guanine ring; *P*, residues interacting with the phosphate groups; *m*, W recognizing the cap methyl group. *Asterisks* indicate W43 and W56 of the human protein used to classify the eIF4E-family members into three classes (13). Residues identical to human eIF4E are shaded in black boxes. Conservative changes are in grey boxes. Gaps are represented by dashes. Purple boxes highlight conservation of phosphorylated residue S209 of human/mouse eIF4E (15,16). Phospho-mimicking E or D residues (18,19) are also highlighted in gray boxes. Human eIF4E (acc. numb.M15353) (20). Trypanosomatid species: Tc, *Trypanosoma cruzei* (acc. num. TcCLB.506127.170 and TcCLB.511353.40); Tb, *Trypanosoma brucei* (acc. num. Tb427tmp.18.0004 and Tb427.10.16070); Ba, *Blechomonas ayalai* (acc. num. Baya_063_0250 and Baya_133_0040); Em, *Endotrypanum monterogeii* (acc. num. EMOLV88_270020700 and EMOLV88_190018800); Lm, *Leishmania major* (acc. num. LmjF.27.1620 and LmjF.19.1500).

eIF4E Human --------------------------------MATVEPETTPTP------------NPPT 16

eIF4E3 Tc SNRIDSARQEDSFGST----------------SAADETSSNALQQ--------TGALGEA 227

eIF4E3 Tb --AIASPPEEDAVAAN----------------GGSVDASGAPSTQ--------NFTVGEI 211

eIF4E3 Ba -------RANDPHSSF----------------RCKSSSNTMGSGM--------EEEFPTA 126

eIF4E3 Em ----------------------------------GNEADKSEDSQ--------LDWLPEA 116

eIF4E3 Lm ----------------------------------KKEADENDDSQ--------LDWLPEA 113

eIF4E4 Tc --TTTSTRKNITQPALPLAVKANFTPTFAPIYVEKTEEEILEISKRSSLKVGAAAFVPRR 117

eIF4E4 Tb --VTTCTKSNGVRSATSPVPKAGSTPIVAEISVDKTDEEVLEISRCSSLKASAPAFLPRR 218

eIF4E4 Ba ---------------------HAATSSGPAMLGGKTEREIVEISQKSSLKVEATPYVPKR 170

eIF4E4 Em --T-AAARR-----S-----LHNSPIMQPSRLGIKSAVEIEAISKSSALNAAAAAYVPQR 236

eIF4E4 Lm --P-GAVRR-----S-----LQNSPIIQPSRLSVKSASEIEAISKNSALNAAAAAYVPQR 235

*

* ****

eIF4E Human TEEEKTE-------SNQ-EVANPEHYIKHPLQNRWALWFFKNDKS----------KTWQA 58

eIF4E3 Tc VENDFSGSVLPSVFEVS-VVAKPPPAEAIRFNTVWALHADDHPTP--------FGAPLAY 278

eIF4E3 Tb VENDFSGSMLPSLFQVE-VLDKPAPAEPVRFNTVWALYADEHPTP--------FGAPLAY 262

eIF4E3 Ba TAVDWTDSKLPALFGCH-NTSAMATSGPMRFNSAWELYADDHSTATTPLQSGNTAATMSF 185

eIF4E3 Em QAVNWCESKLPKLFGCH-NTAAKATSTAIPLHASWDLYADDHQGSSNSSSNSSPGSTTSF 175

eIF4E3 Lm QPTDWSESKLPKLFGCH-NTAAKATSSAIPLHASWDLYADDHQGSSNMASNSSPTSTMSF 172

eIF4E4 Tc TLNR-VMIAKPSPLSLTPA------TGEMTLGDLWCLFYLPAGLG-------ECIRENTY 163

eIF4E4 Tb TLNR-SNMTKPSPFTLTPD------SGDMRFGDPWCLFYLPVGGP-------DSTRESTY 264

eIF4E4 Ba TVKR-MLLTSRNSPTMSP-TDAAKDPSSMSFTDLWCLFYLSSSFG-------ESIKEETY 221

eIF4E4 Em TLTR-VVLAHPSPLALAPSEDPAKDNIDMMLDDLWCLFYLPSKLG-------ENIKEEDY 288

eIF4E4 Lm TLAR-VVLTQPSPLALAPSEDPAKNNIEMMLDDLWCLFYLPTTLG-------ENIKEEDY 287

**G P**

eIF4E Human NLRLISKFDTVEDFWALYNHIQLSSNLMPGCDYSLFKDGIEPMWEDEKNKRGGRWLITLN 118

eIF4E3 Tc DPVLVHLVGDVECFWRLWRYLPPPSALLPAFTYHWFRRDIKPNWEHARNKNGGTITIVIF 338

eIF4E3 Tb HPVLVHLVGDVECFWRLWRHLPPPSTLLPAFTYHWFRRDIRPNWEHTRNKNGGTITFVIF 322

eIF4E3 Ba EPILISTVGDVEAFWRLWRYSPPPSSCPSPFTYSFFRKDVKPEWEHPRNRKGGTITVVIF 245

eIF4E3 Em EPTFVANVGDVERFWRLWRYLPSPSSLPTMYTYSWFRKDIRPEWEHPRNKKGGTISIVVY 235

eIF4E3 Lm EPIFVSNVGDVESFWRLWRYLPAPSALPTVYTYSWFRKDIKPEWEHPRNKKGGTISIVVF 232

eIF4E4 Tc DPTLVFRVDSVATFWKVFNNIPQPTEMKIG-TLYFFRDGINPKWEDPGNRDGGILKMKLD 222

eIF4E4 Tb DPTLVFRMDCISSFWKVFNNIPEPTRMCAG-TLYLFRDGINPKWEDLRNRDGGIVRAKVR 323

eIF4E4 Ba NPTLVFRLENIPTFWKVFNNIPLPSNMQLS-TLYLFRDGIDPKWEDPANRNGGIVKVKVP 280

eIF4E4 Em NPTLVFRVDSIPTFWRVLNNIAAPSELQLS-TLYLFRDGIDPKWEDAANRDGGIVKVKVT 347

eIF4E4 Lm NPTLVFRVDSILTFWRVVNNIAAPSELQLS-TLYLFRDGIDPKWEDPANRDGGIVKVKAT 346

**P** **P** **m**

eIF4E Human KQQR-----RSDLDRFWLETLLCLIGESFDDY-SDDVCGAVVNVRAK-GDKIAIWTTECE 171

eIF4E3 Tc DRDKPGQNSKQTMDDAFMTMLMACCGETLAES-TTNLNGIMLKVRQNKPTTVQIWTASSD 397

eIF4E3 Tb DRDKPGLNNKQTMDDAFMAMLMACSGESLAES-TTNLNGVMLKVRQNKPTTLQIWTASSD 381

eIF4E3 Ba DRDRPGLNDKQVLDDVFMATLLGCVGESFTEG-TSTLNGVMLKVRQNKPVAMQIWTAHSE 304

eIF4E3 Em DRDRPGLNDKQVLDDVFMAMLLGAVGESFHEC-NTTLNGLMLKVRSNKPVTLQLWTAHSE 294

eIF4E3 Lm DRDRSGLSDKQVLDDVFMAMLVGAVGESFHEC-STTLNGIMLKVRSNKPVTLQLWTAHSE 291

eIF4E4 Tc ---------SHCINDAWVYLLCRTIGESWSRSVRDTVNGVALKARER-AYLLEVWVTEQS 272

eIF4E4 Tb ---------PQVVDDAWLHLLCRTVGESWSRSVRNSVNGIALKVRAA-AFMLEVWVTEQT 373

eIF4E4 Ba ---------SHLVNEAWELLLCRTVGDTWSPPVRDAVTGVALKVRER-AYLLEVWVKKQS 330

eIF4E4 Em ---------AAQVDEAWELLLCRTIGDSWSPSVRETVNGVVLKVRER-AYWLELWVTKDS 397

eIF4E4 Lm ---------AAQVDEAWELLLCRTIGDSWSPSVRETVNGVVLKVRER-AYWLELWVTKNS 396

eIF4E Human NREAVTH---IGRVYKERLGLPPKIVIGYQSHADTATKSG-STT--KNRFVV---------- 217

eIF4E3 Tc QRKLKALAGSLRNLLEKIIGAKPLQKLEYFSHQQTQVGGAGSL--AGRMKGKPTRITPDFTL 457

eIF4E3 Tb ELKLRSLARSLRTLLEKVIGPKPLQKLEYFSHQRTQVGAPGSL--AGRMKGKPSRITPDFTL 441

eIF4E3 Ba VGKLKAFANSLRDTLSRVMGGKPLQKIEYYSHHQRRPG-DDSL--ASRVYAKP-KSTPDYVL 362

eIF4E3 Em VSKLKTFANSVRDVLGKVMGPKTLQKMEYYSHHQKQAA-TNSL--AARMKGKT-KISPDHTF 352

eIF4E3 Lm VGKLKAFANSVRDTLTKIMGAKTLQKLEYYSHHQKQAA-TNSL--AARMKGKT-KISPDHTM 349

eIF4E4 Tc AELMMD----ISELLRPLLGDV--FSVFYAPHSVTQERAAAAAL--AEKKRSRNNRRRW--- 323

eIF4E4 Tb SELMSD----ISELLHKFLGDA--FQVPYIPHSVAQERAATNAAALAVKEKKNRGNRRLW-- 427

eIF4E4 Ba PELMKD----IADLWHSLLGGA--FAAVYYSHSAIQERAATAALDKIRKQKKR---R----- 378

eIF4E4 Em GALQKD----LADLWHPVLGAS--FSTTYLTHAVMQERSHAAAALAAEKQKKN---RRRY-- 448

eIF4E4 Lm SALQKD----LAELWHPILGAS--FATTYLTHAMMQERSHAAAALAAEKQKKN---RRRY-- 447

**Supplementary Fig. S27.** **Sequence comparison of human eIF4E and selected Class III orthologs from Trypanosomatids.** Full-length proteins were compared.These proteins have been also termed Group 2 eIF4Es (32,33). Sequences were from Kinetoplastid Informatics Resources TriTrypDB (<https://tritrypdb.org>). Residues involved in the cap recognition (9-12) are indicated as follows: , residues binding the guanine by  –  interactions; *G*, residue recognizing the guanine ring; *P*, residues interacting with the phosphate groups; *m*, W recognizing the cap methyl group. *Asterisks* indicate W43 and W56 of the human protein used to classify the eIF4E-family members into three classes (13). Residues identical to human eIF4E are shaded in black boxes. Conservative changes are in grey boxes. Gaps are represented by dashes. T-Class III specific residues are highlighted in red. Purple boxes highlight conservation of phosphorylated residue S209 of human/mouse eIF4E (15,16). Phospho-mimicking E or D residues (18,19) are also highlighted in gray boxes. Human eIF4E (acc. numb.M15353) (20). Trypanosomatid species: Tc, *Trypanosoma cruzei* (acc. num. TcCLB.508827.30 and TcCLB.421959.10); Tb, *Trypanosoma brucei* (acc. num. Tb427tmp.01.3630 and Tb427.06.1870); Ba, *Blechomonas ayalai* (acc. num. Baya_050_0340 and Baya_105_0020); Em, *Endotrypanum monterogeii* (acc. num. EMOLV88_280026900 and EMOLV88_300011200); Lm, *Leishmania major* (acc. num. LmjF.28.2500 and LmjF.30.0450).

*

eIF4E Human ---MATVEPETT--PTPNPPTTEEEKTESNQEVANPEHYIKHPLQNRWALWFFKNDKSKT 55

eIF4E5 Tc ------------------------------------MKETAHALKDPWFLSYIPQLTPDT 24

eIF4E5 Tb ------------------------------------MEEESHALKDPWFVSYIPQLTTEI 24

eIF4E5 Ba -------------------------------------MATTHQLKAGWFVSFLPLLTKET 23

eIF4E5 Em -------------------------------------MSATHALRDKWFVSFLPLLTADM 23

eIF4E5 Lm --------------------------------MKSATMSATHALRDKWFVSFLPLLTADM 28

eIF4E6 Ba ----------------------------MTSPNEPPAQPKQHPLRDKWFLFYNPSGKSGS 32

eIF4E6 Em -------------------------MSDSSSIREASQASALHPLKSKWFVFYIPASKGNE 35

eIF4E6 Lm MLWRGAVERITRTSNLKSEHICWASMADSNPTKEGTSSAPLHPLKDKWFVFYIPASKGNE 60

eIF4E6 Tc --------------------------------MSAQTSSQPHPLKDRWFVTYFPFVRQKK 28

eIF4E6 Tb --------------------------------MAAEATEKPHPLKDRWFVSYFPVVKQKK 28

* *

** **

Human eIF4E --------W----QANLRLISKFDTVEDFWALYNHIQLSSNLMPGCDYSLFKDGIE-PMW 102

eIF4E5 Tc VKYDFKGDWNKAKQALQQPLDYIRTVEEFWSTINSLPKLHQLGNGSTFIFARNNVD-ASY 83

eIF4E5 Tb VKNNYEGDWNLAKEALQQPLDYVRTVEEFWSTLNSLPKLHQLESSSTFVFARNNVD-ASY 83

eIF4E5 Ba VEQEFKGDWKLAAKERRQKLDWVYSVEELWSTVNSLPKLQHLEIGSTLIFSRHDKD-PSF 82

eIF4E5 Em VNTDFKGNWQLAAQERTQKLDWITSVEELWSTMNSLPKIHQLGMGSTLIFARNNKEPPSY 83

eIF4E5 Lm VNTDYKGNWQLAAQERTQKLDWITSVEELWSTMNSLPKVHQLGMGSTLIFARNNKEPPSY 88

eIF4E6 Ba -----D--F----SDVFDELDNVTTIEEVFASLNTLPYPSLLPKDDNIIFSRNKIT-PKF 80

eIF4E6 Em --------Y----ESEAKELGYVTTIEEVYSTINTLPPITLLPFDDNLVFSRNKIA-PQF 82

eIF4E6 Lm --------Y----EHETKELGYVSTIEEVYSTINTLPPITLLPNDDNLVFSRNKIE-PQF 107

eIF4E6 Tc KGKD----FD--EQQKPKELDWVTTAEELYATINSFPSLVLLPSDDNLVFARNKVE-PYF 81

eIF4E6 Tb --------FSKDEEQKGVELDWVSTAEELHATINAFSPLTLXPPDDNLVFAREKVE-PFF 80

**G P** **P**

Human eIF4E EDEKNKRGGRWLITLNKQQRRSDLDRFWLETLLCLIGESFDD-YS--DDVCGAVVNVRAK 159

TcEIF4E5 EAFP--NGTRVLVDLYKASVAEK---GMDFVLSSVLGEGLTYDVCNGKTVCDV-VRLSSR 137

TbEIF4E5 EAFP--NGTRIIVDIRKAAMAEK---ATAVILSSVIGESVSQEVCGGKPICDV-LRLSSR 137

BaEIF4E5 ETFP--NGSRVVINLHKPPAQEK---GLELVLASVVGELVAESTSKGQPVCDV-IRIAAR 136

EmEIF4E5 EAYP--NGSRIMINLLRPPTTDA---GLELVLAVVMGETVAEKASDGQPVCDV-LRIAAR 137

LmEIF4E5 EAYP--NGSRIMINLLKPPTTDA---GLELVLAVVMGETAAEKASDGKPVCDV-LRIAAR 142

BaEIF4E6 ESFP--GGHRLSIFTKTRVQSDE---VLYRTIAAVMGESI-TKECQGESVVDV-IRITPK 133

EmEIF4E6 ESFP--GGTRFCIFCKTKTQCRE---AMTYVLAAVLGEAISRDACNGEPLCDI-VRVGHK 136

LmEIF4E6 ESFP--GGMRFSIFCKTKTQCRE---ALTYVVAVVLGEAIGRDACKGECVCDI-VRIGHK 161

TcEIF4E6 ENFP--EGDRVCVFTRTKAQSEQ---AVVLVLAAVMGEHL-RSVTESECVADV-VRIAHK 134

TbEIF4E6 ENFP--NGMRVSVFTRTKVQATQ---AVPLVLAAVMGEHL-RTVTDGPSHADV-VRIAHK 133

**P** **m**

Human eIF4E -------GDKIAIWTTECENREAVTH-IGRVYKERLGLPPKIVIGYQSHADTATKS--GS 209

eIF4E5 Tc PNQESPELVRLEVWLSDQLYAKDVIPYIRRGLNEA-GLSFTDFIMGESTFEKDKKKPSVS 196

eIF4E5 Tb PNKESPELVRLEVWLSDQTYGKAVLAYVRKALNDV-GMSQPHVIFGESLFEKEKKKKGK- 195

eIF4E5 Ba RSREFSDLVRVEIWLNDALYTKSVSSYFQTLFKER-EIPPTAYSIQDNVFTEPTVSTPMS 195

eIF4E5 Em PTRDNSEQIRVEVWLSDSSRSHAVAEFLADALKEK-GLAANSYNISEATFDAVVPSKDKK 196

eIF4E5 Lm PSREHSEQIRVEVWLSDSTRSHAVAEFLAEAMRAK-GLAANSYNIAEASFDAAAPGKDKK 201

eIF4E6 Ba PGTVFKDALRLEVWLHKSPYAKTLEEYFNNLFRSTPGVSVMSRDFDEKGSRTKNPAPRTS 193

eIF4E6 Em SSAMYKESVRIEVWAHQSKHNAAMEKYLMSTLSMIPGISVSVRPLK-------------- 182

eIF4E6 Lm GSAMYKESVRIEVWAHQSPYNTAMEKYLVSTLSAIPGITVSARPFK-------------- 207

eIF4E6 Tc PGNVYPESLRVEVWLHKSDFCEKVVQYFVDLFKTYPGIRVARRPISSEVTAD-------- 186

eIF4E6 Tb PGTVYPESLRVEVWLRDRSKVDAVTKYFSEMLAPHPGIRVAGRPINAEGEEAK------- 189

Human eIF4E TTKNRFVV----------- 217

TcEIF4E5 GAKN--------------- 200

TbEIF4E5 ------------------- 195

BaEIF4E5 PP-------TSVPCAEEQK 207

EmEIF4E5 APLAASTLITSLPSMVKD- 214

LmEIF4E5 VLAASTMPSPSSPPMVKD- 219

BaEIF4E6 ------------------- 193

EmEIF4E6 ------------------- 182

LmEIF4E6 ------------------- 207

TcEIF4E6 ------------------- 186

TbEIF4E6 ------------------- 189

**Supplementary Fig. S28.** **Sequence comparison of human eIF4E and selected Class IV orthologs from Trypanosomatids.** Full-length proteins were compared.These proteins have been also termed Group 3 eIF4Es (32,33). Sequences were from Kinetoplastid Informatics Resources TriTrypDB (<https://tritrypdb.org>). Residues involved in the cap recognition (9-12) are indicated as follows: , residues binding the guanine by  –  interactions; *G*, residue recognizing the guanine ring; *P*, residues interacting with the phosphate groups; *m*, W recognizing the cap methyl group. *Asterisks* indicate W43 and W56 of the human protein used to classify the eIF4E-family members into three classes (13). Residues identical to human eIF4E are shaded in black boxes. Conservative changes are in grey boxes. Gaps are represented by dashes. T-Class IV specific residues are highlighted in red. Purple boxes highlight conservation of phosphorylated residue S209 of human/mouse eIF4E (15,16). Human eIF4E (acc. numb.M15353) (20). Trypanosomatid species: Tc, *Trypanosoma cruzei* (acc. num. TcCLB.510293.30 and TcCLB.508207.180); Tb, *Trypanosoma brucei* (acc. num. Tb427.10.5020 and Tb427.07.1670); Ba, *Blechomonas ayalai* (acc. num. Baya_118_0170 and Baya_023_0230); Em, *Endotrypanum monterogeii* (acc. num. EMOLV88_360011000 and EMOLV88_260006900); Lm, *Leishmania major* (acc. num. LmjF.36.0590 and LmjF.26.0240).

**
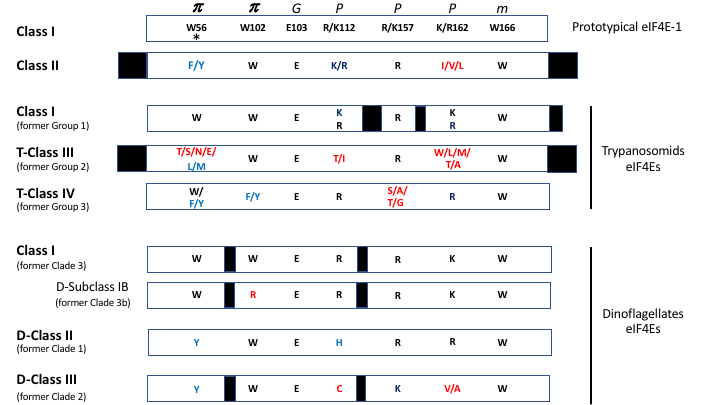
**

**Supplementary Fig. S29. Scheme representing the core of prototypical eIF4Es and eIF4Es from the indicated protists.** Class I eIF4E from metazoan, terrestrial plants and yeast is represented. Numbering is from human protein (20). Amino acid residues directly contacting the mRNA cap structure are indicated (9-12). Trypanosomids and dinoflagellates eIF4Es are indicated with the prefix T- and D-, respectively. Conservative substitutions with respect to the prototypical proteins are in blue. Non-conservative changes are in red. An asterisk indicates W56 used to classify the eIF4E-family members into three classes (13). Stretches of amino acid insertions and extensions are depicted as black boxes (not to scale). Trypanosomids nomenclature in Groups is according to (32,33). Dinoflagellates nomenclature in Clades is according to (34,35).

**Supplementary Table S2.** Conservation of phosphorylatable residues^a,b^ in the fungal eIF4E orthologs as reported for the *S. cerevisiae* S2, S15, and S28 (14,18) and human/mouse S209 (15,16) eIF4Es^c^.

| ***Phylum*** | **Class** | **S2** | **S15** | **S28** | **S209** | **Suppl.**  **Fig.** |
| --- | --- | --- | --- | --- | --- | --- |
| *Ascomycota* | **I** | NC | PC | NC | HC | S9 |
|  | **II** | NC | PC | PC | PC | S10 |
|  | **F-IV** | PC | PC | HC | NC | S11 |
|  | **V** | PC | HC | PC | HC | S12 |
| *Basidiomycota* | **I** | HC | PC | PC | PC | S13 |
|  | **II** | PC | PC | PC | HC | S14 |
|  | **F-VI** | NC | NC | NC | NC | S15 |
| *Chytridiomycota* | **I** | NC | PC | HC | HC | S16 |
|  | **II** | NC | PC | HC | HC | S16 |
| *Rozellomycota* | **I** | PC | NC | NC | HC | S17 |
|  | **II** | PC | NC | NC | HC | S17 |
| *Mucoromycota* | **I** | PC | PC | NC | PC | S18 |
|  | **II** | PC | NC | PC | HC | S19 |
|  | **F-VI** | PC | NC | PC | NC | S20 |
|  | **F-VII** | NC | NC | NC | NC | S20 |
| *Zoopagomycota* | **I** | HC | HC | PC | HC | S7 |
|  | **II** | NC | NC | NC | HC | S8 |

^a^ *NC*, non-conserved; *PC*, partially conserved; *HC*, highly conserved.

^b^ S, T and the phospho-mimicking amino acids D and E are considered conservative changes.

^C^ Data from the cap-binding pocket shown in Fig. 3 (main text).

**Supplementary Table S3.** Distances (Å units) between the cap-interacting amino acids of eIF4E and the cap moieties of the indicated species^1^.

|  | **Cap moiety** | | | | | | |
| --- | --- | --- | --- | --- | --- | --- | --- |
|  | **- stacking** | **- stacking** | **Guanine** | **Phosphate** | **Phosphate** | **Phosphate** | **7-methyl** |
| **Species** |  | | | | | | |
| *Saccharomyces cerevisiae* | 2.1 | 2.4 | 1.7 | 4.1 | 3.6 | 3.0 | 3.2 |
| *Cryptococcus gatti* | 4.1 | 2.8 | 1.5 | 5.9 | 3.9 | 3.7 | 3.6 |
| *Mitosporidium daphnia* | 0.8 | 2.3 | 1.3 | 3.7 | 3.4 | 4.1 | 3.1 |
| *Nematocida major* | 3.3 | 3.4 | 2.2 | 4.1 | 2.7 | 4.5 | 3.9 |
| *Punctularia strigosozonata* | 1.8 | 3.4 | 2.3 | 4.1 | 3.5 | 5.3 | 4.0 |
| *Uncinocarpus reesii* | 2.1 | 3.2 | 0.9 | 7.7 | 2.4 | 4.1 | 3.8 |
| *Ascochyta rabiei* | 0.5 | 2.3 | 1.5 | 8.6 | 2.2 | 4.2 | 3.8 |
| *Malassezia globosa* | 3.5 | 3.0 | 1.6 | 4.5 | 3.6 | 3.0 | 3.8 |
| *Gilbertella persicaria* | 2.7 | 2.9 | 1.9 | 7.9 | 2.1 | 4.4 | 4.0 |
| *Gamisella multidivaricata* | 3.9 | 2.4 | 0.4 | 8.6 | 1.8 | 4.4 | 4.3 |

^1^ Data from the cap-binding pocket shown in Fig. 3 of the main text.

**Supplementary Table S4.** Plasmids used in this study.

| **Plasmid** | **Description** | **Source** |
| --- | --- | --- |
| pVT-URA3 | Contains the *URA3* gene as an auxotrophic marker and the *ADH* (*Alcohol dehydrogenase*) promoter for constitutive expression in *S. cerevisiae*. | (36) |
| pESC-TRP | Contains the *TRP1* gene as an auxotrophic marker and the Gal10 promoter for regulated gene expression with galactose in *S. cerevisiae*. | GeneScript |
| <pVT-URA3-eIF4E> | It expresses *S. cerevisiae* eIF4E under the constitutive *ADH* promoter. | (37) |
| pESC-TRP-eIF4E-HA  S. cerevisiae. | *S. cerevisiae CDC33-HA* cDNA cloned onto pESC-*TRP1*. Gene NP_014502.1; Genome GCF_000146045; *Phylum Ascomycota*; Class I eIF4E. | This study |
| pESC-TRP-eIF4E-HA  C. gatti. | *Cryptococcus gatti eIF4E-HA* cDNA cloned onto pESC-*TRP1*. Gene XP_003194871.1; Genome GCF_000185945; *Phylum Basidiomycota*; F-Subclass IA eIF4E. | This study |
| pESC-TRP-eIF4E-HA  M. daphnia. | *Mitosporidium daphnia eIF4E-HA* cDNA cloned onto pESC-*TRP1*. Gene XP_013237606.1; Genome GCF_000760515; *Phylum Rozellomycota;* F-Subclass IB eIF4E. | This study |
| pESC-TRP-eIF4E-HA  N. major | *Nematocida major eIF4E-HA* cDNA cloned onto pESC-*TRP1.* GeneXP_047772122.1; Genome GCF_021653875; *Phylum Rozellomycota*; Class II eIF4E. | This study |
| pESC-TRP-eIF4E-HA  P. strigosozonata. | *Punctularia strigosozonata eIF4E-HA* cDNA cloned onto pESC-*TRP1*. Gene XP_007382918.1; Genome GCF_000264995; *Phylum Basidiomycota*; Class II eIF4E. | This study |
| pESC-TRP-eIF4E-HA  U. reesii. | *Uncinocarpus reesii eIF4E-HA* cDNA cloned onto pESC-*TRP1*. Gene XP_002543851.1; Genome GCF_000003515; *Phylum Ascomycota*; F-Class IV eIF4E. | This study |
| pESC-TRP-eIF4E-HA  A. rabiei. | *Ascochyta rabiei eIF4E-HA* cDNA cloned onto pESC-*TRP1*. Gene XP_038798412.1; Genome GCF_004011695; *Phylum Ascomycota*; F-Class V eIF4E. | This study |
| pESC-TRP-eIF4E-HA  M. globosa. | *Malassezia globosa eIF4E-HA* cDNA cloned onto pESC-*TRP1*. Gene XP_001729563.1; Genome GCF_000181695; *Phylum Basidiomycota*; F-Class VI eIF4E. | This study |
| pESC-TRP-eIF4E-HA  G. persicaria. | *Gilbertella persicaria eIF4E-HA* cDNA cloned onto pESC-*TRP1*. Gene XP_051439445.1; Genome GCF_025201335; *Phylum Mucoromycota*; F-Class VII eIF4E. | This study |
| pESC-TRP-eIF4E-HA  G. multidivaricata. | *Gamisella multidivaricata eIF4E-HA* cDNA cloned onto pESC-*TRP1*. Gene XP_051416061.1; Genome GCF_025024155; *Phylum Mucoromycota*; F-Class VII eIF4E. | This study |

**Supplementary References**

1. Haft, D. H., Badretdin, A., Coulouris, G., DiCuccio, M., Durkin, A. S., Jovenitti, E., Li, W., Mersha, M., O'Neill, K. R., Virothaisakun, J., and Thibaud-Nissen, F. (2024) RefSeq and the prokaryotic genome annotation pipeline in the age of metagenomes. *Nucleic Acids Res.* **52**, D762-D769

2. Buchfink, B., Reuter, K., and Drost, H. G. (2021) Sensitive protein alignments at tree-of-life scale using DIAMOND. *Nat. Methods* **18**, 366-368

3. Mistry, J., Chuguransky, S., Williams, L., Qureshi, M., Salazar, G. A., Sonnhammer, E. L. L., Tosatto, S. C. E., Paladin, L., Raj, S., Richardson, L. J., *et al.* (2021) Pfam: The protein families database in 2021. *Nucleic Acids Res.* **49**, D412-D419

4. Hauser, M., Steinegger, M., and Soding, J. (2016) MMseqs software suite for fast and deep clustering and searching of large protein sequence sets. *Bioinformatics* **32**, 1323-1330

5. Kumar, S., Stecher, G., and Tamura, K. (2016) MEGA7: Molecular Evolutionary Genetics Analysis version 7.0 for bigger datasets. *Mol. Biol. Evol.* **33**, 1870-1874

6. Saitou, N., and Nei, M. (1987) The neighbor-joining method: A new method for reconstructing phylogenetic trees. *Mol. Biol. Evol.* **4**, 406-425

7. Jumper, J., Evans, R., Pritzel, A., Green, T., Figurnov, M., Ronneberger, O., Tunyasuvunakool, K., Bates, R., Žídek, A., Potapenko, A., Bridgland, A., Meyer, C., Kohl, S. A. A., Ballard, A. J., Cowie, A., Romera-Paredes, B., Nikolov, S., Jain, R., Adler, J., Back, T., Petersen, S., Reiman, D., Clancy, E., Zielinski, M., Steinegger, M., Pacholska, M., Berghammer, T., Bodenstein, S., Silver, D., Vinyals, O., Senior, A. W., Kavukcuoglu, K., Kohli, P., and Hassabis, D. (2021) Highly accurate protein structure prediction with AlphaFold. *Nature* **596**, 583–589

8. Grüner, S., Weber, R., Peter, D., Chung, M. Y., Igreja, C., Valkov, E., and Izaurraalde, E. (2018) Structural motifs in eIF4G and 4E-BPs modulate their binding to eIF4E to regulate translation initiation in yeast. *Nucleic Acid Res.* **46**, 6893-6908

9. Marcotrigiano, J., Gingras, A. C., Sonenberg, N., and Burley, S. K. (1997) Cocrystal structure of the messenger RNA 5' cap-binding protein (eIF4E) bound to 7-methyl-GDP. *Cell* **89**, 951-961

10. Matsuo, H., Li, H., McGuire, A. M., Fletcher, C. M., Gingras, A. C., Sonenberg, N., and Wagner, G. (1997) Structure of translation factor eIF4E bound to m7GDP and interaction with 4E-binding protein. *Nat. Struct. Biol.* **4**, 717-724

11. Tomoo, K., Shen, X., Okabe, K., Nozoe, Y., Fukuhara, S., Morino, S., Ishida, T., Taniguchi, T., Hasegawa, H., Terashima, A., Sasaki, M., Katsuya, Y., Kitamura, K., Miyoshi, H., Ishikawa, M., and Miura, K. (2002) Crystal structures of 7-methylguanosine 5'-triphosphate (m(7)GTP)- and P(1)-7-methylguanosine-P(3)-adenosine-5',5'-triphosphate (m(7)GpppA)-bound human full-length eukaryotic initiation factor 4E: biological importance of the C-terminal flexible region. *Biochem. J.* **362**, 539-544

12. Gross, J. D., Moerke, N. J., von der Haar, T., Lugovskoy, A. A., Sachs, A. B., McCarthy, J. E. G., and Wagner, G. (2003) Ribosome loading onto the mRNA cap is driven by comformational coupling between eIF4G and eIF4E. *Cell* **115**, 739–750

13. Joshi, B., Lee, K., Maeder, D. L., and Jagus, R. (2005) Phylogenetic analysis of eIF4E-family members. *BMC Evol. Biol.* **5**, 48

14. Zanchin, N. I., and McCarthy, J. E. G. (1995) Characterization of the in vivo phosphorylation sites of the mRNA.cap-binding complex proteins eukaryotic initiation factor-4E and p20 in *Saccharomyces cerevisiae* *J. Biol. Chem.* **270**, 26505-26510

15. Joshi, B., Cai, A. L., Keiper, B. D., Minich, W. B., Mendez, R., Beach, C. M., Stepinski, J., Stolarski, R., Darzynkiewicz, E., and Rhoads, R. E. (1995) Phosphorylation of eukaryotic protein synthesis initiation factor 4E at Ser-209. *J. Biol. Chem.* **270**, 14597-14603

16. Flynn, A., and Proud, C. G. (1995) Serine 209, not serine 53, is the major site of phosphorylation in initiation factor eIF-4E in serum-treated Chinese hamster ovary cells. *J. Biol. Chem.* **270**, 21684-21688

17. Lachance, P. E. D., Miron, M., Raught, B., Sonenberg, N., and Lasko, P. (2002) Phosphorylation of eukaryotic translation initiation factor 4E is critical for growth. *Molecular & Cellular Biology* **22**, 1656-1663

18. Studer, R. A., Rodriguez-Mias, R., Haas, K. M., Hsu, J., Viéitez, C., Solé, C., Swaney, D. L., Stanford, L. B., Liachko, I., Böttcher, R., and al., e. (2016) Evolution of protein phosphorylation across 18 fungal species. *Science* **354**, 229-232

19. Ross-Kaschitza, D., and Altmann, M. (2020) eIF4E and interactors from unicellular eukaryotes. *Int. J. Mol. Sci.* **21**, 2170

20. Rychlik, W., Domier, L. L., Gardner, P. R., Hellmann, G. M., and Rhoads, R. E. (1987) Amino acid sequence of the mRNA cap-binding protein from human tissues. *Proc. Natl. Acad. Sci. U. S. A.* **84**, 945-949

21. Rychlik, W., and Rhoads, R. E. (1992) Nucleotide sequence of rabbit eIF-4E cDNA. *Nucleic Acid Res.* **20**, 6415

22. Hernández, G., Altmann, M., Sierra, J. M., Urlaub, H., Corral, R. D., Schwartz, P., and Rivera-Pomar, R. (2005) Functional analysis of seven genes encoding eight translation initiation factor 4E (eIF4E) isoforms in *Drosophila*. *Mech. Dev.* **122**, 529–543

23. Lasko, P. (2000) The *Drosophila melanogaster* genome: translational factors and RNA binding proteins. *J. Cell Biol.* **150**, F51–F56

24. Metz, A. M., Timmer, R. T., and Browning, K. S. (1992) Isolation and sequence of a cDNA encoding the cap binding protein of wheat eukaryotic protein synthesis initiation factor 4F *Nucleic Acid Res.* **20**, 4096

25. Altmann, M., Handschin, C., and Trachsel, H. (1987) mRNA cap-binding protein: cloning of the gene encoding protein synthesis initiation factor eIF4E from *Saccharomyces cerevisiae*. *Mol. Cell. Biol.* **7**, 998–1003

26. Brenner, C., Nakayama, N., Goebl, M., Tanaka, K., Toh-e, A., and Matsumoto, K. (1988) *CDC33* encodes mRNA cap-binding protein eIF-4E of *Saccharomyces cerevisiae*. *Mol. Cell Biol.* **8**, 3556–3559

27. Ptushkina, M., Fierro-Monti, I., Heuvel, J. V. D., Vasilescu, S., Birkenhäger, R., Mita, K., and McCarthy, J. E. G. (1996) *Schizosaccharomyces pombe* has a novel eukaryotic Initiation Factor 4F complex containing a cap-binding protein with the human eIF4E C-terminal motif KSGST. *J. Biol. Chem.* **271**, 32818–32824

28. Ptushkina, M., Berthelot, K., von der Haar, T., Geffers, L., Warwicker, J., and McCarthy, J. E. (2001) A second eIF4E protein in *Schizosaccharomyces pombe* has distinct eIF4G-binding properties. *Nucleic Acids Res.* **29**, 4561-4569

29. Rom, E., Kim, H. C., Gingras, A. C., Marcotrigiano, J., Favre, D., Olsen, H., Burley, S. K., and Sonenberg, N. (1998) Cloning and characterization of 4E-HP, a novel mammalian eIF4E-related cap-binding protein. *J. Biol. Chem.* **273**, 13104–13109

30. Keiper, B. D., Lamphear, B. J., Deshpande, A. M., Jankowska-Anyszka, M., Aamodt, E. J., Blumenthal, T., and Rhoads, R. E. (2000) Functional characterization of five eIF4E isoforms in *Caenorhabditis elegans*. *J. Biol. Chem.* **275**, 10590-10596

31. Ruud, K. A., Kuhlow, C., Goss, D. J., and Browning, K. S. (1998) Identification and characterization of a novel cap-binding protein from *Arabidopsis thaliana*. *J. Biol. Chem.* **273**, 10325-10330

32. Das, S. (2021) Taking a re-look at cap-binding signatures of the mRNA cap-binding protein eIF4E orthologues in trypanosomatids. *Mol. Cell. Biochem.* **476**, 1037-1049

33. Freire, E. R., Sturm, N. R., Campbell, D. A., and De Melo Neto, O. P. (2017) The role of cytoplasmic mRNA cap-binding protein complexes in *Trypanosoma brucei* and other Trypanosomatids. *Pathogens* **6**, 55

34. Roy, S. W., Jagus, R., and Morse, D. (2018) Translation and translational control in dinoflagellates. *Microorganisms* **6**, 30

35. Jones, G. D., Williams, E. P., Place, A. R., Jagus, R., and Bachvaroff, T. R. (2015) The alveolate translation initiation factor 4E family reveals a custom toolkit for translational control in core dinoflagellates. *BMC Evol. Biol.* **15**, 14

36. Vernet, T., Dignard, D., and Thomas, D. Y. (1987) A family of yeast expression vectors containing the fage f1 intergenic region. *Gene* **52**, 225-233

37. Altmann, M., Müller, P. P., Pelletier, J., Sonenberg, N., and Trachsel, H. (1989) A mammalian translation initiation factor can substitute for its yeast homologue *in vivo*. *J. Biol. Chem.* **264**, 12145-12147
